# Abstracts from the 8th International Congress of the Asia Pacific Society of Infection Control (APSIC): Bangkok, Thailand. 12-15 February 2017

**DOI:** 10.1186/s13756-017-0176-1

**Published:** 2017-02-22

**Authors:** Nantanit Sutthiruk, Mari Botti, Julie Considine, Andrea Driscoll, Ana Hutchinson, Kumthorn Malathum, Cucunawangsih Cucunawangsih, Veronica Wiwing, Vivien Puspitasari, Rathina Kumar Shanmugakani, Yukihiro Akeda, Takuya Kodera, Pitak Santanirand, Kazunori Tomono, Takayuki Yamanaka, Hiroyuki Moriuchi, Hiroyuki Kitajima, Yuho Horikoshi, Alyona Lavrinenko, Ilya Azizov, Nurlan Tabriz, Margulan Kozhamuratov, Yekatherine Serbo, Dahae Yang, Woonhyoung Lee, Il Kwon Bae, Jae Hyun Lee, Hyukmin Lee, Jung Ok Kim, Seok Hoon Jeong, Kyungwon Lee, Thiba Peremalo, Priya Madhavan, Sharina Hamzah, Leslie Than, Eng Hwa Wong, Mohd Nasir Mohd Desa, Kee Peng Ng, Marionne Geronimo, Maria Fe Tayzon, Maria Jesusa Maño, Angela Chow, Pei-Yun Hon, Mar-Kyaw Win, Brenda Ang, Yee-Sin Leo, Angela Chow, Pei-Yun Hon, Tina See, Brenda Ang, Rocio Alvarez Marin, Marta Aires de Sousa, Nicolas Kieffer, Patrice Nordmann, Laurent Poirel, Wison Laochareonsuk, Sireekul Petyu, Pawin Wanasitchaiwat, Sutasinee Thana, Chollathip Bunyaphongphan, Woranan Boonsomsuk, Pakpoom Maneepongpermpoon, Silom Jamulitrat, Dorairajan Sureshkumar, Kalyanaraman Supraja, Soundararajan Sharmila, Cucunawangsih Cucunawangsih, Benny Setiawan, Nicolaski Lumbuun, Haruo Nakayama, Toshiko Ota, Naoko Shirane, Chikako Matuoka, Kentaro Kodama, Masanobu Ohtsuka, Silverose Ann Andales Bacolcol, Melecia Velmonte, Allan Alde, Keithleen Chavez, Arlene Joy Esteban, Aisa Jensen Lee, Tai-Chin Hsieh, Shio-ShinJean, Huey-Jen Huang, Shu-Ju Huang, Yu-Huan Huang, Pei-Chen Cheng, Su-Fang Yu, Shih-Ming Tsao, Yuan-Ti Lee, Chien-Feng Li, Min-Chi Lu, Nattapol Pruetpongpun, Thana Khawcharoenporn, Pansachee Damronglerd, Nuntra Suwantarat, Anucha Apisarnthanarak, Sasinuch Rutjanawech, Lisa Cushinotto, Patty McBride, Harding Williams, Hans Liu, Phan Thi Hang, Dinh Pham Phuong Anh, Ngai Le, Dung Khu, Lam Nguyen, Roel Beltran Castillo, Dorairajan Sureshkumar, Ram Gopalakrishnan, Venkatasubramanian Ramasubramanian, Subramanian Sreevidya, Ranganathan Jayapradha, Atsushi Umetsu, Tetsuhiro Noda, Kenyuu Hashimoto, Akihiro Hayashi, Mikie Kabashima, Ursula Jadczak, Knut Elvelund, Marit Johnsen, Bente Borgen, Egil Lingaas, Chia-Hua Mao, Fu-Chieh Chang, Chang-Pan Liu, Ru-Hui Chao, Fu-chieh Chang, Chang-pan Liu, Junpen Pawapotako, Chadanan Prasertpan, Wantanee Malaihuan, Phisit Uirungroj, Chadanan Prasertpan, Chalermpong Saenjum, Teerapat Ouirungrog, Phisit Uirungroj, Sue Borrell, Pauline Bass, Leon Worth, Zhao Xian-li, Li Xiao-long, Yao Xue-hua, Ren Wei, Zhang Xia Zeng, Man Ying Kong, Christopher Koon Chi Lai, Suet Yi Lee, Ngai Chong Tsang, M. M. O’Donoghue, M. V. Boost, L. K. P. Suen, G. K. Siu, K. W. Mui, C. K. C. Lai, D. N. C. Tsang, Yuka Sato, Mariko Tateishi, Mutsuko Mihashi, Jose Paulo Flor, Marko Bautista, V. Jay De Roxas, Justine Vergara, Nicolo Andrei Añonuevo, Marion Kwek, Jose Acuin, Anna Josea Sanchez, Avel Bathan, Jamilah Binte Jantan, Chua Chor Guek, Eu Chiow Kian, Pampe Anak Pirido, Nur Fadilah Binte Mohd Aron, Leah May Estacio, Francis Alvarez Palana, Michelle Gracia, Nur Syafiqah Binte Shamsuddin, Kersten Timbad Castro, Madonna Baloria, Faezah Binte Adam, Zhang Wei, Poh Bee Fong, Marimuthu Kalisvar, Angela Chow, Brenda Ang, I-Ju Chuang, Yi-ChunCho, Yu-Fen Chiu, Lung-Chih Chen, Yi-Chun Lin, Shao-Xing Dong, Yi-Chieh Lee, Hui-Chen Kuan, Hsin-Hua Lin, Chia-Chun Chi, Chin-Te Lu, Fu-chieh Chang, Chang-pan Liu, Tang Ya-Fen, Su Li-Hsiang, Liu Jien-Wei, Hsuehlan Chao, PinRu ChangChien, WeiFang Chen, ChungHsu Lai, Lutfe Ara, Syed Mohammad Niaz Mowla, Shaikh Mahmud Kamal Vashkar, Wai Fong Chan, Mabel Yin ChunYau, Karen Kam LingChong, Tze OnLi, Rajwinder Kaur, Ng Po Yan, Gloria Chor Shan Chiu, Christina W. Y. Cheung, Patricia T. Y. Ching, Radley H. C. Ching, Conita H. S. Lam, C. H. Kan, Shirley S. Y. Lee, C. P. Chen, Regina F. Y. Chan, Annie F. Y. Leung, Isadora L. C. Wong, S. S. Lam, Queenie W. L. Chan, Cecilia Chan, Rajwinder Kaur, Seyed Sadeq Seyed Nematian, Charles John Palenik, Mehrdad Askarian, Seyed Sadeq Seyed Nematian, Charles John Palenik, Nahid Hatam, Mehrdad Askarian, Itaru Nakamura, Hiroaki Fujita, Ayaka Tsukimori, Takehito Kobayashi, Akihiro Sato, Shinji Fukushima, Tetsuya Matsumoto, Jose Paulo Flor, Nicolo Andrei Añonuevo, Marko Bautista, Justine Vergara, V. James De Roxas, Marion Kwek, Jose Paulo Flor, Marko Bautista, Justine Vergara, V James De Roxas, Nicolo AndreiAñonuevo, Marion Kwek, Yeng May Ho, Jia Qi Kum, Bee Fong Poh, Kalisvar Marimuthu, Brenda Ang, Tzu-Yin Liu, Sin-Man Chu, Hui-Zhu Chen, Tun-chieh Chen, Yichun Chen, Ya-Ching Tsao, Sumawadee Skuntaniyom, Kumthorn Malathum, Pirawadee Tipluy, Sangwan Paengta, Ratchanee wongsaen, Sutthiphun thanomphan, Samettanet Tariyo, Buachan Thongchuea, Pattama Khamfu, Sutthiphan Thanomphan, Wipaporn Natalie Songtaweesin, Suvaporn Anugulruengkit, Rujipat Samransamruajkit, Darintr Sosothikul, Ornanong Tansrijitdee, Anry Nakphunsung, Patchareeyawan Srimuan, Jirachaya Sophonphan, ThanyaweePuthanakit, Kunyanut Payuk, Wilawan Picheansathian, Nongkran Viseskul, Elizabeth DeNardo, Rachel Leslie, Todd Cartner, Luciana Barbosa, Heinz-Peter Werner, Florian H. H. Brill, Julia Yaeko Kawagoe, Elizabeth De Nardo, Sarah Edmonds- Wilson, David Macinga, Patricia Mays-Suko, Collette Duley, Phan Thi Hang, Tran Thi Thuy Hang, Tran Thi My Hanh, Christopher Gordon, Dorairajan Sureshkumar, Roopa Durairaj, Anusha Rohit, Saujanya Saravanakumar, Jothymani Hemalatha, Ryuichi Hirano, Yuichi Sakamoto, Shoji Yamamoto, Naoki Tachibana, Miho Miura, Fumiyo Hieda, Yoshiro Sakai, Hiroshi Watanabe, Melecia Velmonte, Silverose Ann Bacolcol, Allan Alde, Keitleen Chavez, Arlene Joy Esteban, Aisa Jensen Lee, Angela Chow, Jia-Wei Lim, Pei-Yun Hon, Aung-Aung Hein, Grace Tin, Vanessa Lim, Brenda Ang, Angela Chow, Aung-Aung Hein, Jia-Wei Lim, Pei-Yun Hon, Vanessa Lim, Grace Tin, Brenda Ang, Angela Chow, Grace Tin, Aung-Aung Hein, Vanessa Lim, Jia-Wei Lim, Pei-Yun Hon, Brenda Ang, Huwi-chun Chao, Chiu-Yin Yeh, Mei-feng Lo, Huwi-chun Chao, Chonlada Piwpong, Songyos Rajborirug, Ploypailin Preechawetchakul, Yada Pruekrattananapa, Tharntip Sangsuwan, Silom Jamulitrat, Ratchanee Wongsaen, Sungwan Paengta, Napatnun Nilchon, Sutthipun Thanompan, Samattanet Tariyo, Ngai Le, Dung Khu, Svetlana Kolesnichenko, Ilya Azizov, Alyona Lavrinenko, Yerbol Tishkambayev, Alyona Lavrinenko, Ilya Azizov, Yerbol Tishkambayev, Asylkhan Alibecov, Svetlana Kolesnichenko, Yekaterina Serbo, Youngwon Nam, Jae Hyeon Park, Yun Ji Hong, Taek Soo Kim, Jeong Su Park, Kyoung Un Park, Eui-Chong Kim, Samuel Abumhere Aziegbemhin, Onaiwu Enabulele, Yao-Shen Tung, An-Chi Chen, Shen-Min Huang, Yui-Yein Yang, Li-Hung Wu, Chin-cheng Lin, Fu-chieh Chang, Chang-pan Liu, Tzu Hao Lien, Jia Hao Chang, Yu Shan Huang, Yi Shun Chen, Chalermpong Saenjum, Sasithorn Sirilun, Teerapat Ouirungrog, Phisit Ouirungroj, Suwanna Trakulsomboon, Patcharee Prasajak, Maryanne W. N. Kwok, Lady S. H. Ng, Lindy M. T. Wong, Lenina S. L. Poon, Mary K. L. Lai, Holly H. S. Cheng, S. K. Fong, Cindy F. Y. Leung, Jumpei Hasegawa, Hiroki Shirakawa, Sachiko Wakai, Makiko Mieno, Shuji Hatakeyama, Mariko Tateishi, Mutsuko Mihashi, Yuka Sato, Chalermpong Saenjum, Manu Deeudom, Prasit Tharavichitkul, Teerapat Ouirungrog, Phisit Ouirungroj, Terrence Chinniah, Jackson Tan, Kavitha Prabu, Sartaj Alam, Aung Kyaw Wynn, Rashidah Ahmad, Amalina Sidek, Dg Azizah Samsuddin, Noraini Ajis, Aliyah Ahmad, Susylawathi Magon, Boon Chu, Jiqiu Kuang, Yan Gao, Shoujun Wang, Yunxiao Hao, Rong Liu, Dongmei Li, Hui Wang, Ng Po Yan, Hisanori Nishio, Hitomi Mori, Yoshiko Morokuma, Takaaki Yamada, Makiko Kiyosuke, Sachie Yasunaga, Kazuhiro Toyoda, Nobuyuki Shimono, Dmitriy Babenko, Anar Turmuhambetova, Antonella Cheşcă, Mark A. Toleman, Dmitriy Babenko, Anar Turmuhambetova, Antonella Cheşcă, Mark A. Toleman, Dmitriy Babenko, Anar Turmuhambetova, Ilya Azizov, Antonella Cheşcă, Mark A. Toleman, Lyudmila L. Akhmaltdinova, Anar Turmuhambetova, Antonella Cheşcă, Dmitriy Babenko, Mark Albert Magsakay, Angelo Macatibag, Maria Fe Tayzon, Jeannica Kriselle Lerios, Ilya Azizov, Alyona Lavrineko, Dmitry Babenko, Eugene Sheck, Mikhail Edelstein, Tzu-Yin Liu, Lih-Yue Li, Chiung-Wen Chan, Hui-Chuan Pan, Tun-chieh Chen, Wipa Vanishakije, Warisra Jaikampun, Pei-Chen Cheng, Huey-Jen Huang, Shu-Ju Huang, Yu-Huan Huang, Su-Yin Li, Su-Fang Yu, Jian-Feng Li, Yu-Ping Wu, Yuan-Ti Lee, Chiao-Hui Lin, Ping-Chin Chang, Samatanet Tariyo, Sangwan Paengta, Ratchanee Wongsaen, Suttsiphan Thanompan, Sumawadee Skuntaniyom, Kumthorn Malathum, Suchada Sukkra, Khalequ Zaman, Sheikh Farzana Zaman, Farzana Zaman, Asma Aziz, Sayeed-Bin Faisal, Magali Traskine, Javier Ruiz-Guiñazú, Dorota Borys, Khalequ Zaman, Sheikh Farzana Zaman, Farzana Zaman, Asma Aziz, Sayeed-Bin Faisal, Magali Traskine, Javier Ruiz-Guiñazú, Dorota Borys, Wendy Wai Yee Lam, May Chow, Lucy Choy, Joseph Kam, Sharifah Azura Salleh, Razila Yacob, Siti Rokiah Yusof, Nordiah Awang Jalil, Jose Paulo Flor, Nicolo Andrei Añonuevo, Marko Bautista, V. Jay De Roxas, Justine Vergara, Maria Lourdes Millan, Marion Kwek, Jose Lito Acuin, Aisa Jensen Lee, Melecia A. Velmonte, Silverose Ann A. Bacolcol, Allan Alde, Keitleen Chavez, Arlene Joy Esteban, Ching-I Ting, Sunisa Dissayasriroj, Terrence Rohan Chinniah, Kavitha Prabu, Rashidah Ahmad, Susylawathi Magon, Jauharatud DiniSuhaimi, Aizzuddin Mirasin, Nurul Morni, Boon Chu, Azizah Samsuddin, Aliyah Ahmad, Amalina Sidek, Noraini Ajis, Amalina AbuBakar, Amanie Shafiee, Julaini Safar, Ng Po Yan, Leung Annie, Fung Yuk Ling, Lau Edna, Luk Kristine, Satoshi Shinomiya, Kumiko Yamamoto, Kayoko Kjiwara, Mitsuhiro Yamaguchi, Angela Chow, Grace Tin, Wei Zhang, Pei-Yun Hon, Bee-Fong Poh, Kalisvar Marimuthu, Brenda Ang, Ming-Chin Chan, Chih-Chien Wang, Shu-Ju Huang, Huey-Jen Huang, Su-Fang Yu, Huan-Yu Huang, Pei-Chen Cheng, Jian-Feng Li, Yuan-Ti Lee, Chiung-Ling Lai, Min-Chi Lu, Sajeerat Kosol, Wantana Sakolwirat, Patchanee Paepong, Sawalee Jansanga, Pattarin Jaisamoot, Nuttha Thongnuanual, Chittima Srithong, Somporn Somsakul, Kumthorn Malathum, Sutima Plongpunth, Mukkapon Punpop, Porntip Malathum, Kumthorn Malathum, Sutthiphan Thanomphan, Ratchanee Wongsaen, Kulada Peautiwat, Nattawipa boon kirdram, Wilawan Picheansathian, Pimpaporn Klunklin, Geetha Samethadka, Naoko Suzuki, Hitomi Asada, Masao Katayama, Atsushi Komano, Akihiro Sato, Itaru Nakamura, Hidehiro Watanabe, Tetsuya Matsumoto, Hye Kyung Seo, Joo-Hee Hwang, Myoung Jin Shin, Su Young Kim, Eu Suk Kim, Kyoung-Ho Song, Hong Bin Kim, Lai-Si Un, Choi-Ian Vong, Jose Paulo Flor, Nicolo Andrei Añonuevo, Marko Bautista, V. James De Roxas, Justine Vergara, Marion Kwek, Jocelyn Koh, Sherly Agustinus, Rozita Bte Abu Hassan, Yin Phyu Thinn, Benjamin Ng, Soe Pyae Tun, Su Mon Thi Ha, Xue Xiaoting, Lin Li, Leyland Chuang, Attanayaka Mudiyanselage Chulani Niroshika, Kaluarachchige Anoma Kaluarachchi Perera, Dimingo Kankanamalage Diana Grace Fernando, Bodhipakshage Rohini Hemamala, Chiu-yin Yeh, Huwi-chun Chao, Hui-Chun Yang, Hsiang-Ju Chiu, Ya-Ling Shih, Yu-Shan Chien, Wan-Yi Lin, Chia-Yun Pan, Ying-Yun Chang, Chiu-Yuch Yea, Ming-Hsien Chu, Li-Chu Lee, Hsiang-Ju Chiu, Ya-Ling Shih, Hui-Chun Yang, Lin Yu-Hsiu, Guo Siao-Pei, Leung Pak-On, Sie Mei-Fe, Chen Jyh-Jou, Lin Yu-Hsiu, Chang Yong-Yuan, Shu-Yuan Kuo, Yu-Hsiu Lin, Ji-Sheng Zhang, Pak-On Leung, Mei-Fe Sie, Jyh-Jou Chen, Yan-Ru Chen, Yu-Hsiu Lin, Ying-Ling Chen, Chi-Fen Taou, Hsiao-Shan Chen, Hung-Jen Tang, Shin Yu Chen, Yin Yin Chen, Fu Der Wang, Tzu-Ping Shih, Chin-Yu Chen, Su-Jung Chen, Mei-chi Wu, Wan-ju Yang, Mei-ling Chou, Man-Ling Yu, Li-Chu Li, Cheng-Wei Chu, Wen-Hao Tsou, Wen-Chih Wu, Wen-Chi Cheng, Cho-Ching Sun, Tzu-Ping Shih, Chin-Yu Chen, Shu-Hua Lu, Su-Jung Chen, Hsin-Ling Yang, Cheng-Yu Lu, Man-Ling Yu, Li-Chu Li, Cheng-Wei Chu, Wen-Hao Tsou, Wen-Chih Wu, Wen-Chi Cheng, Cho-Ching Sun, Nitchawan Hirunprapakorn, Kumthorn Malathum, Sirilux Apivanich, Ttipakorn Pornmee, Chonnikarnt Beowsomboon, Songyos Rajborirug, Yada Pruekrattananapa, Tharntip Sangsuwan, Silom Jamulitrat, Itthaporn Kumkoom, Nongyao Kasatpibal, Jittaporn Chitreecheur, Nongyao Kasatpibal, JoAnne D. Whitney, Surasak Saokaew, Kirati Kengkla, Margaret M. Heitkemper, Anucha Apisarnthanarak, Thanomvong Muntajit, Siriluk Apivanich, Kumthorn Malathum, Somporn Somsakul, Hang Thi Phan, Anh Pham Phuong Dinh, Tuyet Thi Kim Nguyen

**Affiliations:** 1 Deakin University, Melbourne, Australia; 2 Epworth HealthCare, Deakin University, Melbourne, Australia; 3 Eastern Health, Deakin University, Melbourne, Australia; 4 Austin Health, Deakin University, Melbourne, Australia; 5 Faculty of Medicine, Ramathibodi Hospital, Mahidol University, Bangkok, Thailand; 6 Microbiology Department, Faculty of Medicine, Pelita Harapan University, Tangerang, Banten Indonesia; 7 Neuro Department, Faculty of Medicine, Pelita Harapan University, Tangerang, Banten Indonesia; 8 Department of Infection Control and Prevention, Graduate School of Medicine, Osaka University, Osaka, Japan; 9 Division of Infection Control and Prevention, Osaka University Hospital, Osaka, Japan; 10 Research Institute for Microbial Diseases, Osaka University, Osaka, Japan; 11 Tohoku Bio-Array, Sendai, Japan; 12 Faculty of Medicine Ramathibodi Hospital, Mahidol University, Bangkok, Thailand; 13 Division of Infectious Diseases, Department of Pediatrics, Tokyo Metropolitan Children’s Medical Center, Fuchu, Tokyo, Japan; 14 Department of Pediatrics, Nagasaki University Hospital, Nagasaki, Japan; 15 Department of Neonatal Medicine, Osaka Medical Center and Research Institute for Maternal and Child Health, Osaka, Japan; 16 Karaganda State Medical University, Karaganda, Kazakhstan; 17 Institute of Antimicrobial Chemotherapy, Smolensk, Russia; 18 Department of Laboratory Medicine, Kosin University College of Medicine, Busan, Korea; 19 Department of Dental Hygiene, Silla University, Busan, Korea; 20 Yeongam-gun Public Health Center, Yeongam, Korea; 21 Department of Laboratory Medicine and Research Institute of Bacterial Resistance, Yonsei University College of Medicine, Seoul, Korea; 22 School of Pharmacy, Taylor’s University Lakeside Campus, Subang Jaya, Selangor Darul Ehsan Malaysia; 23 School of Medicine, Taylor’s University, Subang Jaya, Selangor Malaysia; 24 Department of Medical Microbiology and Parasitology, Faculty of Medicine and Health Sciences, University Putra Malaysia, Selangor, Malaysia; 25 Department of Biomedical Sciences, Faculty of Medicine and Health Sciences, University Putra Malaysia, Selangor Darul Ehsan, Malaysia; 26 Department of Microbiology, Faculty of Medicine, University Malaya Medical Centre, Kuala Lumpur, Malaysia; 27 Hospital Infection Control and Epidemiology Center, The Medical City, Marilao, Bulacan, Philippines; 28 Tan Tock Seng Hospital, Singapore, Singapore; 29 Tan Tock Seng Hospital, Singapore, Singapore; 30 Medical and Molecular Microbiology Unit, Department of Medicine, University of Fribourg, Fribourg, Switzerland; 31 Hospital UniversitarioVirgen del Rocio y Virgen Macarena, Seville, Spain; 32 Escola Superior de Saude da Cruz Vermelha Portuguesa, Lisbon, Portugal; 33 University of Lausanne and University Hospital Center, Lausanne, Switzerland; 34 Prince of Songkla University, Hat Yai, Songkla Thailand; 35 Madras Medical Mission, Chennai, Tamil Nadu India; 36 Department of Microbiology, Faculty of Medicine, University of Pelita Harapan, Tangerang, Banten Indonesia; 37 Department of Pharmacy, Siloam Teaching Hospitals, Lippo Village, Tangerang, Indonesia; 38 Department of Internal Medicine, Faculty of Medicine, University of Pelita Harapan, Tangerang, Banten Indonesia; 39 Toho University Ohashi medical center, Tokyo, Japan; 40 Manila Doctors Hospital, Manila, Philippines; 41 Emergency Medicine, Department of Emergency and Critical Care Medicine, Wan Fang Medical Center, Taipei Medical University, Taipei, Taiwan; 42 Department of emergency and critical care medicine, School of Medicine, College of Medicine, Taipei Medical University, Taipei, Taiwan; 43 Division of Infectious Diseases, Department of Internal Medicine, Shin Kong Wu Ho-Su Memorial Hospital, Taipei, Taiwan; 46 Infection Control Team, Chung Shan Medical University Hospital, Taichung, Taiwan; 47 Infectious Diseases Division, Chung Shan Medical University Hospital, Taichung, Taiwan; 48 Infectious Diseases Division, China Medical University Hospital, Taichung, Taiwan; 49 Division of Infectious Diseases, Faculty of Medicine, Thammasat University, Bangkok, Thailand; 50 Chulabhorn International College of Medicine, Thammasat University, Bangkok, Thailand; 51 Bryn Mawr Hospital, MainLine Health Care, Bryn Mawr, Pennsylvania USA; 52 Quality management division in Hung Vuong hospital, Ho Chi Minh, Viet Nam; 53 Infection control department in Hung Vuong Hospital, Ho Chi Minh, Viet Nam; 54 National Children’s Hospital, Dong Da, Hanoi, Viet Nam; 55 Central Sterilizing Services Department, Manager Macquarie University Hospital, North Ryde, New South Wales Australia; 56 Apollo Hospitals, Chennai, Tamil Nadu India; 57 Apollo Hospitals, Karapakkam, Tamil Nadu India; 58 Shin Koga Hospital, Kurume, Fukuoka Japan; 59 Lovisenberg Diakonale Hospital, Oslo, Norway; 60 Oslo Univerisity Hospital, Oslo, Norway; 61 Central Supply Room, Department of Nursing, Mackay memorial hospital, Taipei, Taiwan; 62 Infection control center, Mackay memorial hospital, Taipei, Taiwan; 63 Central Supply Room, Department of Nursing, Mackay memorial hospital, Taipei, Taiwan; 64 Infection control center, Mackay memorial hospital, Taipei, Taiwan; 65 Wanonniwas Hospital, Wanonniwas, Sakonnakhon, Thailand; 66 CSSD, Maharaj Nakorn Chiang Mai Hospital, Faculty of Medicine, Chiang Mai University, Chiang Mai, Thailand; 67 Pose Health Care Co., Ltd., Kannayao, Bangkok, Thailand; 68 Central sterile services department (CSSD), Maharaj Nakorn Chiang Mai Hospital, Faculty of Medicine, Chiang Mai University, Chiang Mai, Thailand; 69 Department of Pharmaceutical Sciences, Faculty of Pharmacy, Chiang Mai University, Chiang Mai, Thailand; 70 Pose Health Care Co., Ltd., Kannayao, Bangkok, Thailand; 71 Infection Prevention and Hospital Epidemiology Unit, Department of Infectious Diseases, Alfred Health, Melbourne, Australia; 72 Gai-huan Henan Provincial Infectious Disease Hospital, Zhengzhou, China; 73 Queen Elizabeth Hospital, Hong Kong, China; 74 Squina International Centre for Infection Control, School of Nursing, The Hong Kong Polytechnic University (PolyU), Hong Kong, China; 75 Department of Health Technology and Informatics, The Hong Kong Polytechnic University (PolyU), Hong Kong, China; 76 Department of Building Services Engineering, The Hong Kong Polytechnic University (PolyU), Hong Kong, China; 77 Queen Elizabeth Hospital, Hong Kong, China; 78 Kurume University, Kurume, Fukuoka Japan; 79 Asian Hospital and Medical Center, Muntinlupa, National Capital Region Philippines; 80 National kidney foundation, Singapore, Singapore; 81 Jurong West1 Dialysis Centre, Singapore, Singapore; 82 Kolam Ayer Dialysis Centre, Singapore, Singapore; 83 Woodlands1 Dialysis Centre, Singapore, Singapore; 84 Teck Whye Dialysis Centre, Singapore, Singapore; 85 Yishun1 Dialysis Centre, Singapore, Singapore; 86 Hougang1 Dialysis Centre, Singapore, Singapore; 87 Kim Keat Dialysis Centre, Singapore, Singapore; 88 Tampines2 Dialysis Centre, Singapore, Singapore; 89 Infection Control Unit, Tan Tock Seng Hospital, Singapore, Singapore; 90 Infection Control Unit, Tan Tock Seng Hospital, Singapore, Singapore; 91 Infectious Disease, Tan Tock Seng Hospital, Singapore, Singapore; 92 Clinical Epidemiology, Tan Tock Seng Hospital, Singapore, Singapore; 93 Infectious Disease, Tan Tock Seng Hospital, Singapore, Singapore; 94 Lo-Tung Pohai Hospital, Lo-Hsu Foundation, Incl, Ilan, Luodong Town, Taiwan; 95 Infection control center, Mackay memorial hospital, Hsinchu, Taiwan; 100 Committee of Infection Control, Chang Gung University Medical College, Kaohsiung, Taiwan; 101 Departments of Clinical Pathology, Chang Gung University Medical College, Kaohsiung, Taiwan; 102 Division of Infectious Diseases, Chang Gung University Medical College, Kaohsiung, Taiwan; 103 Internal Medicine, Kaohsiung Chang Gung Memorial Hospital, Kaohsiung, Taiwan; 104 E-DA Hospital, Kaohsiung, Taiwan; 105 Clinical Governance Unit, International Centre for Diarrhoeal Disease Research, Bangladesh (icddr, b), Dhaka, Bangladesh; 106 The Hong Kong Polytechnic University, North Point, Hong Kong, China; 107 School of Medical and Health Sciences Tung Wah College, Hong Kong, China; 108 Dietetic Department, Matilda International Hospital, Hong Kong, China; 109 Nursing Department, Matilda International Hospital, Hong Kong, China; 110 Infection Control Team, Caritas Medical Centre, Hospital Authority, Hong Kong, China; 111 Hospital Authority, Hong Kong, China; 112 Baptist Hospital, Hong Kong, China; 113 HKICNA, Hong Kong, China; 114 HK Sanatorium & Hospital, Hong Kong, China; 115 Matilda International Hospital, Hong Kong, China; 116 Shiraz University of Medical Sciences, Shiraz, Iran; 117 Indiana University School of Dentistry, Indianapolis, IN USA; 118 Shiraz University of Medical Sciences, Shiraz, Iran; 119 Indiana University School of Dentistry, Indianapolis, IN USA; 120 Department of Infection Prevention and Control, Tokyo Medical University Hospital, Tokyo, Japan; 121 Department of Microbiology, Tokyo Medical University, Tokyo, Japan; 122 Asian Hospital and Medical Center, Muntinlupa, National Capital Region Philippines; 123 Asian Hospital and Medical Center, Muntinlupa, National Capital Region Philippines; 124 Tan Tock Seng Hospital, Singapore, Singapore; 125 Kaohsiung Municipal Ta-Tung Hospital, Kaohsiung, Cianjin District Taiwan; 126 Department of Nursing, Chi Mei Medical Center, Liouying, Tainan, Taiwan; 127 Infection control office, Longtan Min-Sheng hospital, Longtan, Taoyuan Taiwan; 128 Ramathibodi Hospital, Bangkok, Thailand; 129 Infection Control and Prevention Unit, Nakornping Hospital, ChaingMai, Thailand; 130 Nakornping Hospital, Chiang Mai, Thailand; 131 Department of Pediatrics, Faculty of Medicine, Chulalongkorn University, Bangkok, Thailand; 132 Research Unit in Pediatric Infectious Diseases and Vaccines, Faculty of Medicine, Chulalongkorn University, Bangkok, Thailand; 133 Division of Critical Critical Care, Faculty of Medicine, Chulalongkorn University, Bangkok, Thailand; 134 Division of Hematology-Oncology, Faculty of Medicine, Chulalongkorn University, Bangkok, Thailand; 135 Department of Pediatrics, King Chulalongkorn Memorial Hospital, Bangkok, Thailand; 136 Chao Phya Abhaibhubejhr Hospital, Prachinburi, Thailand; 137 Faculty of Nursing, Chiang Mai University, Chiang Mai, Thailand; 138 GOJO Industries, Inc., Akron, OH USA; 139 HygCen International GmbH, Schwerin, Germany; 140 Dr. Brill + Partner GmbH, Hamburg, Germany; 141 Hospital Albert Einstein, Sao Paulo, Brazil; 142 GOJO Industries, Inc, Akron, Ohio USA; 143 Bioscience laboratories, Inc, Bozeman, Montana USA; 144 Quality Management Division, Hung Vuong Hospital, Ho Chi Minh City, Vietnam; 145 Infection Control Department, Hung Vuong Hospital, Ho Chi Minh City, Vietnam; 146 University of Sydney, New South Wales Sydney, Australia; 147 Madras Medical Mission, Chennai, Tamil Nadu India; 148 SRM Institutes for Medical Sciende (SIMS) Hospital, Chennai, India; 149 Kasturba Medical College Manipal, Karnataka, India; 150 100 Degree Fahrenheit (F), Chennai, India; 151 Department of Pharmacy, Aomori Prefectural Central Hospital, Aomori, Japan; 152 Department of Laboratory Medicine, Aomori Prefectural Central Hospital, Aomori, Japan; 153 Department of Pharmacy, Aomori Prefectural Central Hospital, Aomori, Japan; 154 Department of Laboratory Medicine, Aomori Prefectural Central Hospital, Aomori, Japan; 155 Department of Clinical Laboratory Medicine, Hirosaki University Graduate School of Medicine, Aomori, Japan; 156 Division of Infection Control and Prevention, Kurume University Hospital, Kurume, Japan; 157 Infection Prevention and Control Office, Manila Doctors Hospital, Manila, Philippines; 158 Tan Tock Seng Hospital, Singapore, Singapore; 159 Tan Tock Seng Hospital, Singapore, Singapore; 160 Tan Tock Seng Hospital, Singapore, Singapore; 161 Chi Mei Medical Center, Intensive Care Medicine, Yongkang, Taiwan; 162 Intensive care unit, Chimei medical center, Tainan, Taiwan; 163 Infection Control Nurse, Suratthani Hospital, Suratthani, Thailand; 164 Prince of Songkla University, Hat Yai, Songkla, Thailand; 165 Infection Control and Prevention Unit, Nakornping Hospital, ChaingMai, Thailand; 166 Department of Laboratory, Nakornping Hospital, ChaingMai, Thailand; 167 National Children’s Hospital, Dong Da, Hanoi, Viet Nam; 168 Karaganda State Medical University, Karaganda, Kazakhstan; 169 Institute of Antimicrobial Chemotherapy, Smolensk, Russia; 170 Karaganda State Medical University, Karaganda, Kazakhstan; 171 Institute of Antimicrobial Chemotherapy, Smolensk, Russia; 172 Armed Forces Daejeon Hospital, Daejeon, Korea; 173 Seoul National University Bundang Hospital, Seoul, Korea; 174 Seoul National University Hospital, Seoul, Korea; 175 Department of Microbiology, Faculty of Life Sciences, University of Benin, Benin, Edo Nigeria; 176 Show Chwan Memorial Hospital, Changhua, Taiwan; 177 Departments of Medical Laboratory, Show Chwan Memorial Hospital, Changhua, Taiwan; 178 Infection Control Office, Show Chwan Memorial Hospital, Changhua, Taiwan; 179 Department of infection, Mackay memorial hospital, Taipei, Taiwan; 180 Infection control center, Mackay memorial hospital, Taipei, Taiwan; 181 Laboratory Medicine Department, National Taiwan University Hospital Hsin-Chu Branch, Hsin-chu, Taiwan; 182 Department of Emergency Medicine, National Taiwan University Hospital Hsin-Chu Branch, Hsin-chu, Taiwan; 183 Division of Infectious Diseases, National Taiwan University Hospital Hsin-Chu Branch, Hsin-chu, Taiwan; 184 Department of Pharmaceutical Sciences, Faculty of Pharmacy, Chiang Mai University, Chiang Mai, Thailand; 185 PoseHealthCare Co., Ltd., Kannayao, Bangkok, Thailand; 186 Faculty of Medical Technology, Rangsit University, Pathumthani, Thailand; 187 Department of Medicine, Queen Elizabeth Hospital, Hong Kong, China; 188 Department of Nephrology, Tokyo Metropolitan Health and Medical Treatment Corporation Okubo Hospital, Kabukicho, Shinjuku-ku, Tokyo, Japan; 189 Department of Urology, Tokyo Women’s Medical University, Kawadacho, Shinjuku-ku, Tokyo, Japan; 190 Department of Urology, Tokyo Metropolitan Health and Medical Treatment Corporation Okubo Hospital, Kabukicho, Shinjuku-ku, Tokyo, Japan; 191 Department of Medical Informatics, Center for Information, Jichi Medical University, Yakushiji, Shimotsuke-shi, Tochigi Japan; 192 Division of General Internal Medicine/Division of Infectious Diseases, Jichi Medical University Hospital, Yakushiji, Shimotsuke-shi, Tochigi Japan; 193 Department of Internal Medicine, Tokyo Metropolitan Health and Medical Treatment Corporation Okubo Hospital, Kabukicho, Shinjuku-ku, Tokyo, Japan; 194 Kurume University School of Nursing, Kurme, Fukuoka, Japan; 195 Department of Pharmaceutical Sciences, Faculty of Pharmacy, Chiang Mai University, Chiang Mai, Thailand; 196 Department of Microbioloigy, Faculty of Medicine, Chiang Mai University, Chiang Mai, Thailand; 197 Pose Health Care Co., Ltd., Kannayao, Bangkok, Thailand; 198 Raja Isteri Pengiran Anak Saleha Hospital, Bandar Seri Begawan, Brunei Muara Brunei Darussalam; 199 Infection Control and Prevention Department, Peking University People’s Hospital, Beijing, China; 200 Clinical Laboratory, Peking University People’s Hospital, Beijing, China; 201 Caritas Medical Centre, Hospital Authority, Hong Kong, China; 202 Kyushu University Hospital, Fukuoka, Japan; 203 Karaganda State Medical University, Karaganda, Kazakhstan; 204 Transilvania University of Braşov, Braşov, Romania; 205 Cardiff University, Cardiff, Wales UK; 206 Karaganda State Medical University, Karaganda, Kazakhstan; 207 Transilvania University of Braşov, Braşov, Romania; 208 Cardiff University, Cardiff, Wales UK; 209 Karaganda State Medical University, Karaganda, Kazakhstan; 210 Institute of Antimicrobial Chemotherapy, Smolensk, Russia; 211 Transilvania University of Braşov, Braşov, Romania; 212 Cardiff University, Cardiff, Wales UK; 213 Karaganda State Medical University, Karaganda, Kazakhstan; 214 Transilvania University of Braşov, Braşov, Romania; 215 Hospital Infection Control and Epidemiology Center, The Medical City, Quezon, Metro Manila, Philippines; 216 Department of Cardiology, The Medical City, Quezon, Metro Manila, Philippines; 217 Institute of Antimicrobial Chemotherapy, Smolensk, Russian Federation; 218 Karagamnda State Medical University, Karagamnda, Kazakhstan; 219 Kaohsiung Municipal Ta-Tung Hospital, Kaohsiung, Cianjin Taiwan; 220 Somdetchaopraya institute of psychiatry, Klongsan, Bangkok, Thailand; 221 Infection Control Team, Chung Shan Medical University Hospital, Taichung, Taiwan; 222 Infectious Diseases Division, Chung Shan Medical University Hospital, Taichung, Taiwan; 223 Department of Nursing Medicine, Chung Shan Medical University Hospital, Taichung, Taiwan; 224 Chi Mei Medical Center, Liouying, Tainan, Taiwan; 225 Infection Control and Prevention Unit, Nakornping Hospital, ChaingMai, Thailand; 226 Ramathibodi Hospital, Bangkok, Thailand; 227 International Centre for Diarrheal Disease Research, Dhaka, Bangladesh; 228 GlaxoSmithKline (GSK) Vaccines, Wavre, Belgium; 229 International Centre for Diarrheal Disease Research, Dhaka, Bangladesh; 230 GSK Vaccines, Wavre, Belgium; 231 Infection Control Unit, Canossa Hospital (Caritas), Hong Kong, China; 232 Canossa Hospital (Caritas), Hong Kong, China; 233 Department of Medical Microbiology and Immunology, Universiti Kebangsaan Malaysia Medical Centre, Cheras, Kuala Lumpur, Malaysia; 234 Infection Control Unit, Universiti Kebangsaan Malaysia Medical Centre, Cheras, Kuala Lumpur, Malaysia; 235 Asian Hospital and Medical Center, Muntinlupa, National Capital Region Philippines; 236 Infection Prevention and Control Office, Manila Doctors Hospital, Manila, National Capital Region Philippines; 237 Chi-mei medical center intensive care unit, Tainan, Taiwan; 238 Medical Wings, Siam Land Flying, Bangkok, Thailand; 239 Raja Isteri Pengiran Anak Saleha Hospital, Bandar Seri Begawan, Brunei Muara Brunei Darussalam; 240 Infection Control Team, Caritas Medical Centre, Hospital Authority, Hong Kong, China; 241 Minoh City Hospital, Minoh, Japan; 245 Tan Tock Seng Hospital, Singapore, Singapore; 246 Infection Control Office, Tri-Service General Hospital, National Defense Medical Center, Taipei, Taiwan; 247 Department of Pediatrics, Tri-Service General Hospital, National Defense Medical Center, Taipei, Taiwan; 248 Infection Control Team, Chung Shan Medical University Hospital, Taichung, Taiwan; 249 Infectious Diseases Division, Chung Shan Medical University Hospital, Taichung, Taiwan; 250 Department of Nursing Medicine, Chung Shan Medical University Hospital, Taichung, Taiwan; 251 Infectious Diseases Division, China Medical University Hospital, Taichung, Taiwan; 252 Suratthani Hospital, Suratthani, Thailand; 253 Ramathibodi Hospital, Bangkok, Thailand; 254 Faculty of Medicine Ramathibodi Hospital, Bangkok, Thailand; 255 Nakornping Hospital, Chiang Mai, Thailand; 256 Faculty of Nursing, Chiang Mai University, Chiang Mai, Thailand; 257 Consultant microbiologist and infection control officer, Bhagwan Mahaveer Jain Hospital, Bangalore, Karnataka India; 258 National Hospital Organization Nagoya Medical Center, Nagoya, Aichi Japan; 259 Tokyo Medical University Hospital, Shinjyuku-ku, Tokyo, Japan; 260 Department of Internal Medicine, Seoul National University Bundang Hospital, Seongnam, Republic of Korea; 261 Infection Control Office, Seoul National University Bundang Hospital, Seongnam, Republic of Korea; 262 Health Bureau of Macau SARS, Macau, Macau; 263 Asian Hospital and Medical Center, Muntinlupa, National Capital Region Philippines; 264 Department of Medical Services, Ang Mo Kio-Thye Hua Kwan Hospital, Singapore, Singapore; 265 Department of Nursing Services, Ang Mo Kio-Thye Hua Kwan Hospital, Singapore, Singapore; 266 Department of Allied Health, Ang Mo Kio-Thye Hua Kwan Hospital, Singapore, Singapore; 267 Department of Health Performance Office, Ang Mo Kio-Thye Hua Kwan Hospital, Singapore, Singapore; 268 Department of Medicine, Ng Teng Fong General Hospital, Singapore, Singapore; 269 Department of Nursing Training & Development, Ng Teng Fong General Hospital, Singapore, Singapore; 270 National Hospital of Sri Lanka, Colombo, Sri Lanka; 271 Chimei medical center, Tainan, Taiwan; 272 Department of Nursing, En Chu Kong Hospital, Sanxia, New Taipei City, Taiwan; 273 Taipei Veterans General Hospital, Yilan, Taiwan; 274 Department of Nursing, En Chu Kong Hospital, New Taipei City, Taiwan; 275 Chi Mei Medical Center, Liouying, Tainan, Taiwan; 276 Chi Mei Medical Center, Liouying, Tainan, Taiwan; 277 Kaohsiung Medical University, Kaohsiung, Taiwan; 278 Chi Mei Medical Center, Liouying, Tainan, Taiwan; 279 Chi Mei Medical Center, Liouying, Tainan, Taiwan; 280 Infection Control Committee, Chi Mei Medical Center, Tainan, Taiwan; 281 Infection control, Taipei Veterans General Hospital, Taipei, Taiwan; 282 Taipei Veterans General Hospital Yuanshan Branch, Taipei, Taiwan; 283 Institute of Public Health and School of Medicine, National Yang-Ming University, Taipei, Taiwan; 284 Mary’s Junior College Of Medicing And Management, Yilan, Taiwan; 285 Infection Control Committee, Taipei Veterans General Hospital Yuanshan Branch, Taipei, Taiwan; 286 Department of Nursing, Taipei Veterans General Hospital Yuanshan Branch, Taipei, Taiwan; 287 Department of Medicine, Taipei Veterans General Hospital Yuanshan Branch, Taipei, Taiwan; 288 Department of Surgery, Taipei Veterans General Hospital Yuanshan Branch, Taipei, Taiwan; 289 Vice Superintendent, Taipei Veterans General Hospital Yuanshan Branch, Taipei, Taiwan; 290 Superintendent, Taipei Veterans General Hospital Yuanshan Branch, Taipei, Taiwan; 291 Institute of Public Health and School of Medicine, National Yang-Ming University, Taipei, Taiwan; 292 Section of Infectious Diseases, Department of Medicine, Taipei Veterans General Hospital, Taipei, Taiwan; 293 Department of Teaching units, ST. Mary’s Junior College Of Medicing And Management, Yilan, Taiwan; 294 Ramathibodi Hospital, Bangkok, Thailand; 295 Department of Community Medicine, Faculty of Medicine, Prince of Songkla University, Songkla, Thailand; 296 Registered Nurse, Saint Louis Hospital, Bangkok, Thailand; 297 Associate Professor, Faculty of Nursing, Chiang Mai University, Chiang Mai, Thailand; 298 Division of Nursing Science, Faculty of Nursing, Chiang Mai University, Chiang Mai, Thailand; 299 Department of Biobehavioral Nursing and Health Systems, School of Nursing, University of Washington, Seattle, WA USA; 300 Center of Health Outcomes Research and Therapeutic Safety (Cohorts), School of Pharmaceutical Sciences, University of Phayao, Phayao, Thailand; 301 School of Pharmacy, Monash University Malaysia, Selangor Darul Ehsan, Malaysia; 302 Division of Infectious Diseases, Thammasart University Hospital, Pratumthani, Thailand; 303 Ramathibodi Hospital, Bangkok, Thailand; 304 Quality management division in Hung Vuong hospital, Ho Chi Minh city, Viet Nam; 305 Infection control department in Hung Vuong Hospital, Ho Chi Minh city, Viet Nam

## Session: Antimicrobial Resistance

### AR1 Key stakeholders’ perspectives on the underlying causes of antimicrobial resistance in Thailand

#### Nantanit Sutthiruk^1^, Mari Botti^2^, Julie Considine^3^, Andrea Driscoll^4^, Ana Hutchinson^2^, Kumthorn Malathum^5^

##### ^1^Deakin University, Melbourne, Australia; ^2^Epworth HealthCare, Deakin University, Melbourne, Australia; ^3^Eastern Health, Deakin University, Melbourne, Australia; ^4^Austin Health, Deakin University, Melbourne, Australia; ^5^Faculty of Medicine, Ramathibodi Hospital, Mahidol University, Bangkok, Thailand

###### **Correspondence:** Nantanit Sutthiruk (nsutthir@deakin.edu.au)

**Background**

Antimicrobial resistance (AMR) is a major problem worldwide. Antimicrobial stewardship (AMS) has the vital aim of ensuring optimal use of antimicrobial medicines to minimize AMR. New strategies are needed to reduce AMR. It is vital to ensure that key stakeholders are involved in the development of these strategies. This study aimed to examine key stakeholders’ perspectives on the underlying causes of AMR in Thailand.

**Materials and methods**

Semi-structured interviews were conducted with 15 key multidisciplinary clinicians, heads of department and healthcare administrators who were involved in AMS programs in a 1,000-bed university hospital in Bangkok Thailand. Qualitative content analysis was used to analyze the interview data.

**Results**

One of the key themes that emerged was lack of regulatory control resulting in widespread antibiotic availability and use both in health and agriculture in Thailand, including over-the-counter availability of antibiotics. This ease of accessibility combined with poor consumer knowledge was considered one of the most important contributors to the increasing prevalence of AMR. The development and implementation of more effective infection prevention and control strategies was identified as a priority, particularly in healthcare. Three major concerns related to the perception that many patients admitted to hospital already have AMR infections, that staff prescribing behaviors are not ideal, and that the lack of resources to develop and implement AMS programs is an important barrier to decreasing the overuse of antibiotics.

**Conclusions**

Participants recognized that AMR is a major problem in Thailand and in healthcare. There was agreement that what is required is better regulatory control of antibiotics and medical engagement in AMS.

## Session: Antimicrobial Resistance

### AR2 Bacteriological profile of neurosurgical infections in a secondary care centre, Tangerang, Indonesia: implication for empirical antibiotic treatment

#### Cucunawangsih Cucunawangsih^1^, Veronica Wiwing^1^, Vivien Puspitasari^2^

##### ^1^Microbiology Department, Faculty of Medicine, Pelita Harapan University, Tangerang, Banten, Indonesia; ^2^Neuro Department, Faculty of Medicine, Pelita Harapan University, Tangerang, Banten, Indonesia

###### **Correspondence:** Cucunawangsih Cucunawangsih (cucunawangsih.fk@uph.edu)

**Background**

Antimicrobial resistance is a major problem of post-operative neurosurgical infection over the recent years. This study aimed to evaluate an increasing trend of infection in neurosurgical patients and susceptibility pattern of the causative pathogen.

**Material and methods**

Over a period of five years (June 2010 to June 2015), 216 cerebrospinal fluid and pus samples derived from clinically suspected cases of post-operative neurosurgical infection were processed using the standard procedures for culture and antibiotic susceptibility testing.

**Results**

Of these 216 patients, causative pathogens were identified in 55 patients (25.5%). Majority of infections were caused by multidrug- resistant gram-negative bacilli (MDRGNB) including *Pseudomonas aeruginosa* (n = 7, 12.7%), *Acinetobacter baumannii* (n = 6, 10.9%), *Sphingomonas paucimobilis* (n = 5, 9.0%), *Escherichia coli*(n = 4, 7.3%), *Aeromonas salmonocida* (n = 3, 5.4%), and *Klebsiella pneumoniae (n =* 3, 5.4%). The common isolates showed a high susceptibility to tigecycline (86.7%) and amikacin (90%), ceftriaxone (76.9%) and ceftazidime (70%). All Gram-positive bacteria isolates were susceptible to tigecycline and vancomycin.

**Conclusions**

Based on our result of susceptibility pattern, a combination of tigecycline and amikacin should be considered for empirical therapy to treat MDRGNB infections. This finding pointed that strict antibiotic policies were required to work out the issue of emerging MDR-GNB infections.

## Session: Antimicrobial Resistance

### AR3 A newly developed single-strand tag hybridization – printed array strip technique for identification of carbapenemase-producing Enterobacteriaceae

#### Rathina Kumar Shanmugakani^1,2,3^, Yukihiro Akeda^1,2,3^, Takuya Kodera^4^, PitakSantanirand^5^, Kazunori Tomono^1,2^

##### ^1^Department of Infection Control and Prevention, Graduate School of Medicine, Osaka University, Osaka, Japan; ^2^Division of Infection Control and Prevention, Osaka University Hospital, Osaka, Japan; ^3^Research Institute for Microbial Diseases, Osaka University, Osaka, Japan; ^4^Tohoku Bio-Array, Sendai, Japan; ^5^Faculty of Medicine Ramathibodi Hospital, Mahidol University, Bangkok, Thailand

###### **Correspondence:** Rathina Kumar Shanmugakani (rathinabiotech@gmail.com)

**Background**

Carbapenem resistant organisms are known to risk the life of both immunocompromised and immunocompetent patients due to their resistance towards the drug of the last resort. In carbapenemase-producing Enterobacteriaceae (CPE), carbapenemase genes serve as the main reservoir of carbapenem resistance due to their plasmid-mediated transferability to naïve Enterobacteriaceae. In this regard, CPE infections are a global health issue due to their hasty dissemination throughout the world. The detection of CPE at the earliest is crucial to control its transmission. Several detection systems are being developed and modified for CPE detection but have various constraints in different criteria.

**Materials and methods**

We attempted to develop single-strand tag hybridization –printed array strip (STH-PAS), a new genotypic multiplex detection system to detect CPE directly in clinical samples. STH-PAS is a PCR-based technique that targets the four major carbapenemase genes - *bla*_NDM_, *bla*_KPC_, *bla*_IMP_, and *bla*_OXA-48_ for CPE detection. The sensitivity and specificity of STH-PAS in detecting the CPE were determined for the clinical isolates and direct clinical specimens.

**Results**

STH-PAS showed 100% sensitivity and specificity in detecting the CPE clinical isolates in comparison with the culture methods and PCR. For detection of CPE directly in stool specimens, STH-PAS showed a sensitivity and specificity of 92% and 99.4%, respectively.

**Conclusions**

The results of the current study depict that STH-PAS possesses several advantages as a good detection system for CPE. The simplicity and rapidity of STH-PAS show that it could serve as an effective tool for surveillance and infection control purposes

## Session: Antimicrobial Resistance

### AR4 Invasive infections due to multidrug-resistant gram-negative bacilli among Japanese children

#### Takayuki Yamanaka^1^, Hiroyuki Moriuchi^2^, Hiroyuki Kitajima^3^, Yuho Horikoshi^1^

##### ^1^Division of Infectious Diseases, Department of Pediatrics, Tokyo Metropolitan Children's Medical Center, Fuchu, Tokyo, Japan; ^2^Department of Pediatrics, Nagasaki University Hospital, Nagasaki, Japan; ^3^Department of Neonatal Medicine, Osaka Medical Center and Research Institute for Maternal and Child Health, Osaka, Japan

###### **Correspondence:** Takayuki Yamanaka (t.yamanaka122@gmail.com)

**Background**

Although multidrug-resistant (MDR) gram-negative bacilli (GNB) become a global concern, the disease burden of MDR GNB in children has not been reported yet in Japan. We elucidate the impact of invasive MDR GNB infections among Japanese children in the hospital setting.

**Materials and methods**

A primary questionnaire was sent to 520 pediatric training facilities. A secondary questionnaire was sent to determine whether any cases showed a positive blood or cerebral spinal fluid culture for Extended Spectrum Beta-lactamase (ESBL) producing GNB, *Amp*C β-lactamases producing GNB, or carbapenem-resistant enterobacteriacae (CRE) between April 2012 and March 2015.The following data were collected; demographic data pertaining to both the care facilities and patients, clinical diagnosis, and outcomes.

**Results**

The response rate for the primary questionnaire was 57%. Among facilities that responded, 66 facilities were eligible for the secondary questionnaire. The response rate for secondary questionnaire was 48%. A total of 92 pediatric patients had invasive MDR GNB infection. The median age was 2.5 years old (interquartile range 3 months–10 years old). The number of patients with bacteremia caused by ESBL GNB, *Amp*C GNB, and CRE were 66 (72%), 22 (24%), and 4 (4%), respectively. The clinical diagnosis of ESBL and *Amp*C GNB showed 53 cases of sepsis. The clinical diagnosis of CRE showed 2 cases of catheter related blood stream infection and 2 cases of sepsis. Mortality at 30 days for ESBL, *Amp*C and CRE bacteremia was 6%, 9% and 0%, respectively.

**Conclusions**

The most common MDR GNB bacteremia was ESBL GNB among children in this survey.

## Session: Antimicrobial Resistance

### AR5 The role of non-specific microflora in patients with tuberculosis

#### Alyona Lavrinenko^1^, Ilya Azizov^2^, Nurlan Tabriz^1^, Margulan Kozhamuratov^1^, Yekatherine Serbo^1^

##### ^1^Karaganda State Medical University, Karaganda, Kazakhstan; ^2^Institute of Antimicrobial Chemotherapy, Smolensk, Russia

###### **Correspondence:** Alyona Lavrinenko (lavrinenko.alena@gmail.com)

**Background**

Tuberculosis is often complicated by the addition of non-specific inflammation, which changes not only the clinical manifestation of tuberculosis, but the course and outcome of disease. This study aimed to study the spectrum of non-specific microflora from patients with active tuberculosis and to evaluate its susceptibility to antimicrobial agents.

**Materials and methods**

The study was conducted in 2014-2015; 343 sputum samples were investigated. Identification of microorganisms was carried out by MALDI-TOF methods using mass-spectrometer Microflex (Bruker Daltonics, Germany). The sensitivity of microorganisms to antibiotics was determined by disk-diffusion methods (CLSI 2012). Statistical processing and data analysis was performed using WhoNet 6.3 program.

**Results**

The growth of non-specific microflora in patients with tuberculosis was obtained in 21% of cases. The predominant etiologic role in non-specific inflammation belonged to *S. aureus* (22%), *K. pneumoniae* were isolated in 12.5%, *A. baumannii* – in 11.1%. Remaining microorganisms were isolated in individual cases. 12.5% staphylococci were MRSA, to other anti-staphylococcal drugs *S. aureus* has kept a high sensitivity. Isolated *K. pneumoniae* strains were resistant to cephalosporins of the 3rd generation in 12.5%, the resistance to meropenem marked in 11.1%. *A. baumannii* was characterized by a high resistance to antibiotics – 85.7% ESBL-producing strains, 37.5% and 62.5% strains were resistant to imipenem and meropenem, 80% *A. baumannii* strains were resistant to fluoroquinolones.

**Conclusions**

According to antibioticogram data, the isolated microorganisms obtained from non-specific microflora of patients with tuberculosis, may adversely affect the course of the disease and impede the selection of antibacterial drugs and affect the outcome of the disease.

## Session: Antimicrobial Resistance

### AR6 Antimicrobial susceptibility of clinical isolates of *Enterococcus faecium* and *Enterococcus faecalis* in Korea

#### Dahae Yang^1^, Woonhyoung Lee^1^, Il Kwon Bae^2^, Jae Hyun Lee^3^, Hyukmin Lee^4^, Jung Ok Kim^4^, Seok Hoon Jeong^4^, Kyungwon Lee^4^

##### ^1^Department of Laboratory Medicine, Kosin University College of Medicine, Busan, Korea; ^2^Department of Dental Hygiene, Silla University, Busan, Korea; ^3^Yeongam-gun Public Health Center, Yeongam, Korea; ^4^Department of Laboratory Medicine and Research Institute of Bacterial Resistance, Yonsei University College of Medicine, Seoul, Korea

###### **Correspondence:** Dahae Yang (laluna1628@gmail.com)

**Background**

*Enterococcus* species become a leading problem of nosocomial infections with their multidrug resistance (MDR) potential. This study was performed to investigate antimicrobial susceptibility of *E. faecium* and *E. faecalis* clinical isolates in Korea.

**Materials and methods**

A total of 152 non-duplicated *E. faecium* and 112 *E. faecalis* isolates recovered from clinical blood specimen were collected from 19 hospitals at various regions in Korea during 2014. Species identification was performed using MALDI-TOF (Bruker) and 16S rRNA gene sequencing. Antimicrobial susceptibility was tested by CLSI disk diffusion methods. The presence of *vanA* and *vanB* genes was detected by PCR experiments.

**Results**

All *E. faecalis* isolates were susceptible to ampicillin, while only 11.8% (18/152) *E. faecium* isolates were susceptible. *E. faecalis* isolates (96.4%, 108/112) exhibited higher susceptibility rate to vancomycin than *E. faecium* isolates (66.4%, 101/152). None of *E. faecalis* and *E. faecium* isolates showed resistance to linezolid. Only 52.6% (80/152) of *E. faecium* isolates were susceptible to quinupristin-dalfopristin. *E. faecalis* and *E. faecium* isolates exhibited high-level resistance to gentamicin (50% and 57.9%, respectively). PCR experiments showed that all vancomycin-resistant *E. faecium* and *E. faecalis* isolates carried the *vanA*. Interestingly, two vancomycin-susceptible *E. faecalis* and three vancomycin-susceptible *E. faecium* isolates carried the *vanA* gene.

**Conclusions**

This study shows further dissemination of MDR enterococci in Korea. Antimicrobial susceptibility of quinupristin-dalfopristin and gentamicin for *E. faecium* has lowered to near 50%. Discrepancy between antimicrobial susceptibility tests and PCR for *vanA* gene might be due to loss of some elements consisting transposon Tn*1546* carrying the *vanA* gene.

## Session: Antimicrobial Resistance

### AR7 Antifungal susceptibilities, biofilms, phospholipase and protease activities in *Candida rugosa* and *Candida pararugosa* isolated from tertiary teaching hospitals

#### Thiba Peremalo^1^, Priya Madhavan^2^, Sharina Hamzah^1^, Leslie Than^3^, Eng Hwa Wong^2^, Mohd Nasir Mohd Desa^4^, Kee Peng Ng^5^

##### ^1^School of Pharmacy, Taylor’s University Lakeside Campus, Subang Jaya, Selangor Darul Ehsan, Malaysia; ^2^School of Medicine, Taylor’s University, Subang Jaya, Selangor, Malaysia; ^3^Department of Medical Microbiology and Parasitology, Faculty of Medicine and Health Sciences, University Putra Malaysia, Selangor, Malaysia; ^4^Department of Biomedical Sciences, Faculty of Medicine and Health Sciences, University Putra Malaysia, Selangor Darul Ehsan, Malaysia; ^5^Department of Microbiology, Faculty of Medicine, University Malaya Medical Centre, Kuala Lumpur, Malaysia

###### **Correspondence:** Priya Madhavan (Priya.Madhavan@taylors.edu.my)

**Background**

Rising number of candidiasis significantly contribute towards resistance of commonly used antifungal agents. Lately, *Candida* species such as *C. rugosa* and *C. pararugosa* have emerged as fungal pathogens that cause invasive infections.

**Material and methods**

Clinical isolates were from two tertiary referral hospitals in Malaysia. Test for antifungal susceptibility, biofilm, protease and phospholipase activities, all of which contribute to their virulence were performed. Biofilms were quantified using crystal violet (CV) and tetrazolium (XTT) reduction assays in 96-well microtiter plates. Time point reading was done on all strains incubated at 6, 12, 24, 48 and 72 hours.

**Results**

There were seven isolates of *C. rugosa* and one isolate of *C. pararugosa* in this study. E-test antifungal tests showed that all *Candida rugosa* strains were susceptible-dose dependent towards voriconazole and resistant to fluconazole, amphotericin B and caspofungin based on Clinical and Laboratory Standard Institute guidelines. Highest biomass was observed in one of the *C. rugosa* strains, followed by *C. pararugosa* at 12 hours of incubation. However, highest bioactivity was observed in the ATCC at 24 hours, followed by *C. pararugosa* at 48 hours and the same *C. rugosa* train at 24 hours. Virulence was also contributed by secretion of protease enzymes by all the clinical strains. None of the *C. rugosa* and *C. pararugosa* trains showed any phospholipase activity.

**Conclusions**

*C. rugosa* and *C. pararugosa* clinical isolates should be considered pathogenic species because of their resistance against commonly used antifungal drugs and their contributing virulence factors.

## Session: Antimicrobial Resistance

### AR8 Comparison of antimicrobial resistance of respiratory isolates after introduction of a second tier of antimicrobial stewardship program in the medical city hospital

#### Marionne Geronimo, Maria Fe Tayzon, Maria Jesusa Maño

##### Hospital Infection Control and Epidemiology Center, The Medical City, Marilao, Bulacan, Philippines

###### **Correspondence:** Marionne Geronimo (marionnegeronimo@gmail.com)

**Background**

In response to an antimicrobial resistance "apocalypse" The Medical City, a private tertiary hospital in the Philippines, conducts microbiologic surveillance and has an existing "prior approval of restricted antibiotics" wherein release of identified broad spectrum antibiotics were done only upon approval of ID consultants. In June 2015, a second tier of ASP was introduced. The "drug duration, audit and feedback" (DDAF) program monitors and audits the duration of empiric antibiotics prescribed by clinicians. Sticker reminders are being placed on the chart on day 3 as a reminder to de-escalate and on day 10 as a reminder to consider stopping the antibiotics. This study aimed to present a comparative study of the antimicrobial resistance of the top 3 bacteriologic agents from respiratory isolates in a tertiary care hospital in the Philippines, from January to June 2015 versus January to June 2016, as a surrogate marker to the success of the second tier of ASP recently introduced

**Materials and methods**

Most prevalent organisms from sputum, endotracheal aspirate and bronchoalveolar lavage were determined through laboratory surveillance comparing their resistance pattern from January to June 2015 versus January to June 2016.

**Results**

The top 3 respiratory pathogens in the ICU were identified. Some decrease in the resistance data of the most common isolate, *Klebsiella pneumoniae* were as follows: 12% decrease in resistance to ceftriaxone, 9% to levofloxacin and 4% to piperacillin-tazobactam. Similar decreases in resistance were seen with *Pseudomonas aeruginosa* and other organisms.

**Conclusions**

The study showed decrease in resistance of most common bacteriologic agents from respiratory isolates upon introduction of DDAF program.

## Session: Antimicrobial Resistance

### AR9 Do daily universal octenidine baths lead to the development of octenidine resistance in methicillin-resistant *Staphylococcus aureus* (MRSA)?

#### Angela Chow, Pei-Yun Hon, Mar-Kyaw Win, Brenda Ang, Yee-Sin Leo

##### Tan Tock Seng Hospital, Singapore, Singapore

###### **Correspondence:** Angela Chow (Angela_Chow@ttsh.com.sg)

**Background**

Methicillin-resistant *Staphylococcus aureus* (MRSA) colonization has not declined in acute hospitals, despite active surveillance and enhanced contact precautions. Universal antiseptic baths could reduce MRSA transmission, but there are concerns about antiseptic resistance. To evaluate the prevalence of octenidine resistance in MRSA isolated from inpatients exposed to octenidine baths during hospitalization.

**Materials and methods**

We conducted a cross-sectional study, testing for resistance to octenidine in MRSA isolates obtained from discharge screening cultures from inpatients of a dermatology/infectious disease ward from July 2013 to June 2014. During that period, daily universal octenidine baths was implemented in the ward. The minimum inhibitory concentration (MIC) of octenidine was determined by the modified Clinical and Laboratory Standards Institute (CLSI) methods, with microbroth dilution susceptibility testing for a range of 0.125-8 ug/ml. There is no defined MIC breakpoint for antiseptics by CLSI.

**Results**

Of 600 patients screened negative for MRSA on entry to the ward, 21 were identified to be colonized with MRSA at discharge. Eighteen of the isolates were available for resistance testing. Median duration of exposure to octenidine baths was 8.5 (IQR 7-12) days. Majority (77.8%) of isolates had MIC = 0.5, with the remaining had MIC = 1. Duration of exposure to antiseptic baths was not associated with higher octenidine MIC levels (OR 0.84, 95%CI 0.62-1.12, P = 0.236).

**Conclusions**

Resistance to octenidine in MRSA was not observed among patients who were colonized during a hospitalization episode where they were exposed to daily octenidine baths. This study is limited by the small number of MRSA acquisitions and the findings should be verified in larger studies.

## Session: Antimicrobial Resistance

### AR10 Antiseptic susceptibility in methicillin-resistant *Staphylococcus aureus* (MRSA) in subacute wards

#### Angela Chow, Pei-Yun Hon, Tina See, Brenda Ang

##### Tan Tock Seng Hospital, Singapore, Singapore

###### **Correspondence:** Angela Chow (Angela_Chow@ttsh.com.sg)

**Background**

Methicillin-resistant *Staphylococcus aureus* (MRSA) is a growing clinical problem in subacute wards where patients have a longer length of stay than in acute wards. Universal antiseptic baths could be added to the armamentarium for MRSA prevention and control. Our study aimed to assess for the baseline antiseptic susceptibilities in MRSA, prior to the institution of universal antiseptic baths in subacute wards.

**Materials and methods**

We conducted a cross-sectional study, testing for susceptibilities to chlorhexidine and octenidine in MRSA isolates obtained from inpatients of two subacute wards from May-July 2013. Minimum inhibitory concentrations (MICs) of chlorhexidine and octenidine were determined by the modified Clinical and Laboratory Standards Institute (CLSI) methods, with microbroth dilution susceptibility testing for a range of 0.125-8 ug/ml.

**Results**

A total of 43 MRSA isolates were tested: 10 in May, 14 in June, and 19 in July. For chlorhexidine, all except for one had MIC = 2. The remaining with MIC = 4 had occurred in July 2013. In comparison, the majority (90.7%) of the isolates had MIC = 0.5 when tested for octenidine susceptibility, with the remaining having an MIC = 1. A higher proportion of MRSA isolates with the higher MIC level (MIC = 1) to octenidine was observed in June 2013 than in the other months, although statistical significance was not achieved due to the small sample size (OR 7.64, 95%CI 0.72-81.54, P = 0.092).

**Conclusions**

MRSA isolated from patients from subacute wards were highly susceptible to chlorhexidine and octenidine. Universal antiseptic baths could be implemented in subacute wards, with follow-up studies conducted to monitor for any development of antiseptic resistance.

## Session: Antimicrobial Resistance

### AR11 Antimicrobial activity of octenidine against multidrug-resistant gram-negative pathogens

#### Rocio Alvarez Marin^1,2^, Marta Aires de Sousa^3^, Nicolas Kieffer^1^, Patrice Nordmann^1,4^, Laurent Poirel^1^

##### ^1^Medical and Molecular Microbiology Unit, Department of Medicine, University of Fribourg, Fribourg, Switzerland; ^2^Hospital UniversitarioVirgen del Rocio y Virgen Macarena, Seville, Spain; ^3^Escola Superior de Saude da Cruz Vermelha Portuguesa, Lisbon, Portugal; ^4^University of Lausanne and University Hospital Center, Lausanne, Switzerland

###### **Correspondence:** Laurent Poirel (laurent.poirel@unifr.ch)

**Background**

Multidrug-resistant gram-negative (MRGN) pathogens pose a major and growing threat for health care systems, as therapy of infections is often limited due to the lack of available systemic antibiotics. Well tolerated antiseptic molecules may be a very useful implementation in infection control,not only to reduce the dissemination of methicillin-resistant *Staphylococcus aureus* (MRSA), but also MRGN.

**Material and methods**

As decolonization strategies with regard to MRSA are already implemented in high risk areas (i.e. ICUs), this study aimed to investigate, if the same protocol might be concomitantly efficient against MRGN. A series of 5 different species (*Escherichia coli, Klebsiella pneumoniae, Enterobacter cloacae, Acinetobacter baumannii, Pseudomonas aeruginosa*) was studied to prove efficacy under clinically relevant conditions according to an official test norm (EN13727). We used 5 clonally-unrelated isolates per species, including a single wild-type strain, and four MRGN isolates, corresponding either to the 3 MGRN or 4 MGRN definition of multidrug resistance. Octenidine (OCT, Schuelke&Mayr GmbH, Germany) susceptibility was evaluated with and without organic load.

**Results**

A contact time of 30 seconds or 1 minute was fully effective for all isolates by using different OCT concentrations (0.01% and 0.05%), with a bacterial reduction factor of >5 log systematically observed. Growth kinetics were determined with two different wild-type strains (*A. baumannii* and *K. pneumoniae*), proving a time-dependent efficacy of OCT, mirroring what has been previously observed for MRSA.

**Conclusions**

These results highlight that OCT, besides being a very effective agent against MRSA, may also be extremely useful to eradicate emerging highly resistant gram-negative pathogens associated with nosocomial infections.

## Session: Antimicrobial Resistance

### AR12 Comparison of mortality of patients with *Acinetobacter baumannii* bacteremia caused by different levels of drug resistance

#### Wison Laochareonsuk, Sireekul Petyu, Pawin Wanasitchaiwat, Sutasinee Thana, Chollathip Bunyaphongphan, Woranan Boonsomsuk, Pakpoom Maneepongpermpoon, Silom Jamulitrat

##### Prince of Songkla University, Hat Yai, Songkla, Thailand

###### **Correspondence:** Wison Laochareonsuk (ic_conference@yahoo.com)

**Background**

*Acinetobacter baumannii* is an important opportunistic nosocomial pathogen causing a variety of infections. The intrinsic virulence of drug-resistant *A. baumannii* has remained controversial. We compared mortality rates and sepsis score of patients with *A. baumannii* bacteremia caused by different level of drug resistance.

**Materials and methods**

A retrospective study was conducted in adult patients (age > 15 years) admitted to Songklanagarind hospital during 2009 and 2015 and blood culture positive for *A. baumannii* after 3 days of admission. Antimicrobial resistance was categorized into four levels comprising of non-multidrug resistance (non-MDR), multidrug-resistant (MDR), extensively drug-resistant (XDR), and possible pandrug-resistant (possible-PDR). Severity of underlying disease of the patients immediately before onset of bacteremia was determined by sequential organ failure (SOFA) score and American Association of Anesthesia (ASA) score. Virulence of *A. baumannii* was assessed in terms of sepsis score and in hospital mortality rate.

**Results**

The study identified 38, 110, 168, and 14 cases of bacteremia caused by non-MDR, MDR, XDR, and possible PDR, respectively. After adjusting for confounding effect by using Cox proportional hazard model, mortality rates attributable to *A. baumannii* was significantly associated to levels of drug resistance. Using non-MDR as a reference, the incidence rate ratios and corresponding 95% confidence intervals (95% C.I) of MDR, XDR, and possible PDR were 2.3 (95% C.I = 0.9-4.9), 3.1(95% C.I = 1.4-7.0), and 1.9(95% C.I = 0.6-5.5) respectively.

**Conclusions**

The virulence of *A. baumannii* did not loss with drug resistance.

## Session: Antimicrobial Stewardship

### AS1 Antibiotic associated diarrhea in Indian hospitals: common or commonly missed?

#### Dorairajan Sureshkumar, Kalyanaraman Supraja, Soundararajan Sharmila

##### Madras Medical Mission, Chennai, Tamil Nadu, India

###### **Correspondence:** Dorairajan Sureshkumar (dskinfdis@gmail.com)

**Background**

Most of the antibiotic stewardship programs (ASP) in the developing world measure antibiotic consumption, adherence to antibiotic guidelines and antibiotic resistance. However, antibiotic associated diarrhea (AAD) is a common medical problem of antibiotic treatment and important quality monitor of ASP was not monitored commonly in India. This study aimed to measure the prevalence of AAD in hospitalized patients receiving antibiotics.

**Materials and methods**

A point prevalence study was conducted in a 300-bed tertiary care cardiac hospital in Chennai, South India. All hospitalized patients in cardiology wards and intensive care units (ICUs) receiving at least one dose of either oral or intravenous antibiotic were audited by physician assistant for the symptoms of diarrhea and cross checked by interviewing patients.

**Results**

During the study period, 107 eligible patients had available records for analysis. There were 58 patients (54.20%) receiving antibiotics. Of these, there were 34 receiving single antibiotic, 17 receiving two antibiotic combinations, 4 receiving three antibiotic combinations and 3 were taking four antibiotic combinations. The details of diarrhea was missing in 3 patients’ medical records. Only 2 patients (3.44%) developed diarrhea and they received two antibiotics combination.

**Conclusions**

Although more than half of the patients received antibiotics, AAD was not common in our hospital. However, regular monitoring of AAD along with other parameters were required for better implementation of ASP in the hospital.

## Session: Antimicrobial Stewardship

### AS2 Antibiotic prescription after restricted antimicrobial program

#### Cucunawangsih Cucunawangsih^1^, Benny Setiawan^2^, Nicolaski Lumbuun^3^

##### ^1^Department of Microbiology, Faculty of Medicine, University of Pelita Harapan,Tangerang, Banten, Indonesia; ^2^Department of Pharmacy, Siloam Teaching Hospitals, Lippo Village, Tangerang, Indonesia; ^3^Department of Internal Medicine, Faculty of Medicine, University of Pelita Harapan, Tangerang, Banten,Indonesia

###### **Correspondence:** Cucunawangsih Cucunawangsih (cucunawangsih.fk@uph.edu)

**Background**

Infections caused by antibiotic-resistant bacteria have led to increase burden on the healthcare system. Effective antimicrobial stewardship control program (ASCP) requires the clinician acceptance of program recommendation. We evaluate the antibiotic consumption and antibiotic susceptibility after ASCP implementation in 2013 in a teaching hospital in Tangerang, Indonesia.

**Materials and methods**

Our ASCP restrict the prescription of carbapenems, fourth generation cephalosporins, and tigecycline. Antibioticsusceptibility and consumption of restricted antibiotics were extracted from database. Antibiotics use was measured by the number of DDDs per 100 bed-days

**Results**

The proportion of susceptible bacteria against; cefpirome increased from 57% to 73%, cefepime 63% to 64%, imipenem 78% to 83%, and tigecycline 74% to 75% during 2013 to 2015. The proportion of meropenem susceptibility remained the same at 70% in 2013 and 2015. The defined daily doses (DDDs) per 100 bed-days was significantly reduced in all restricted antibiotics from 2013 to 2015 except tigecycline. The consumption of 1.0 g cefepime was 2190, 1035 and 107, 1.0 g cefpirome was 313, 473 and 140, 1.0 g meropenem was 16047, 11271 and 5281, 0.05 g teicoplanin was 40.4, 58.8 and 0, 0.5 g vancomycin was 716, 731 and 212.5, and 0.6 g linezolid was 129.6, 72 and 0 in 2013, 2014 and 2015, respectively

**Conclusions**

Our ASCP was effective in terms of lowering broad spectrum antibiotic consumption and improving the antibiotic susceptibility.

## Session: Antimicrobial Stewardship

### AS3 Utilization of diagnosis–procedure combination data for advancing the antimicrobial stewardship program

#### Haruo Nakayama, Toshiko Ota, Naoko Shirane, Chikako Matuoka, Kentaro Kodama, Masanobu Ohtsuka

##### Toho University Ohashi medical center, Tokyo, Japan

###### **Correspondence:** Haruo Nakayama (haruonakayama@med.toho-u.ac.jp)

**Background**

Infection with antibiotic-resistant bacteria results in increased morbidity, mortality and economic burden. Antimicrobial stewardship program (ASP) has been widely implemented to guide appropriate antibiotic use, in order to minimize antibiotic resistance. However, establishment of ASP is not always possible due to lack of interest. We examined the application of diagnosis-procedure combination (DPC) data as an incentive for achieving the target of ASP.

**Materials and methods**

The Toho University Ohashi Medical Center inpatient initiated ASP focusing on reduction of inappropriate perioperative antibiotics and anti-MRSA drugs. The DPC data was extracted for antibiotic consumption and duration in each patient from April 2013 to March 2016.

**Results**

The consumption of the first-generation and second-generation cephalosporins as perioperative antimicrobial agents was 62% during observation period. This proportion was below the initial benchmark. On the other hand, the consumption of anti-MRSA agents was 2.26% higher than the benchmark. More than half of patients undergoing surgery received perioperative antibiotics only one day. The majority was cardiovascular surgery patients used intraoperatively.

**Conclusions**

Our study shows that utilization of the DPC database for advancing the ASP is possible. It is convenient process to measure outcome of ASP at the group or organizational level.

## Session: Antimicrobial Stewardship

### AS4 Impact of antimicrobial stewardship on prescribing practices of physicians, antibiotic utilization and rates of multiple drug resistant organisms in Manila Doctors hospital, Philippines

#### Silverose Ann Andales Bacolcol, Melecia Velmonte, Allan Alde, Keithleen Chavez, Arlene Joy Esteban, Aisa Jensen Lee

##### Manila Doctors Hospital, Manila, Philippines

###### **Correspondence:** Silverose Ann Andales Bacolcol (silveroseann@yahoo.com)

**Background**

Manila Doctors Hospital (MDH), a tertiary hospital in the Philippines had initiated antimicrobial stewardship (AMS) to reduce antimicrobial resistance(AMR) in the institution. The impact of AMS on prescribing practices of physicians, antibiotic utilization and rates of multiple drug resistant organisms (MDROs) were evaluated.

**Materials and methods**

From 2011 to 2013, a multi-disciplinary AMS team initiated educational campaigns, developed AMS policies and antimicrobial guidelines based on the local antibiogram. Pilot implementation of policies was conducted in 2014 to 2016. Outcome measures included compliance rate of physicians to the surgical prophylaxis guideline, utilization of carbapenems and vancomycin in defined daily doses (DDDs per 100 patient-days) and rates of multi-drug resistant organisms.

**Results**

The utilization of carbapenems was 47.48, and 31.72 DDDs/100 patient days in 2014 and 2015, respectively (33% reduction). The utilization of vancomycin increased from 3.76 DDDs/100 patient-days in 2014 to 4.93 DDDs/100 patient-days in 2015. There was increased compliance to timing of surgical prophylaxis from 53% in 2014 to 89% in 2016. The proportion of Methicillin-resistant *S. aureus* (MRSA) decreased from 60% in 2013 to 47% in 2015. The proportion of Extended spectrum beta-lactamase (ESBL)-producing *K. pneumoniae* decreased from 12% in 2013 to 10% in 2015, whereas proportion of ESBL-producing *E. coli* increased from 11% in 2013, to 20% in 2014, and 23% in 2015. The proportion of *Klebsiella pneumoniae* carbapenemase (KPC)-producing *K. pneumoniae* was 16% in 2013, 23% in 2014, and 16% in 2015.

**Conclusions**

The AMS program resulted in increased compliance to the surgical prophylaxis policy, decreased utilization of carbapenems, and decreased proportion of MRSA, ESBL-producing *K. pneumoniae*. There is a room for improvement in reduction vancomycin use in this setting.

## Session: Antimicrobial Stewardship

### AS5 Comparison of the clinical efficacy between aerosolized colistin methanesulfonate plus tigecycline and aerosolized colistin methanesulfonate alone against extensively drug-resistant *Acinetobacter calcoaceticus –baumannii* complex pneumonia

#### Tai-Chin Hsieh^3^, Shio-Shin Jean^1,2^

##### ^1^Emergency Medicine, Department of Emergency and Critical Care Medicine, Wan Fang Medical Center, Taipei Medical University, Taipei, Taiwan; ^2^Department of emergency and critical care medicine, School of Medicine, College of Medicine, Taipei Medical University, Taipei , Taiwan; ^3^Division of Infectious Diseases, Department of Internal Medicine, Shin Kong Wu Ho-Su Memorial Hospital,Taipei, Taiwan

###### **Correspondence:** Shio-Shin Jean (jeanshioshin168@gmail.com)

**Background**

Extensively drug-resistant *Acinetobacter calcoaceticus-baumannii complex* (XDR-ABc) pneumonia is an important cause of healthcare-associated pneumonia. Although tigecycline was not approved for treatment of healthcare-associated pneumonia, it has been used off-label for XDR-ABc pneumonia. We evaluated whether the clinical efficacy of tigecycline combined with aerosolized colistin methanesulfonate (CMS) is superior to aerosolized CMS alone.

**Materials and methods**

This is a retrospective case-control study, conducted in Wan-Fang Medical Center, Taipei Medical University, Taipei, Taiwan from November 2014 to February 2015. The definition of XDR-ABc pneumonia was pneumonia caused by ABc with susceptibility only to colistin and tigecycline. Cases were patients who received aerosolized CMS in combination with intravenous tigecycline for at least 5 days to treat XDR-ABc pneumonia. Controls were those who received inhaled CMS alone and were selected based on the following matching criteria to cases; age (±5 years), Acute Physiology and Chronic Health Evaluation (APACHE) II score (±4 points).

**Results**

There were 53 patients in each group. The mean age of patients was 80 years old. The proportion of patients underwent mechanical ventilation were 35.8% and 28.3% in cases and controls, respectively (*p* = 0.45). The median APACHE II score was 17.5 (15.3-19.6) in cases and 17.3 (15.3-19.2) in control group (*p* = 0.9). The mean length of hospital stay was 32 days (*p* = 0.524), 30-days mortality rate was 34% and 22.6%(*p* = 0.02), and overall mortality was 46.2% and 33.3% (p = 0.19)in cases and controls, respectively.

**Conclusions**

Despite the active in-vitro susceptibility of tigecycline against XDR-ABc, combination therapy with tigecycline and aerosolized CMS for XDR-ABc pneumonia showed no additional clinical benefit.

## Session: Antimicrobial Stewardship

### AS6

**Withdrawn**

## Session: Antimicrobial Stewardship

### AS7 The effectiveness of reducing the amount of antibiotic resistant strains by Promoting Antibiotic Stewardship Program of a medical center

#### Huey-Jen Huang^1^, Shu-Ju Huang^1^,Yu-Huan Huang^1^,Pei-Chen Cheng^1^, Su-Fang Yu^1^, Shih-Ming Tsao^1,2^, Yuan-Ti Lee^1,2^,Chien-Feng Li^1,2^, Min-Chi Lu^3^

##### ^1^Infection Control Team, Chung Shan Medical University Hospital, Taichung, Taiwan; ^2^Infectious Diseases Division, Chung Shan Medical University Hospital, Taichung, Taiwan; ^3^Infectious Diseases Division, China Medical University Hospital, Taichung, Taiwan

###### **Correspondence:** Huey-Jen Huang (vivian.sunia@gmail.com)

**Background**

In 2013, the amount of antibiotics in this medical center accounted for 8.4% of its total drug amount. The calculated DID dosage of inpatient antibiotics was 917 and the rate of antibiotic-resistant strains has been on the rise. To cope with the development of drug resistance, from 2014 to 2015, the Antibiotic Stewardship Program (ASP) was executed to promote proper use and therefore to decrease the volume of antibiotics, and to reinforce MDRO isolation.

**Materials and methods**

A multi-discipline team for antibiotic stewardship was reformed. ID doctors and infection control practitioners developed regulations for antibiotic use, conducted training programs, reviewed antibiotic uses and gave feedback. The compliance and accuracy of hand hygiene, and isolation precaution and protection were strengthened by nursing personnel. The pharmacist team division provided daily antibiotic assessment and statistics. Microbiology laboratory was responsible for drug-resistant data.

**Results**

The expenditure of consumed antibiotic, as a ratio of total drug, declined from 8.4% in 2013 to 5.6% in 2015. From 2013 to 2015, the DID from 917 to 824, carbapenems from 53.4 to 47.4, quinolones from 95.6 to 81.9, and glycopeptides from 28.4 to 21.9. Furthermore, CRPA from 16.7% to 5.7%, CRAB from 55.7% to 26.2%, MRSA from 52.2% to 43.5%, VRE from 60% to 45%. However, a little elevation of CRKP from 9.62% to 11.3% was observed.

**Conclusions**

Employing ASP, we have enhanced the cooperation among antibiotic team members. As a result, the correct use of antibiotics was improved, the amount of antibiotics was less consumed, and the ratios of most MDRO declined.

## Session: Antimicrobial Stewardship

### AS8 Inappropriate empiric antibiotic treatment of uncomplicated cystitis: a prospective observational study in a tertiary care center in Thailand

#### Nattapol Pruetpongpun^1^, Thana Khawcharoenporn^1^, Pansachee Damronglerd^1^, Nuntra Suwantarat^2^, Anucha Apisarnthanarak^2^, Sasinuch Rutjanawech^2^

##### ^1^Division of Infectious Diseases, Faculty of Medicine, Thammasat University, Bangkok, Thailand; ^2^Chulabhorn International College of Medicine, Thammasat University, Bangkok, Thailand

###### **Correspondence:** Nattapol Pruetpongpun (Natprathan@hotmail.com)

**Background**

Increased antibiotic resistance among *Escherichia coli* has led to inappropriate empirical antibiotic use (IAU) for associated infections. Limited data exists for IAU among cases with acute uncomplicated cystitis (AUC).

**Material and methods**

We conducted a prospective observational study at a General Practice (GP) Clinic from December 2014 to February 2016. Eligible participants included women aged 15-60 years with AUC. All participants’ urine cultures were sent before empirical antibiotics were prescribed at the GP physicians’ discretion. The rate of IAU was subsequently identified by the investigators. Strategies to minimize IAU were then determined based on the relevant data of AUC treatment in this study.

**Results**

Eighty participants were enrolled. *E. coli* was the most common pathogen isolated (78.3%) with resistance rates to trimethoprim-sulfamethoxazole, fluoroquinolone, ceftriaxone, amoxicillin-clavulanate and ertapenemof61.7%, 42.6%, 21.3%, 2.1% and 0%, respectively. Extended-beta-lactamase production was confirmed in 12.5% of *E. coli* isolates. The rate of IAU was 91.3%. Ciprofloxacin use was the only independent risk factor for IAU (adjusted odds ratio, 6.471; 1.089-38.461; *P* =0.04). Based on the study results, including the in-vitro susceptibility data and the risk factors for acquisition of antibiotic-resistant *E.coli*, a specific algorithm for AUC treatment was created. If this algorithm was used along with education about IAU and antibiotic stewardship program focusing on ciprofloxacin use, the rate of IAU would have decreased to 3.8%.

**Conclusions**

Our findings suggest the high rate of IAU in AUC treatment in a GP setting and underlie the need for multifaceted interventions to reduce IAU.

## Session: Antimicrobial Stewardship

### AS9 Synergy between antimicrobial stewardship programs and infection control efforts

#### Lisa Cushinotto, Patty McBride, Harding Williams Jr, Hans Liu

##### Bryn Mawr Hospital, MainLine Health Care, Bryn Mawr, Pennsylvania, USA

###### **Correspondence:** Hans Liu (liuliang@aol.com)

**Background**

Infection control/prevention (IC) programs seek to prevent infections via surveillance and outbreak investigation, hand hygiene, isolation precautions, environmental disinfection, evaluating IC products, and policy development. Antimicrobial stewardship programs (ASP) try to optimize antibiotic use for better outcomes and less toxicity. Both reduce antimicrobial resistance in pathogens, educate healthcare staff, patients, and families, and optimize resource use.

**Material and methods**

Our study objectives were to define areas of synergy between IC and ASP efforts with specific examples and to determine how best to promote these.

Bryn Mawr Hospital: 250-bed community-teaching hospital with 3 IC practitioners; ASP team has one infectious diseases (ID) physician and an ID-trained Pharm.D. It is part of a 5 hospital system with microbiology laboratory. ASP has been in placed for 5-1/2 years.

Synergy between ASP and IC: (1) collaborative identification of outbreaks (e.g., regional babesiosis in 2015, ongoing *C. difficile* cases), (2) monitoring antimicrobial resistance via complementary computer surveillance systems, (3) coordinating presentation of microbiology antibiograms, (4) collaboration on healthcare staff educational programs, and (5) joint presentations to system wide committees and accrediting organizations.

**Results**

From 2011 to 2015 dosage days for selected antibiotics decreased 58-90% per 1000 patient-days and total antibiotic cost decreased $577,680 (54%) per year.

**Conclusions**

IC and ASP programs should work together to the benefit of both and the institution and health system as a whole. This can be facilitated by regular communications and meetings, ongoing review of microbiological pathogen and susceptibility trends, and collaboration on research and educational programs.

## Session: Antimicrobial Stewardship

### AS10 Awareness survey regarding antibiotic among healthcare workers in Hungvuong hospital

#### Phan Thi Hang^1^, Dinh Pham Phuong Anh^2^

##### ^1^Quality management division in Hung Vuong hospital, Ho Chi Minh, Viet Nam; ^2^Infection control department in Hung Vuong Hospital, Ho Chi Minh, Vietnam

###### **Correspondence:** Dinh Pham Phuong Anh (thuyhangytcc@gmail.com)

**Background**

Healthcare workers (HCWs) play an important role to be a consultant about antibiotic use for patients. This would be helpful to reduce antibiotic overuse and prevent emergence of antimicrobial resistance in the hospital and public settings.

**Materials and methods**

We conducted across sectional study by using a self-assessment validated questionnaires, to determine current awareness and common habits related to antibiotic usage and antimicrobial resistance among HCWs in Hung Vuong hospital; an obstetrics and gynecology hospital in Vietnam.

**Results**

A total of 161 HCWs were enrolled in the survey. Although 99% of HCWs responded correctly "Many infections are becoming increasingly resistant to treatment by antibiotics", 77% of them thought that “antibiotic resistance occurs when your body becomes resistant to antibiotics”. A total of 19% (95% CI: 0.13 – 0.26) and 8% (95% CI: 0.04 – 0.13) of HCWs have correct knowledge about antibiotic usage and antimicrobial resistance. A total of 16% of HCWs answered that they have to prescribe antibiotic because they cannot follow up the patients' condition. Twenty-two percent of HCWs answered that it is necessary to take antibiotic when people have fever.

**Conclusions**

Majority of HCWs have incorrectly awareness about definition of antibiotic resistance. Furthermore, their habit of antibiotic use is inappropriate. We should have intervention programs to improve HCWs’ knowledge and control their antibiotic usage*.*

## Session: Antimicrobial Stewardship

### AS11 High prevalence of HAIs caused by gram negative carbapenem resistant strains in Vietnamese paediatric ICUs

#### Ngai Le, Dung Khu, Lam Nguyen

##### National Children's Hospital, Dong Da, Hanoi, Viet Nam

###### **Correspondence:** Ngai Le (ngai.lekien@nhp.org.vn)

**Background**

It is necessary to understand on the prevalence of hospital acquired infections and carbapenem-resistant gram negative bacteria (CR-GNB) in children’s hospital in resource constrained settings.

**Material and methods**

During a 1-year study period (2012-2013), we conducted a monthly point prevalence surveys (PPS) using the protocol of ECDC in 2 major Vietnamese children’s hospitals and 3 referral hospitals with pediatric intensive care units (PICU).

**Results**

1363 cases were included in the PPS. Average age was 11 months (median 3 months). Major reasons for admission were infection 66%. Admission source was other hospital 49.3%, current hospital 36.5%. Intubation 47.8%, CVC 29.4%, PVC 86.2%. HAI prevalence was 33.1%, of these 2 HAI in 9.9% and 3 HAI in 1.3%. Intubation had a significant (p < 0.001) correlation with HAI. The most common diagnosis was pneumonia 52.2%, sepsis 26.4%, the SSI and NE 9 cases (2%) each. Positive laboratory findings were 212 (43%). Carbapenem resistance were 40%; 46% of *K. pneumoniae* were carbapenem-resistant and 76% cephalosporin-resistant. 56% of *P. aeruginosa* and 64% of *A. baumannii* were carbapenem-resistant; 78% of *S. aureus* were MRSA. Antibiotics were given to 1307 (88.6%), one antibiotic 39.9%, 2 antibiotics 44.7%, 3 antibiotics 10.6% and 4 antibiotics 0.8%, average 1.7 antibiotics per case. Colistin was given to 97 cases, of these 49% had reported carbapenem-resistant strains; 49% carbapenem-resistant cases were treated with colistin.

**Conclusions**

There is a high prevalence of HAI in Vietnamese PICUs, usually caused by GNB with a very high antibiotic resistant rate. Colistin is commonly used in combination with other antibiotics to treat these resistant infections.

## Session: Disinfection/ sterilization

### DS1 Processing of reusable medical devices in health service organizations: preventing HAIs at Macquarie University hospital: an Australian experience

#### Roel Beltran Castillo

##### Central Sterilizing Services Department, Manager Macquarie University Hospital, North Ryde, New South Wales, Australia

###### **Correspondence:** Roel Beltran Castillo (roel.castillo@muh.org.au)

**Background**

The global community demands standardization to approaches in patient safety. Protocols differ to meet the specific demands of health care facilities; patient safety has always been the common end. An introduction and understanding of compliance under the Australian setting would reaffirm this common end. Our similarities and differences in achieving the purpose are interesting to note.This study aimed to share information between infection control professionals, how Australia kept pace with the technological evolution of reusable medical devices (RMDs) and caters to specific reprocessing requirements to ensure patient safety. How RMDs are reprocessed to minimize, control and prevent healthcare-associated infections (HAIs)

**Materials and methods**

The Australian Commission on Safety and Quality in Healthcare requires health service organizations to comply with 10 standards ensuring patients get the quality of care they truly deserve. All CSSD throughout Australia are responsible for:

Standard 3.16: Reprocessing of RMDs in accordance with relevant International standards and instructions for reprocessing or IFUs;

Standard 3.17: Systems to enable identification of a process to a patient;

Standard 3.18: Ensuring workforces to decontaminate RMDs undertake competency based training.

Are RMDs reprocessed to minimize the risk of infection at Macquarie University Hospital?

**Results**

Macquarie University Hospital: ISO accredited and CSSD received on all criteria: Meet with Merit – this is the highest possible achievement on an ISO accreditation

**Conclusions**

Prevention of healthcare associated infections in patients undergoing surgeries is an essential component of patient safety. Compliance with standards is a critical aspect.

## Session: Disinfection/ sterilization

### DS2 Fumigation free operating rooms in developing world- a reality

#### Dorairajan Sureshkumar^1^, Ram Gopalakrishnan^1^, Venkatasubramanian Ramasubramanian^1^, Subramanian Sreevidya^2^, Ranganathan Jayapradha^2^

##### ^1^Apollo Hospitals, Chennai, Tamil Nadu, India; ^2^Apollo Hospitals, Karapakkam, Tamil Nadu, India

###### **Correspondence:** Dorairajan Sureshkumar (dskinfdis@gmail.com)

**Background**

Fumigation of operating rooms (OR) with high concentration of toxic chemicals is an age old tradition practiced in most of the developing world to control hospital acquired infections. This approach lost favor in the developed world due to questionable efficacy and toxicity concerns. However, most of the hospitals in developing world continue to use fumigation practices with variable frequency. Here we report our experience of fumigation free OR in India.

**Materials and methods**

This quasi-experimental before and after intervention study was conducted in a 50-bed tertiary care referral women and children hospital in Chennai (South India) between January 2015 and September 2016. The practice of OR disinfection using quaternary ammonium compounds fumigation was allowed in addition to standard cleaning methods in before-intervention phase (Jan 2015 to Dec 2015). In after-intervention phase (Jan 2016) onwards the fumigation practice was stopped and standard cleaning methods alone followed. The monthly environmental microbiological surveillance cultures and surgical site infection (SSI) rates were compared and analyzed.

**Results**

In the before-intervention phase there were 715 surgical procedures were carried out with 2 SSIs and 156 environmental samples tested all were within acceptable limits as per defined standards. In the after-intervention phase 535 surgeries were carried out with no SSIs and all 117 environmental samples collected were within acceptable limits.

**Conclusions**

The standard cleaning methods alone without chemical fumigation is sufficient for operating rooms disinfection in India. However, this finding should be confirmed in large multi-site studies before universal recommendation.

## Session: Disinfection/ sterilization

### DS3 Change of the contamination rate caused by skin antiseptic change during blood culture collection

#### Atsushi Umetsu, Tetsuhiro Noda, Kenyuu Hashimoto, Akihiro Hayashi, Mikie Kabashima

##### Shin Koga Hospital, Kurume, Fukuoka, Japan

###### **Correspondence:** Atsushi Umetsu (spas3bq9@future.ocn.ne.jp)

**Background**

It is reported that skin antiseptic with a chlorhexidine-alcohol concentration of more than 0.5% usedduring blood cultures lowers contamination rate more effectively than povidone-iodine skin antiseptic. As an approach to enhance precision of blood cultures, I heldcampaigns for appropriate sterilization methods in 2010 also changed 10% povidone-iodine to 1% chlorhexidine-alcohol antiseptic during blood cultures in 2012, and reported results.

**Material and methods**

Study periods:April,2010-March,2011(A), August,2011-July,2012 after the sterilization methods campaign(B), and August,2012-July,2013 after the antiseptic change(C). I investigated and compared each period. The contamination was calculated by dividing the number of cases in which there was only one positive result of two sets or more of blood culture specimens submitted on the same day by the total of all paired sets collected. The contaminants were defined as CoNS, *Bacillus* spp., *Corynebacterium* spp., *Micrococcus* spp., *Propinonibacterium* spp. which a doctor took as causative organism of the infection were excluded.

**Results**

The contamination of the blood culture for periods A: 2.25%, B: 0.98%, and C: 1.05%.

**Conclusions**

There’s significant decrease in the contamination during blood cultures after sterilization methods campaign (A-B)(p < 0.05). It suggests contamination decreased by performing appropriate sterilization methods. There were no contamination differences in B-C after changing disinfectant (p = 0.86). It was suggested that an equal skin sterilization effect was provided when I performed the sterilization with1% chlorhexidine-alcohol and10% povidone-iodine, which was appropriate.The 1% chlorhexidine-alcohol has an immediate effect and durability in comparison with povidone-iodine, shorting the time for drawing blood after sterilization. Reflecting importance for busy on-site blood cultures.

## Session: Disinfection/ sterilization

### DS4 Flexible endoscope decontamination - is it good enough?

#### Ursula Jadczak^1^, Knut Elvelund^1^, Marit Johnsen^1^, Bente Borgen^2^, Egil Lingaas^2^

##### ^1^Lovisenberg Diakonale Hospital, Oslo, Norway; ^2^Oslo Univerisity Hospital, Oslo, Norway

###### **Correspondence:** Ursula Jadczak (ursula.agnes@yahoo.no)

**Background**

Lovisenberg Diakonale Hospital has Olympus EDT machines for cleaning of endoscopes. In the standard program the wash time is 3 minutes with special detergent and the disinfection time is 5 minutes. Periodical testing of final rinse-water is performed regularly with good results. Microbiological control of the final rinse-water from the washer-disinfector for endoscopes (EWD) is the most widely used methods for detecting growth of bacteria. The validation process is a comprehensive procedure that requires both resources, time, knowledge, special equipment and access to the microbiological laboratory. The hospital wanted to assure the quality of decontamination of their endoscopes. We have established a partnership with the Department of Infection Control at Oslo University Hospital in order to validate the EWD.

**Materials and methods**

The decontamination of flexible endoscopes must be tested and validated according to the standard EN ISO 15883. To validate the disinfection process and secure a repeatable method, we used a surrogate endoscope (Spypach). This is equipped with biological indicators, temperature sensors and pressure- and flow-measures.

**Results**

Microbiological control of final rinse-water: satisfactory results according to standard.Cultivation of biological indicators: unsatisfactory results according to standard. Discovery of protein and fibrin in surrogate endoscope: unsatisfactory results according to standard. Measurement of temperature, pressure and flow: no deviation

**Conclusions**

The endoscope was not adequately cleaned during decontamination. Final rinse-water had satisfactory quality but remaining biological indicators and protein residues showed that the decontamination was not satisfactory. Alternative solutions may include: increasing the wash time and/ or modify the contents of detergent.

## Session: Disinfection/ sterilization

### DS5 Can we use the super rapid readout biological indicator to replace traditional biological indicator

#### Chia-Hua Mao^1^, Fu-Chieh Chang^2^, Chang-Pan Liu^2^

##### ^1^Central Supply Room, Department of Nursing, Mackay memorial hospital, Taipei, Taiwan; ^2^Infection control center, Mackay memorial hospital, Taipei, Taiwan

###### **Correspondence:** Fu-Chieh Chang (maple0711@gmail.com)

**Background**

As we know, the biological indicators are the most accepted means of the QC in sterilization. Because the biological indicators contain *Geobacillus stearothermophilus* that have spore inside the cell, if the sterilization were successful, the *G.sterothermophilus* should be killed, and the Biological indicator can’t detected by color or machine. So, in this study, we aimed to make sure whether the super Rapid Biological Indicator can replace the traditional Biological Indicator or not. According to the instruction of the super Biological Indicator, after 60 minutes, the data will show pass or not. So we using the culture method to make sure the result are the same or not.

**Materials and methods**

In this study, we collected 214 super Rapid Readout Biological Indicators from Feb.2016 to Mar. 2016, and all the result showed that was no bacteria growth after 1 hour incubated. Then we used thioglycolate and TSA to make sure the results .

**Results**

According to the experiment’s result, all of the culture data showed that 214 super Rapid Readout Biological Indicators were the same. The accuracy were 100% and the sensitivity and specificity were 100% and 100%.

**Conclusions**

Based on this study, we assented that super Rapid Readout Biological Indicator can replace the traditional Biological Indicator. Otherwise, BI results are available about the time (1 hour) it takes to cool a load, and instruments and implants can be released to the OR much sooner

## Session: Disinfection/ sterilization

### DS6 Using crepe paper to substitute cotton in sterilization

#### Ru-Hui Chao^1^, Fu-chieh Chang^2^, Chang-pan Liu^2^

##### ^1^Central Supply Room, Department of Nursing, Mackay memorial hospital, Taipei, Taiwan; ^2^Infection control center, Mackay memorial hospital, Taipei, Taiwan

###### **Correspondence:** Fu-chieh Chang (maple0711@gmail.com)

**Background**

Sterilization wrap is commonly used for instrument trays or cassettes. There are many different types and sizes of wraps available. Typically, two sheets are needed to provide an effective barrier and a specific technique is recommended [CDC, AAMI ST79] to allow for aseptic opening. Wrapped instruments should be secured with sterilization tape that also serves as an external indicator. Before closing, a multi-parameter chemical indicator should be included inside along with the instruments. Before this study, we used cotton of wraps. And the problem we focused was the expiry date. According to the Taiwan CDC suggestion, the cotton’s expiry date is 7 days. But it’s too short for us. This study aimed to find some types that can replace the cotton.

**Materials and methods**

In this study, we used the crepe paper of wraps to replace cotton. Otherwise, we want to elongate the expiry date. So we collected the instruments that cover by crepe paper and that storage in CSR (HEPA level: 100000) for 1, 4, 8, 12, 16, 20, 24, 28 weeks. Then we used the broth methods to make sure whether the instruments were contamination or not.

**Results**

In this study, we found all of the instruments were clean on 20 weeks. After 20 weeks, the culture result showed that some bacteria were growth on the instruments.

**Conclusions**

According to the experiment’s results, we suggested that the crepe paper can replace the cotton of wraps, and it could elongate the expiry date for 20 weeks in our CSR.

## Session: Disinfection/ sterilization

### DS7 Development of the sterilization process to prevent the breakage of mouth mirror in central sterile supply department at Wanonniwas hospital

#### Junpen Pawapotako

##### Wanonniwas Hospital, Wanonniwas, Sakonnakhon, Thailand

###### **Correspondence:** Junpen Pawapotako (peemon2009@hotmail.com)

**Background**

In 2014, 47 pieces of the mouth mirrors from dental unit were broken as they were packed and mixed with the other tools that were heavy, shape, and without protection. It broke the mouth mirrors from a process of packaging. The important is to prevent the broke of mouth mirror in order to have the good quality and enough instruments to service patients.This study aimed to determine the result of development of sterilization process toprevent the broke of mouth mirror in CSSD of Wanonniwas hospital.

**Materials and methods**

The sample group was composed of 1) Mouth mirror and 2) Personnel from the CSSD 8 persons.The process included 3 steps: 1) prepare process is to learn the reason of the breaking of mouth mirror and create the system in every process from caring, washing, packaging, sterilization, keeping, sending to dental unit, 2) methods process is inform the personnel about the reason, why mouth mirror were broken, change the methods of sterilization of mouth mirror, and 3) evaluation : This study were collected during fiscal year 2014 through 2016.

**Results**

The breaking of mouth mirror in year 2014, 2015, and 2016 (May) were 48 pieces, 33, and 4 pieces, respectively. It has a clearly decrease.

**Conclusions**

The sterilization to prevent the broke of mouth mirror that caring keep the mouth mirror into the box with a lid. The process of washing and drying, sorting the mouth mirror. Packing cloth wrapped. Sterilization, distribution and storage kit for the side impact protection.

## Session: Disinfection/ sterilization

### DS8 Evaluation on the cleaning performance of different cleaning processes using the protein residue check test

#### Chadanan Prasertpan^1^, Wantanee Malaihuan^1^, Phisit Uirungroj^2^

##### ^1^CSSD, Maharaj Nakorn Chiang Mai Hospital, Faculty of Medicine, Chiang Mai University, Chiang Mai, Thailand; ^2^Pose Health Care Co., Ltd., Kannayao, Bangkok, Thailand

###### **Correspondence:** Chadanan Prasertpan (cprasert.morn@gmail.com)

**Background**

The medical instrument cleaning and disinfection procedures are important process to remove organic and inorganic matter, due to concerns about contamination risks especially protein residue, to prevent cross-contamination and ensure the safety. This study aimed to determine the cleaning efficacy of two different cleaning methods, manual cleaning process and an automatic washer disinfector machine, using the protein residue check test in the Central Sterile Supply Department (CSSD), Maharaj Nakorn Chiang Mai Hospital.

**Materials and methods**

The 35, 40, and 25 samples of medical instrument washed and cleaned by manual cleaning process, the automatic washer disinfector machine, and re-sterile (without washing process), respectively, were collected from July to August, 2014 to determine the protein residue. The protein residue was detected with two different detections, the protein residue check test (Pose Health Care Co., Ltd., Thailand) and fluorescence-based protein detection test (Lab Focus Co., Ltd.).

**Results**

The 27 (77.14%), 33 (82.50%), and 11 (44.00%) samples of manual cleaning process, the automatic washer disinfector machine, and re-sterile produced undetectable protein residues, respectively (p-value = 0.002). The 8 (22.86%), 7 (17.50%), and 14 (56.00%) samples of manual cleaning process, the automatic washer disinfector machine, and re-sterile produced detectable protein residues, respectively.

**Conclusions**

The results indicated that, the medical instrument using the re-sterile process should pre-clean instrument with enzymatic detergent to remove gross soil immediately and must be thoroughly washed and cleaned before being sterile. Moreover, the manual cleaning process and an automatic washer disinfector machine must be optimized to eliminate protein contamination and minimize the cross-contamination.

## Session: Disinfection/ sterilization

### DS9 Evaluation on the cleaning performance of an alkaline detergent used in an automatic washer disinfector machine

#### Chadanan Prasertpan^1^, Chalermpong Saenjum^2^, Teerapat Ouirungrog^3^, Phisit Uirungroj^3^

##### ^1^Central sterile services department (CSSD), Maharaj Nakorn Chiang Mai Hospital, Faculty of Medicine, Chiang Mai University, Chiang Mai, Thailand; ^2^Department of Pharmaceutical Sciences, Faculty of Pharmacy, Chiang Mai University, Chiang Mai, Thailand; ^3^Pose Health Care Co., Ltd., Kannayao, Bangkok, Thailand

###### **Correspondence:** Chalermpong Saenjum (chalermpong.saenjum@gmail.com)

**Background**

Different medical device cleaning and disinfection procedures are used on a large scale. It is an important procedure to remove organic and inorganic from medical device to prevent cross-contamination. This study aimed to determine the cleaning efficacy of an alkaline detergent used in an automatic washer disinfector machine in the Central Sterile Supply Department (CSSD), Maharaj Nakorn Chiang Mai Hospital.

**Materials and methods**

An alkaline detergent was developed by Pose Health Care Co., Ltd., Thailand. The programs were designed with different concentrations of alkaline detergent 50, 60, 80, and 100 mL (0.15, 0.18, 0.24, and 0.30 v/v, respectively) and two different temperatures (60 and 65 °C). The cleaning efficacy was monitored using a TOSI (EN ISO 15883) and Brown STF loaded check strips. Each program used three TOSI test kits and five Brown STF strips to evaluate the cleaning efficacy. Additionally, protein residue was detected with the Pyromol® test, protein residue check test, and fluorescence-based protein detection test. Moreover, biofilm and microorganisms were determined using a scanning electron microscope (SEM).

**Results**

The optimum concentration of the alkaline-based cleaning detergent and temperature were 60 mL (0.18 v/v) and 60 °C, respectively. This program (60 mL and 60 °C) produced undetectable protein residues, biofilm, and microorganisms on medical instruments after the cleaning process.

**Conclusions**

Comparable to the recent condition of 100 mL (0.30 v/v) concentration and at 65 °C, the optimum condition of 60 mL concentration and at 60 °C reduced the cleaning costs by 40% per each cleaning process.

## Session: Emerging Infectious Diseases

### EID1 Implementing standardised guidelines for management of carbapenemase-producing enterobacteriaceae in Australia: experience of a large tertiary healthcare facility

#### Sue Borrell, Pauline Bass, Leon Worth

##### Infection Prevention and Hospital Epidemiology Unit, Department of Infectious Diseases, Alfred Health, Melbourne, Australia

###### **Correspondence:** Sue Borrell (S.Borrell@alfred.org.au)

**Background**

Carbapenemase-producing Enterobacteriaceae (CPE) infections are an emerging threat in specific regions of South East Asia, Europe, and the United States. CPE occurs sporadically in Australia with isolated genomic clusters. Different classes of carbapenemases are prevalent by region worldwide, and in Australia, imipenemase-producing CPE (bla IMP-4) is endemic at low levels. In our healthcare facility, carbapenemases other than IMP demonstrate epidemiological links to recent overseas healthcare or residency. Following retrospective recognition of a cluster of CPE cases linked to a single healthcare facility in Melbourne, the local health authority, the Victorian Department of Health and Human Services, released comprehensive consensus guidelines for the management of CPE in December 2015.

**Materials and methods**

Based on a “Search and Destroy” strategy, instruction is provided regarding identification of CPE, centralised reporting, contact tracing and screening, the need for alerts, clearance protocols for contacts, cleaning validation and 6-monthly point-prevalence surveys. Oversight of outbreaks is provided by a state incident management team and a single state reference laboratory performs genomic analysis of all isolates.

**Results**

This presentation will outline the experience and lessons learned in implementing these CPE guidelines in our healthcare facility. Examples include the development of new systems to identify and communicate CPE cases, electronic alerts for isolation and screening, a CPE staff education program and methods for conducting point-prevalence surveys in high-risk wards.

**Conclusions**

Early identification and screening of patients hospitalised abroad and the need for a functional IT platform to facilitate electronic flagging/alerts represent challenges likely to be faced by many healthcare facilities in our region.

## Session: Emerging Infectious Diseases

### EID2

**Withdrawn**

## Session: Environmental control

### E1 Analysis of risk factors among 51 measles patients infected with multiple drug-resistant bacteria

#### Zhao Xian-li, Li Xiao-long, Yao Xue-hua, Ren Wei, Zhang Xia Zeng

##### Gai-huan Henan Provincial Infectious Disease Hospital, Zhengzhou, China

###### **Correspondence:** Zhao Xian-li (zzlygrb@163.com)

**Background**

In recent years, we found rising in trends of measles infections, even in a small local epidemic. It may because of the measles virus’ genes per se, antigenic variation or other factors. Infected with measles virus can cause temporarily decline in human’s immune, especially cellular immunity. Lack of effective immune reactions can lead to secondary bacterial and multiple infections. We investigated measles-infected patients and evaluated susceptible factors for multiple drug-resistant bacteria infections. We also examined hospital infection prevention and control protocol in controlling measles outbreaks in our hospital.

**Materials and methods**

During 2013 to July 2015, 492 cases of patients with measles were detected in our hospital. We retrospectively reviewed and analyzed 51 measles-infected cases who had multiple drug-resistant bacteria infection data.

**Results**

We found that the main risk factors to be infected with multiple drug-resistant bacteria included: 1) age under 8 months, 2) abnormal cardiac functions, 3) having malnutrition and 4) having encephalitis.

**Conclusions**

The hospital should focus on patients under 8 months of age, with abnormal cardiac functions, having malnutrition or encephalitis to avoid multiple drug resistant bacteria infections and decrease mortality by strengthening treatment and implementation of infection prevention and control protocols.

## Session: Environmental control

### E2 A local experience sharing: hand wash basin as a potential source of carbapenemase-producing Enterobacteriaceae transmission in hospital environments

#### Man Ying Kong, Christopher Koon Chi Lai, Suet Yi Lee, Ngai Chong Tsang

##### Queen Elizabeth Hospital, Hong Kong, China

###### **Correspondence:** Man Ying Kong (kongmy@ha.org.hk)

**Background**

The global spread of Carbapenemase-producing *Enterobacteriaceae* (CPE) is a major challenge for infection control practitioners.We adopted a proactive approach that all CPE carriers were isolated in a designated ward with strict contact precautions. Here, we investigated if CPE can survive terminal disinfection in hospital environment. Our study aimed to evaluate the extent of CPE contamination in patient care environment after terminal disinfection using microbiological sampling.

**Materials and methods**

Microbiological samples for CPE were taken from the general wards’ environment whenever a patient newly identified with CPE was removed for isolation. Environmental samplings were collected by trained personnel. High-touch and wet surfaces were sampled using sterile Polywipe sponge. ChromID CARBA agar was used for selective cultivation of CPE. Suspicious colonies grown after overnight incubation at 35 °C were further examined for carbapenemase production using CARBA-NP. We confirmed carbapenemase production using GeneXpert CARBA.

**Results**

Between 7 April 2014 and 4 October 2016, 468 environmental samples were collected from 23 wards. We found 1.92%tested positive for CPE that included7 hand-wash basins, one sink and one hospital curtain. These isolates were IMP-producing CPE. Six affected basins/sinks were cleared from CPE after cleaned with detergent followed by disinfection with 5.25% sodium hypochlorite solution (1,000 ppm) daily for one week. The CPE in the remaining two hand-wash basins survived for 21 days after daily decontamination and the basins need to be replaced.

**Conclusions**

Our results highlighted hand-wash basins may serve as a potential environmental reservoir for CPE. As standardized decontamination regimen for sinks was lacking, we recommended hand-wash basins should not be used for the disposal of body fluids.

## Session: Environmental control

### E3 Effectiveness of decontamination of isolation wards using the spectra 1000 ultra-violet c light system

#### MM O’Donoghue^1^, MV Boost^1^, LKP Suen^1^, GK Siu^2^, KW Mui^3^, CKC Lai^4^, DNC Tsang^4^

##### ^1^Squina International Centre for Infection Control, School of Nursing, The Hong Kong Polytechnic University (PolyU),Hong Kong, China; ^2^Department of Health Technology and Informatics, The Hong Kong Polytechnic University (PolyU),Hong Kong, China; ^3^Department of Building Services Engineering, The Hong Kong Polytechnic University (PolyU),Hong Kong, China; ^4^Queen Elizabeth Hospital,Hong Kong, China

###### **Correspondence:** MM O'Donoghue (margaret.o.donoghue@polyu.edu.hk)

**Background**

Terminal cleaning of isolation rooms is an essential step in infection control. However, traditional cleaning and disinfection may be inadequately performed by time limitation. Ultra-violet (UV) devices are effective in environmental decontamination but their cost can be prohibitive. Recently, a more economical version of a UV-C sterilizing unit has become available but its effectiveness for environmental decontamination has not yet been independently evaluated. Our study aimed to evaluate the effectiveness of the Spectra 1000 UVC light system to reduce viability of healthcare-associated pathogens in a ward environment.

**Materials and methods**

Four organisms (*Staphylococcus aureus*, *Enterococcus faecalis, Acinetobacter baumannii* and *Klebsiella pneumoniae)* were coated onto designated areas of formica that were then attached to eight locations in the room. The room was irradiated for 15 minutes and the lamp was moved to a second position then treatment was repeated. Cultures were performed and resulting colonies were enumerated. Surviving numbers were compared with non-irradiated controls.

**Results**

All organisms were rendered non-viable in areas receiving direct irradiation or substantial reflected light. At two sites where heavily shaded (rear of bedside lockers and armchairs), a 2-log reduction in viability of *E. faecalis* was observed.

**Conclusions**

Spectra 1000 UVC light offered an effective adjunct to conventional terminal disinfection of isolation rooms. Although two irradiation periods using two positions of the lamp were needed, to ensure that shaded areas would receive adequate treatment, the total time for disinfection was only 30 minutes. As UV treatment does not produce residues, there is no down time after use.

## Session: Environmental control

### E4 The current status of cross-infection risks in hotels

#### Yuka Sato, Mariko Tateishi, Mutsuko Mihashi

##### Kurume University, Kurume, Fukuoka, Japan

###### **Correspondence:** Yuka Sato (yukasato503@gmail.com)

**Background**

In Japan, the Ministry of Health, Labour, and Welfare published the “Guidelines for the Prevention of New Strains of Influenza Infections in Employers and Employees [1]”. However, our survey, which investigated infection control for new strains of influenza in 2014, revealed that recognition of these guidelines was low, showing that infection prevention control had been insufficiently implemented in hotels. Our study aimed to identify the current status of cross-infection risks in hotels, and use the results to improvethe control of infectious diseases in such areas.

**Materials and methods**

The study ran from March 17-18, 2016. The study volunteers were members of the All Japan *Ryokan* Hotel Association. To assess environmental contamination levels in hotel settings, the ATP and AMP swab test kits (Kikkoman Lumitester PD-20) were used. Frequently touched surfaces were measured for contamination. Measurement sites included 30 that were measured intermittently, and 39 that were measured before and after cleaning.

**Results**

There were 5 intermittently measured monitoring sites that exceeded 5,000 RLU including the open/close buttons inside the kitchen elevator and first floor handrails. There were 5 monitoring sites measured before and after cleaning that exceeded 5,000 RLU even after cleaning, including the inner sides of restaurants’ sliding doors and the inner washroom door knobs of guest rooms.

**Conclusions**

ATP values of more than 5,000 RLU were detected at some monitoring sites, suggesting the need to reconsider methods and frequency of cleaning by taking risk of cross-infection into account.

**References**

1. Ministry of Health, Labour, and Welfare websate. Guidelines for the Prevention of New Strains of Influenza Infections in Employers and Employees (update 2009 Feb 17); http://www.mhlw.go.jp/bunya/kenkou/kekkaku-kansenshou04/pdf/090217keikaku-08.pdf (cited 2014 Oct 25)

## Session: Environmental control

### E5 Utilization of infection control risk assessment tool in improving the compliance to infection prevention and control during construction activities in the hospital

#### Jose Paulo Flor, Marko Bautista, V Jay De Roxas, Justine Vergara, Nicolo Andrei Añonuevo, Marion Kwek, Jose Acuin, Anna Josea Sanchez, Avel Bathan

##### Asian Hospital and Medical Center, Muntinlupa, National Capital Region, Philippines

###### **Correspondence:** Nicolo Andrei Añonuevo (naanonuevo@asianhospital.com)

**Background**

The purpose of this study was to test the effectively of the Infection Control Risk Assessment (ICRA) monitoring tool developed by the Infection Prevention and Control Unit (IPCU) of Asian Hospital and Medical Center with the aim to increase the compliance of construction workers to recommended infection prevention and control measures during construction, renovation and demolition in the hospital.

**Materials and methods**

Indicated in the ICRA monitoring tool were the details of the activity and the infection risk level (Class I,II,III and IV). The design used wasa quasi-experimental designwhich was conducted among all construction projects in the hospital within a 1-year period. The percent compliance was computed by number of compliant projects per month over total number of monthly projects which thenmultiplied by 100.

**Results**

There were a total of 151 construction projects monitored by direct observation which utilized the ICRA tool. Other interventions included orientation of construction workers to the tool, acknowledgment and accountability of recommended infection prevention and control measures by signing the tool and lastly, making use of the tool to provide feedback. Results show an improvement in the compliance to infection prevention and control interventions from average of 84% during pre-intervention to 91% post intervention.

**Conclusions**

Having an ICRA tool paved the way for construction workers to be pro-active and be involved in preventing infections brought by construction, renovation and demolition.

## Session: Environmental control

### E6 Garnering staff towards a cleaner and safer environment: the national kidney foundation experience

#### Jamilah Binte Jantan^1^, Chua Chor Guek^1^, Eu Chiow Kian^1^, Pampe Anak Pirido^1^, Nur Fadilah Binte Mohd Aron^2^, Leah May Estacio^3^, Francis Alvarez Palana^4^, Michelle Gracia^5^, Nur Syafiqah Binte Shamsuddin^6^, Kersten Timbad Castro^7^, Madonna Baloria^8^, Faezah Binte Adam^9^

##### ^1^National kidney foundation, Singapore, Singapore; ^2^Jurong West1 Dialysis Centre, Singapore, Singapore; ^3^Kolam Ayer Dialysis Centre, Singapore, Singapore; ^4^Woodlands1 Dialysis Centre, Singapore, Singapore; ^5^Teck Whye Dialysis Centre, Singapore, Singapore; ^6^Yishun1 Dialysis Centre, Singapore, Singapore; ^7^Hougang1 Dialysis Centre, Singapore, Singapore; ^8^Kim Keat Dialysis Centre, Singapore, Singapore; ^9^Tampines2 Dialysis Centre, Singapore, Singapore

###### **Correspondence:** Jamilah Binte Jantan (jamilah.jantan@nkfs.org)

**Background**

In the dialysis centre, there is potential for cross transmission of infectious agents through contaminated devices, hands, equipment, supplies and environmental surface during haemodialysis (HD) treatment. To reduce the risk of acquiring infections, staff routinely clean and disinfect medical equipment and high-touch areas after each patient's HD treatment at the National Kidney Foundation (NKF). This study aim of the study is to assess environmental cleaning of high-touch areas and develop intervention program to achieve compliance ≥85%.

**Materials and methods**

This is a quantitative study involving 29 Infection Control Link Nurses (ICLNs) at the Community-based Dialysis Centres (CB-DCs), NKF from October 2015 to April 2016. In November 2015, ICLNs conducted an environmental cleaning assessment of high-touch areas using a checklist and Glo Germ Kits, to ascertain the efficacy of environmental cleaning at 29 CB-DCs. Pre-study data showed an overall average of 67% compliance. RCA revealed the absence of an audit tool for high-touch areas, a lack of training leading to knowledge deficit, poor cleaning techniques and staff incompetency. Interventions included a checklist (audit tool) for environmental cleaning assessment of high-touch areas, a "Train the Trainer" programme for the 29 ICLNs, an annual competency assessment and video tutorials on environmental hygiene to standardise practice.

**Results**

Following the interventions, environmental cleaning assessment of high-touch areas showed an overall average of 86% compliance, with 17 CB-DCs achieving ≥85% compliance in environmental cleaning of high-touch areas.

**Conclusions**

This study illustrated that the intervention programme increased staff awareness, thereby improving compliance. Besides promoting positive outcomes, it enhanced the internal monitoring system at NKF.

## Session: Environmental control

### E7 Are surfaces of gym equipment a source of methicillin-resistant *Staphylococcus aureus* (MRSA) colonisation in the rehabilitation centre?

#### Zhang Wei^1^, Poh Bee Fong^2^, Marimuthu Kalisvar^3^, Angela Chow^4^, Brenda Ang^5^

##### ^1^Infection Control Unit, Tan Tock Seng Hospital, Singapore, Singapore; ^2^Infection Control Unit, Tan Tock Seng Hospital, Singapore, Singapore; ^3^Infectious Disease, Tan Tock Seng Hospital, Singapore, Singapore; ^4^Clinical Epidemiology, Tan Tock Seng Hospital, Singapore, Singapore; ^5^Infectious Disease, Tan Tock Seng Hospital, Singapore, Singapore

###### **Correspondence:** Zhang Wei (zhang_wei@ttsh.com.sg)

**Background**

In March 2016, a surge of 34 MRSA acquisitions (30 from screening and 4 from urine cultures) was noted in rehabilitation ward (REH-ward) at Tan Tock Seng Hospital, Singapore. Our objective was to investigate if fomite transmission could be a cause of these acquisitions. We conducted one-day surveillance screening of the gym equipment and REH-ward’s environment.

**Materials and methods**

Samples were collected by rolling swabs moistened with sterile saline five times on the surfaces of gym equipment before and after use. In the ward, selected patients’ beds and common items or equipment in shared area were also sampled. Samples were cultured for MRSA using selective chromogenic media.

**Results**

In the gym, all 156 samples collected from equipment pre-use were negative for MRSA. However, 7.3% (6/85) of the samples collected after use were MRSA positive. In the wards, all swabs (55) that were taken from the common shared area such as computers, case notes carts, were negative. Two out of 12 beds (16.7%) occupied by MRSA carriers and 3 out of 53 beds (5.7%) occupied by non-MRSA carriers were contaminated with MRSA (OR 3.33, 95%CI 0.24- 32.46, *p* = 0.23). Overall rates of MRSA-positive swabs were comparable between the wards and gym (2.5% vs. 2.2%, *p* = 0.82).

**Conclusions**

Gym equipment was not more likely than the ward environment to contribute to MRSA acquisition. The importance of environmental cleaning in all areas including rehabilitation facilities cannot be over-emphasised.

## Session: Environmental control

### E8 Reforming program of environmental cleaning

#### I-Ju Chuang, Yi-ChunCho, Yu-Fen Chiu, Lung-Chih Chen, Yi-Chun Lin, Shao-Xing Dong, Yi-Chieh Lee, Hui-Chen Kuan, Hsin-Hua Lin, Chia-Chun Chi, Chin-Te Lu

##### Lo-Tung Pohai Hospital, Lo-Hsu Foundation, Incl, Ilan, Luodong Town, Taiwan

###### **Correspondence:** I-Ju Chuang (897077@mail.pohai.org.tw)

**Background**

With the widespread use of antibiotics in the treatment of human bacterial infections, the multidrug-resistant microorganisms also appear to threaten human health. Environmental cleaning to avoid the spread of bacteria and healthcare-associated infection is an important part of healthcare infection control practice. Through this program, our hospital aimed to improve the environmental cleaning, reduce the bacterial antibiotics resistance, and further reduce the use of antibiotics.

**Materials and methods**

We reformed program of environmental cleaning and measured incidence of multidrug-resistant bacteria and consumption of designated antibiotics.

**Results**

Our results were shown below. The unqualified rate in environmental cleanliness of our cleaners was 39.1% before this program was implemented and 21.7% after this program that demonstrated 44.5% reduction. The numbers of healthcare-associated infections with multidrug-resistant bacteria was 30 before this program, and 8 after this program (73% reduction). The consumption of anti-methicillin-resistant Staphylococcus aureus was 142.0 defined daily dose (DDD) /1000 bed-days before this program, and 101.5 DDD /1000 bed-days after this program (28.5% reduction). The consumption of glycopeptides was 120.0 DDD /1000 bed-days before this program, and 83.2 DDD /1000 bed-days after this program (30.8% reduction). The consumption of carbapenems was 164.5 DDD /1000 bed-days before this program, and 98.1 DDD /1000 bed-days after this program (40.4% reduction).

**Conclusions**

According to our results, environmental cleaning may effectively reduce the number of healthcare-associated infections with multidrug-resistant bacteria and used of broad-spectrum antibiotics. If it can be promoted consistently, in accordance to the qualified data, the use of antibiotics could be reduced and the prevention of bacterial resistance occurred.

## Session: Environmental control

### E9 To investigate the expiry date of antimicrobial curtain

#### Fu-chieh Chang, Chang-pan Liu

##### Infection control center, Mackay memorial hospital, Hsinchu, Taiwan

###### **Correspondence:** Fu-chieh Chang (maple0711@gmail.com)

**Background**

The invention related to an antibacterial treatment process of a curtain fabric material with functions of free washing and environmental protection. They were used for the antibacterial treatment of a non-woven fabric made from polypropylene fibers and as the curtain fabric material with functions of free washing and environmental protection. Before our hospital use this production, we aimed to investigate the expiry date of antimicrobial curtain to establish our protocol.

**Materials and methods**

We conducted this experiment from August to October 2016. In this study, we used three companies’ antimicrobial curtains. We used the *C.difficile*, MRSA and *A. baumannii* as study models. Then we putted the antimicrobial curtain on the agar then see the bacteria growth or not.

**Results**

According to our study, *C. difficile*, MRSA and *A. baumannii* were not detected in the antimicrobial curtains. After 11 weeks, there were no bacteria growths on the curtain.

**Conclusions**

The curtain fabric material produced by the invention has the advantages of good air permeability, easy maintenance of dry curtain fabric surfaces, dirt resistance, free washing, no toxicity and irritative peculiar smell, easy recycling, environmental protection, good antibacterial property and low antibacterial treatment cost.

## Session: Environmental control

### E10

**Withdrawn**

## Session: Environmental control

### E11 Evaluation of the appropriateness of the standard operating procedure and propose amendments to ensure of the cleaning/disinfection in Intensive Care Unit

#### Tang Ya-Fen^1,2^, Su Li-Hsiang^1^, Liu Jien-Wei^1,3,4^

##### ^1^Committee of Infection Control, Chang Gung University Medical College, Kaohsiung, Taiwan; ^2^Departments of Clinical Pathology, Chang Gung University Medical College, Kaohsiung, Taiwan; ^3^Division of Infectious Diseases, Chang Gung University Medical College, Kaohsiung, Taiwan; ^4^Internal Medicine, Kaohsiung Chang Gung Memorial Hospital, Kaohsiung, Taiwan

###### **Correspondence:** Tang Ya-Fen (yafen@cgmh.org.tw)

**Background**

Environmental contamination is the important source for bacterial spread causing hospital-acquired infections. Environmental cleaning with an established standard operation procedure (SOP) and cleaning staff’s strict adherence to the SOP are therefore extremely important. However, evaluation of the effects of environmental cleaning in general has not been fully reported in the literature. This study aim to elucidate the effects of environmental cleaning with the established SOP and by the well trained cleaners in Kaohsiung Chang Gung Memorial Hospital (KSCGMH). Environmental cleaning was based on the SOP which followed the principle of cleaning from higher locations to lower ones, and from the contaminate areas to the comparatively cleaner ones in rooms where patients were staying.

**Materials and methods**

Before and after daily environmental cleaning routine, environmental surfaces were swabbed for sampling specimens for bacterial culture and for bacterial count evaluation in case of culture positive.

**Results**

Data showed that before and after cleaning environmental, bacterial burdens in environmental surfaces of the bedroom were significantly reduced (p < 0.0001).

**Conclusions**

Indicating the environmental cleaning with the current cleaning SOP and by these well trained cleaners has been effective.

## Session: Environmental control

### E12 Hospital-wide environmental cleaning monitoring program reduces healthcare-associated infections related to multidrug-resistant organism

#### Hsuehlan Chao, PinRu ChangChien, WeiFang Chen, ChungHsu Lai

##### E-DA Hospital, Kaohsiung, Taiwan

###### **Correspondence:** Hsuehlan Chao (lan3428519@gmail.com)

**Background**

The evidence-based policies to clean hospital environment can reduce the colonization and infection of multidrug-resistant organisms (MDROs). The purpose of this study is to measure the effectiveness of environmental cleaning policies.

**Materials and methods**

(1) The effectiveness of policies was examined by adenosine triphosphate (ATP) and microbial cultures (MCs) before and after the implementation of policies at both the general wards (GWs) and the intensive care units(ICUs). (2)The study audited selected 20 points related to MDROs, including bed rails, bed button beds (the desktop corner), etc.(3)The standard values: ATP less than 250 RLU at ICU and less than 500 RLU at GW. Bacterial colonies count < 100 CFU.

**Results**

(1)20/49 ATPs (41%) had been detected over 500RUL before policies, but 28/67 (42%) after policies without statistical difference (p = 0.45) in GWs. However, in ICUs, before policies 40/81 ATPs (49%) over250 RLU and after policies 30/67 ATPs over 250 RLU, no statistical difference (p = 0.29). (2) 50% selected points were over 500RUL both before and after policies at GWs. Only 16.6% selected points were over 250RUL in ICUs. (3) MCs had statistically significant (p = 0.01) before and after policies at GWs, including Oxacillin-Resistant *Staphylococcus aureus* (ORSA), *Enterococcus faecium* (VRE), *Acinetobacter baumannii*, (XDR).But ICUs were not dirty over standard both before and after policies.

**Conclusions**

This survey helps us understand how much dirty and contamination in the environment. Especially, bed rails, button, isolation unit, car-related equipment, room telephone, curtains. Establishing good environmental policies are very important to prevent healthcare-associated infections. ICUs environment is cleaner than GWs in this study.

## Session: Hand hygiene

### H1 Promoting effective hand hygiene practices through implementing standard guidelines at the healthcare facilities in Bangladesh

#### Lutfe Ara, Syed Mohammad Niaz Mowla, Shaikh Mahmud Kamal Vashkar

##### Clinical Governance Unit, International Centre for Diarrhoeal Disease Research, Bangladesh (icddr, b), Dhaka, Bangladesh

###### **Correspondence:** Lutfe Ara (lutfeara@icddrb.org)

**Background**

Contaminated hands are the foremost source of spreading infections in healthcare facilities. Wellness and safety of patients and healthcare workers (HCWs) can be achieved by promoting best practices in infection control through education and advocacy. We aimed to develop effective hand hygiene (HH) practices among HCWs by improving their knowledge, attitude and practices through implementing standard HH guidelines.

**Materials and methods**

A year-long project was conducted at two hospitals of Bangladesh. This included a baseline survey, intervention by implementing standard HH guidelines through classroom and hands-on training, and a post intervention survey. Pretest-posttest was conducted with preformed questionnaire and observation checklist during pre and post intervention surveys. Total of 600 physicians and nurses were trained on standard HH practices.

**Results**

At the Institute of Child and Mother Health, rate of HH compliance before patient contact improved from 4% to45.32%(p < 0.0001) among physicians and 2.07% to 60% (p < 0.0001) among nurses. After patient contact, it increased from 4.8% to 50.36% (p < 0.0001) among physicians and 5.93% to 59.51% (p < 0.0001) among nurses. At General Hospital, Sirajgonj, rate of compliance before patient contact increased from 2.25% to 49.18% (p < 0.0001) among physicians and 3.10% to 53.57% (p < 0.0001) among nurses. After patient contact, it increased from 2.25% to 58.20% (p < 0.0001) among physicians and 5.31% to 60.71% (p < 0.0001) among nurses.

**Conclusions**

The project outcomes signify that implementing standard HH guidelines improves the knowledge, attitude and practices of the HCWs. The results emphasize the necessity of continuous education and advocacy in improving HH compliance to promote excellence in infection prevention and control.

## Session: Hand hygiene

### H2 The effective hand hygiene promotional measures in hospital: listen to your staff

#### Wai Fong Chan

##### The Hong Kong Polytechnic University, North Point, Hong Kong, China

###### **Correspondence:** Wai Fong Chan (wfchan@alumni.cuhk.net)

**Background**

The World Health Organization multi-modal intervention to increase hand hygiene compliance was adopted in a rehabilitation hospital since 2008. However, the effectiveness of the individual measure was unknown. This study was conducted for evaluating the effectiveness of hand hygiene promotion activities used in the hospital.

**Materials and methods**

A cross-sectional survey was applied in 2012. A 16-item self-administered questionnaire was adopted to collect the opinion on the effectiveness of measures previously used on hand hygiene promotion. Nurses and healthcare assistants working in the inpatient settings of hospital were invited to participate in the survey. Seven-point Likert-type scale from “extremely ineffective” to “extremely effective” was used for ratingindividual items. Rasch measurement was employed for data analysis by Winsteps version 3.92.1.

**Results**

One hundred and seventy-nine questionnaires were returned contributing 97.2% of the response rate. The categories in the rating scale were collapsed into 4-point scale before further analysis. Thirty-one misfitting persons and three misfitting items were removed after examination in quantitative and qualitative manners. No differential item functioning was found between subgroups. The final scale was considered as unidimensional with reliabilities ranged at 0.95-0.96 and 0.91-0.92for persons and items respectively. “Placing the alcohol-based handrub” was identified as the most effective measure on hand hygiene promotion and “Set up an annual target of hand hygiene compliance” was considered as the least effective.

**Conclusions**

The survey identified and located the effective measures on hand hygiene promotion. For a more efficient approach, the hospital may prioritize the most effective items in hand hygiene promotion.

## Session: Hand hygiene

### H3 Effective hand hygiene with individual hand microbial profiles through validating scores of bioluminescene assay with actual microbial load

#### Mabel Yin ChunYau^1^, Karen Kam LingChong^2^, Tze OnLi^1^, Rajwinder Kaur ^3^

##### ^1^School of Medical and Health Sciences Tung Wah College, Hong Kong, China; ^2^Dietetic Department, Matilda International Hospital, Hong Kong, China; ^3^Nursing Department, Matilda International Hospital, Hong Kong, China

###### **Correspondence:** Mabel Yin ChunYau (mabelyau@twc.edu.hk)

**Background**

Adenosine-Triphosphate (ATP) bioluminescence assay has been popularly adopted in clinical and catering industry due to its ease of use and immediate results. ATP bioluminescence assay picks up cellular discharged ATP, which can also be found on cellular debris or organic components that are not microbial in nature.Its measurement on animate objects can be misleading. This study was developed for the catering crew of a private hospital as a Hand Hygiene (HH) practice monitoring. Microbial viable count was used as a validating reference for the ATP Relative Light Units (RLUs) as a control measure of HH effectiveness. A set of basal microbial values was developed for each staff member. This provided a convenient but reliable protocol for ATP luminometry users.

**Materials and methods**

Swab sampling was collected from crews’ hand for bacterial culture. Selective media and serological tests were used for pathogen screenings, which included *Staphylococcus aureus*, coliform and salmonella. Standard curves to demonstrate the correlation between the viable microbial counts and the corresponding RLUs of the ATP measurements were developed.

**Results**

The study showed the actual viable microbial density of individuals after handwashing did not correlate positively with RLUs. Each individual had his/her own confidence regarding to the limitation of RLUs. However, the HH compliance could be reflected by the viable microbial counts.Individual skin condition played a role in this association.

**Conclusions**

The measurement instilled a positive effect on the crew. Hand hygiene compliance can be reflected with the bench marking standard technique. Hand hygiene compliance was increased and microbial load was significantly reduced.

## Session: Hand hygiene

### H4 I pledge on hand hygiene: hand hygiene campaign in Caritas Medical Centre (CMC)

#### Ng Po Yan (carolpoyan@gmail.com)

##### Infection Control Team, Caritas Medical Centre, Hospital Authority, Hong Kong, China

**Background**

Hand hygiene is the single and most effective way to prevent the spread of microorganisms in hospital. When health care workers (HCWs) have own sense and aware of the importance of hand hygiene, it yields twice the result with half the effort in infection control. The study aimed to promulgate hand hygiene is the responsibility of every HCW and maintain hand hygiene as the standard of care in the daily work of HCWs

**Materials and methods**

On the International Hand Hygiene Day 5th May, a hand hygiene campaign was held in CMC. HCWs were invited to take photo and pledged on hand hygiene compliance. Hand hygiene technique was also taught personally by infection control nurses and return demonstration was needed during the activity. An instant photo was taken when they pledged. These photos were shown on a board during the hand hygiene promotion activity and returned to each colleague as a souvenir afterward.

**Results**

Hospital managers, frontline doctors and nurses, allied health professionals and supporting staff, total over 100 colleagues in CMC were pledged on hand hygiene on that day. This pledge motivated other colleagues to compile in hand hygiene. The hand hygiene compliance rate in CMC maintained over 90%.

**Conclusions**

Motivate health care workers to perform hand hygiene by a soft commitment is another way to promote and raise their awareness of hand hygiene.

## Session: Hand hygiene

### H5 Hand hygiene: dreams come true “clean care is safer care”

#### Gloria Chor Shan Chiu^1^, Christina WY Cheung^2^, Patricia TY Ching^1^, Radley HC Ching^1^, Conita HS Lam^3^, CH Kan^2^, Shirley SY Lee^1^, CP Chen^1^, Regina FY Chan^1^, Annie FY Leung^1^, Isadora LC Wong^1^, S S Lam^1^, Queenie WL Chan^4^

##### ^1^Hospital Authority, Hong Kong, China; ^2^Baptist Hospital, Hong Kong, China; ^3^HKICNA, Hong Kong, China; ^4^HK Sanatorium & Hospital, Hong Kong, China

###### **Correspondence:** Gloria Chor Shan Chiu (gloriacatcat2@yahoo.com.hk)

**Background**

More than hundreds of millions of worldwide people are suffered from infection regardless of acquiring in the Community or Healthcare setting. Most of the infections especially the Healthcare associated Infections (HCAIs) are preventable through adherence to patient care practices. Hand hygiene (HH) is the most effective and easier way to prevent and control the infections in which also being the major component in WHO the First Global Patient Safety Challenge.

**Materials and methods**

To introduce the “WHO hand hygiene save life campaign” and enhance the awareness of public and healthcare workers for the importance of hand hygiene. Hong Kong Infection Control Nurse Association (HKICNA) has joined actively in the community events such as “World Health Day Carnival” since 2008. In these few years, there were over a thousand public to participate in HH and infection control related educational game booths and talks.

**Results**

Further to extend the engagement of public and healthcare workers, a poster design competition for promotion of Hand Hygiene was organized in 2012; the winning poster was used as “Talking Wall” in community and healthcare settings and the background of HKICNA’s souvenirs.

In 2014, a creative reminder- hand-held electric fan with visual lit up “Hand Hygiene" was distributed.

In addition, another innovative idea – two HH Dances were designed to continuously promote the HH; they stress that HH practice should start from children to adulthood, from healthcare worker to all in the community. Both of them were used as a tool for promoting in hospitals and schools and assessable in YouTube which was gained thousands of 'Likes'

**Conclusions**

HKICNA is working hard on introducing hand hygiene concept for infections prevention and control in healthcare settings and community. The concept of "Clean Hand Save Lives" will continue be emphasized.

## Session: Hand hygiene

### H6 Unforgettable 7 steps & 5 moments” – critique on staff’s technique and accuracy

#### Cecilia Chan, Rajwinder Kaur

##### Matilda International Hospital, Hong Kong, China

###### **Correspondence:** Cecilia Chan (cecilia@matilda.org)

**Background**

Hand hygiene (HH) is recognized as the most effective measure to prevent the spread of micro-organisms through hand contact during patient care. Besides yearly mandatory infection control training through On-line Learning Management System, Matilda International Hospital (MIH) also provides relentless training to staff emphasizing the importance of proper hand hygiene and embracing WHO 5 moments. In June 2016, educational booth game – “Unforgettable 5 moments and 7 steps” was held to assess knowledge and techniques of both clinical and non-clinical. This study aimed to evaluate the techniques of staff’s hand hygiene practice, compliance to rubbing time and accuracy in reiterating the 5 moments.

**Materials and methods**

Observational methods were used to evaluate HH technique and rubbing time. Staff were required to accurately call to remembrance the 5 moments through direct questioning and demonstrate 7 steps of hand hygiene technique with at least 20 seconds of rubbing time. Immediate feedbacks were supplemented.

**Results**

The overall accuracy of all assessed criteria’s was 90.53%. Majority of HH steps achieved greater than 90% compliance rate except “Between finger” and “Back of finger”. “Before clean aseptic procedure” demonstrated to be the most difficult to recall.

**Conclusions**

The audit allowed for gaps and in-depth understanding of staff HH practices to be more accurately identified with subsequent staff training strategies to be drawn and implemented.

## Session: Hand hygiene

### H7 Knowledge and self-reported hand hygiene compliance among nurses in Shiraz, Iran, 2016

#### Seyed Sadeq Seyed Nematian^1^, Charles John Palenik^2^, Mehrdad Askarian^1^

##### ^1^Shiraz University of Medical Sciences, Shiraz, Iran; ^2^Indiana University School of Dentistry, Indianapolis, IN, USA

###### **Correspondence:** Mehrdad Askarian (askariam@sums.ac.ir)

**Background**

Alcohol-based hand rubs (ABHRs) are the preferred methods for performing routine hand hygiene (HH) in healthcare facilities. However, soap-and-water hand washing is still popular. This study measured the HH knowledge and self-reported practices of Shiraz Nemazee Hospital nurses.

**Materials and methods**

This study employed two questionnaires. A six-question survey covered HH knowledge, (19 possible points), while HH practices were monitored in a second survey containing four multi-part self-reporting inquiries (37 possible points) in 2016. Surveys were voluntarily completed at work. Responses were analyzed anonymously.

**Results**

342 nurses completed the questionnaire.54.4%had formal HH training in the past year.55.6%reported using ABHRs for more than a year. 53.8% preferred traditional soap-and-water hand washing. Eleven nurses never used ABHRs. Nursing experience varied - 15.1% (>ten years), 10.8% (5-10 years), 35.2% (2-5 years) and 28.9% (<2 years). Knowledge scores ranged from 11-15 (high score was17). Self-reported HH compliance scores ranged from 24-31 (high score of 37). A positive, but weak correlation existed between knowledge and self-reported practice scores (r =0.28, p <0.001). No correlation existed between years of experience and knowledge (p =0.85, r = 0.011) or self –reported practice scores (p = 0.86, r = 0.01). Also, no correlation was found between age and self-reported practices (p =0.4, r = - 0.048) and/or knowledge scores (p =0.85, r = -0.011).

**Conclusions**

HH training needs to be increased for all nurses. Stressing benefits of ABHR use could improve HH compliance and effectiveness.

## Session: Hand hygiene

### H8 Observing hand hygiene compliance among hospital nurses in southern Iran, 2016

#### Seyed Sadeq Seyed Nematian^1^, Charles John Palenik^2^,Nahid Hatam^1^, Mehrdad Askarian^1^

##### ^1^Shiraz University of Medical Sciences, Shiraz, Iran; ^2^Indiana University School of Dentistry, Indianapolis, IN, USA

###### **Correspondence:** Nahid Hatam (hatamn@sums.ac.ir)

**Background**

Hand hygiene (HH) is the most effective way to prevent healthcare-associated infections. Unfortunately, HH compliance worldwide is suboptimal. In this study, we measured HH compliance among Shiraz Nemazee Hospital nurses.

**Materials and methods**

A WHO method for direct observation of HH was used. We observed staff members (nurses, paramedics and auxiliaries) HH practices. A single trained observer made all determinations. Nurses were not informed of being observed. The study was conducted in 29 hospitals wards involving four major services (intensive care, internal medicine, surgery and pediatrics) during January through June 2016.

**Results**

1097 observations were made. The overall HH compliance rate was 39.23% with greatest compliance (44.12%) occurring in intensive care wards, while internal disease ward shad the lowest (26.88%) rates. HH compliance in surgical and pediatrics wards was 39.15% and 38.30%.HH compliance between intensive care and internal disease wards was significantly (p < 0.001) different. However, no significant differences were noted between intensive care and surgical wards (p = 0.16) and intensive care and pediatric wards (p = 0.24). Also, there was no statistically significance difference between professional nursing categories (p = 0.66). Alcohol- based hand rubs were used during 81% of HH events. Soap-and-water hand washing was used the remainder of times.

**Conclusions**

Type of nursing position was not a reliable predictor of higher HH compliance. HH policies in intensive care wards appear more effective than those of other wards. Alcohol-based hand rubs proved to be the preferred HH methods.

## Session: Hand hygiene

### H9 Scenario-based simulation healthcare education for hand hygiene

#### Itaru Nakamura^1^, Hiroaki Fujita^1,2^, Ayaka Tsukimori^1,2^,Takehito Kobayashi^1,2^, Akihiro Sato^1^, Shinji Fukushima^1^, Tetsuya Matsumoto^2^

##### ^1^Department of Infection Prevention and Control, Tokyo Medical University Hospital, Tokyo, Japan; ^2^Department of Microbiology, Tokyo Medical University, Tokyo, Japan

###### **Correspondence:** Itaru Nakamura (task300@tokyo-med.ac.jp)

**Background**

Simulation healthcare education is widely used in medical education and has great potential. However, scenario-based simulation healthcare education for preventing nosocomial infections has not been described. This study aimed to determine the effectiveness of scenario-based simulation education to improve hand hygiene.

**Materials and methods**

A single-centre, prospective, cohort study was conducted at Tokyo Medical University Hospital (1015 beds), an acute-care teaching hospital, from January 2011 to December 2014. Each infection-control training course (ICTC) was held every month and lasted 2 hours. Trainees put on and removed personal protective equipment under scenarios of standard precaution (two scenarios) and contact precaution with Methicillin-resistant *Staphylococcus aureus* (one scenario), while considering timing of hand hygiene. We determined the correlations between the participation rate in the simulation education and use of alcohol-based hand disinfection and reduction of catheter-related blood stream infection (CRBSI).

**Results**

There were 1077 trainees. The total participation rate for hospital staff was 76% by the end of the study. The overall correlation between use of alcohol-based hand disinfection in the hospital and the course participation rate was significant (correlation coefficient, 0.97). An inverse correlation (−0.94) was observed for the relation between the ICTC participation rate and the incidence of CRBSI. With participation in the ICTC, CRBSIs due to *Staphylococcus* spp. and *Enterobacteriaceae* were significantly lower than those due to *Candida* spp.

**Conclusions**

Our ICTC had a positive effect on hand hygiene and reducing CRBSI. This study is the first effective scenario-based simulation healthcare education to hand hygiene and control of nosocomial infection.

## Session: Hand hygiene

### H10 Increasing hand hygiene compliance through an evidenced-based strategy

#### Jose Paulo Flor, Nicolo Andrei Añonuevo, Marko Bautista, Justine Vergara, V James De Roxas, Marion Kwek

##### Asian Hospital and Medical Center, Muntinlupa, National Capital Region, Philippines

###### **Correspondence:** Nicolo Andrei Añonuevo (naanonuevo@asianhospital.com)

**Background**

The purpose of this study is to increase and sustain hand hygiene compliance through evidence-based approach and to relate compliance with the trend of healthcare associated infections in the hospital.

**Materials and methods**

The study was conducted over a 1 year period from September 2015 to August 2016. The methods used in monitoring hand hygiene compliance are “Direct Observation” which includes self-reporting and by the use of secret shoppers. Another is “Electronic Monitoring” through the use of Radio Frequency Identification Device. Surveillance of healthcare associated infection (HAI) is conducted in the General Nursing Units, Telemetry and Intensive Care Units. Interventions to increase hand hygiene compliance were implemented such as Training of nurse LINC representatives in monitoring hand hygiene compliance, use of social media (Facebook) in promoting hand hygiene, recognition of individuals and department with high compliance.

**Results**

After the implementation of interventions, result showed an increase in the hand hygiene compliance from below 50%, during the start of the period monitored, to above 80% during the succeeding months. Comparison of hand hygiene compliance versus healthcare associated infection rates was shown through a graph, this information was cascaded to the different departments during unit meetings. Correlation showed a contrasting trend between Hand hygiene compliance and healthcare associated infection rates.

**Conclusions**

It was therefore concluded that an increase in the compliance to hand hygiene can decrease the healthcare associated infections among patients. In addition, feedback methods and other evidenced-based interventions can increase hand hygiene compliance.

## Session: Hand hygiene

### H11 Implementing multiple interventions through the use of an electronic monitoring system in increasing the hand hygiene compliance in medical surgical ICU

#### Jose Paulo Flor, Marko Bautista, Justine Vergara, V James De Roxas, Nicolo AndreiAñonuevo, Marion Kwek

##### Asian Hospital and Medical Center, Muntinlupa, National Capital Region, Philippines

###### **Correspondence:** Nicolo AndreiAñonuevo (naanonuevo@asianhospital.com)

**Background**

There is no single solution in addressing poor compliance to hand hygiene. The purpose of this study is to increase the compliance of hand hygiene in the Medical Surgical Intensive Care Unit (MSICU) by the use of multiple interventions and the aid of an electronic monitoring system. The Infection Prevention and Control Unit (IPCU) would also like to correlate the trend of hand hygiene rate with healthcare associated infections in the MSICU.

**Materials and methods**

The data collected is generated by an automated system through the use of radio frequency badges worn by the healthcare worker and sensors attached to the hand rubs and soap dispensers. Badges provide real time feedback by means of an alarm system. A quasi-experimental design was used to test the effectiveness of the interventions implemented. The utilization of visual boards in providing feedback, converting the door entrance into a giant hand hygiene poster, use of social media, and reward system were included in the interventions.

**Results**

Total number of opportunities captured is 121,252. Post intervention data showed an increase in the compliance from below 50% to above 80%.

**Conclusions**

It was therefore concluded that the multiple strategic approach, through the use of electronic monitoring, helped in increasing compliance of healthcare workers to hand hygiene. Moreover, when hand hygiene trend was compared to the healthcare associated infection, a contrasting trend was evident.

## Session: Hand hygiene

### H12 Hand hygiene compliance audits – is there a role for the ‘link doctor’?

#### Yeng May Ho, Jia Qi Kum, Bee Fong Poh, Kalisvar Marimuthu, Brenda Ang

##### Tan Tock Seng Hospital, Singapore, Singapore

###### **Correspondence:** Brenda Ang (Brenda_Ang@ttsh.com.sg)

**Background**

Auditing of Hand Hygiene compliance is usually done by the Infection Control (IC) team. Because it is time consuming and resource intensive, hospitals often rely on ‘link nurses’ to do audits. There are questions as to how these results would correspond to that done by other observers who do not belong to the same unit. As part of a hospital-wide Hand Hygiene program, an initiative to involve young doctors in Hand Hygiene activities was mooted and residents nominated by heads of departments to be hand hygiene auditors.

**Materials and methods**

These residents (‘link doctors’) were first trained by the Infection Control nurses, following WHO Five Moments, and underwent inter-rater reliability testing. They were tasked with doing audits on Hand Hygiene compliance for their own departments, at times of their choosing. The study period was from February to Sep 2015. The results of their audits were submitted to the Infection Control Unit, and compared with the results done by the IC unit.

**Results**

While the overall compliance rate when audits were done by the ‘link doctors’ was 76%, it was 46% when done by IC team. The only ‘moment’ where there was no difference in compliance rates between them was ‘after patient contact’.

**Conclusions**

There was significant difference in hand hygiene compliance rates when audits were done by ‘link doctors’. While there might be concerns about the validity of their results, such exercises could lead to improved understanding, ownership of, and participation in activities to improve hand hygiene.

## Session: Hand hygiene

### H13 A workshop for training hand hygiene observers and evaluating learning outcomes

#### Tzu-Yin Liu, Sin-Man Chu, Hui-Zhu Chen, Tun-chieh Chen

##### Kaohsiung Municipal Ta-Tung Hospital, Kaohsiung,Cianjin District, Taiwan

###### **Correspondence:** Tzu-Yin Liu (ned740206@yahoo.com.tw)

**Background**

To achieve good compliance and correct hand hygiene for healthcare workers in clinical setting, we conducted quality audit by non-fixed observers to prevent the Hawthorne effect.This study aimed to train hand hygiene observers who can educate hand hygiene audit ability and reinforce the concepts of good compliance of correct hand hygiene

**Materials and methods**

The process included 1.Set up the standard of hand hygiene audit; 2. Hold the lecture for the perception of the importance of hand hygiene; 3. Correct hand hygiene hands-on practice: using fluorescent cream on hands and washing hands with water and soap and then checked hands under fluorescent lamp; 4. Teaching hand hygiene audit in real clinical settings; 5. Hand hygiene Q &A, sharing and discussion.

**Results**

The workshop trained 23 nurses as hand hygiene observers and all passed the assessment. The average learning outcome evaluation is 86.3%. The average satisfactory rate of the workshop is 98.3% via the 5 domains of the value of learning, applicability, enhancing professional knowledge, the appropriate application of educational materials, the achievement of learning expectation. All trained hand hygiene observers can apply the concepts in clinical settings.

**Conclusions**

The workshop composed with hands-on practice, teaching in real clinical settings and sharing-discussion instead of one-way lecturing. This multimodal educational program integrated with lecturing, idea sharing, experiments and teach-reply methods to enhance learning outcomes and reinforce the hand hygiene perception. Through the educational program, we transferred the hand hygiene knowledge to modify healthcare workers’ perception and then their quality improvement attitude to cooperate the patient safety strategies in the whole institute.

## Session: Hand hygiene

### H14 Evaluation direct observation methods to measure compliance with hand hygiene rates in regional hospital

#### Yichun Chen (clh30004@mail.chimei.org.tw)

##### Department of Nursing, Chi Mei Medical Center, Liouying, Tainan, Taiwan

**Background**

Compliance with hand hygiene is the most important concept in preventing infection to patients in health care settings. The World Health Organization (WHO) guidelines on hand hygiene in health care indicate that the direct observation methods to measure compliance with hand hygiene are the gold standard, also the most reliable methods of measurement.The study aimed to explore the compliance with hand hygiene rates in regional hospital.

**Materials and methods**

Direct observation methods were performed to measure compliance with hand hygiene in physicians, nurses, nurse practitioners and nurse assistants from January 1, 2016 to September 30, 2016 of a regional hospital in Taiwan.

**Results**

Compliance with hand hygiene rates 87.37% (83/95), physicians 80% (8/10), nurses 92.5% (62/67), nurse practitioners 76.9% (10/13) and nurse assistants 80% (4/5).

**Conclusions**

Compliance with hand hygiene rates were higher than reported in the literature (CDC). Another problem with direct observation was the Hawthorne effect produced by the observation. When a medical practitioner discovered that he or she was observed, he or she would be conscious of his or her own observation and deliberately changed his or her behavior to increase the frequency of hand hygiene. In the pursuit of the desired positive results, and affect the hand hygiene compliance measurement results. Recommendation to regular change auditor or monitoring type, ex: video recorder, but given these measurement limitations, more valid, practical, and less costly methods are needed.

## Session: Hand hygiene

### H15 The efficiency of patients and visitors’ education for promoting handwashing and respiratory hygiene compliance in a local community hospital

#### Ya-Ching Tsao (supersunshine2011@gmail.com)

##### Infection control office, Longtan Min-Sheng hospital, Longtan, Taoyuan, Taiwan

**Background**

Hand washing and respiratory hygiene are fundamental in infection control management. They seem a global language in health settings. A majority studies investigate the relationship between hand hygiene and infectious diseases transmission among healthcare staffs. The aim of this survey was to discover the efficiency of hand hygiene and cough manners education model among patients and visitors.

**Materials and methods**

This study measured by questionnaires with hand hygiene and cough manner observation compliance tools. Duration of data collection was from July, 2015 to August, 2016. 428 patients and visitors of a local community hospital in North Taiwan were submitted their questionnaire.

**Results**

83.41% participants were over 61 years old (age group: 57.24% 61-64 years old, 26.17%over 65 years old). The finding from cough manner observation revealed that only 33.04% participants allowed cough manners while they have sneezing, coughing or flu symptoms. This had significant difference to the same question of questionnaire (82.24%). The difference also appeared in hand hygiene observation, 59.65% observers performed hand washing in their hand hygiene opportunities which was considerably lower than the questionnaire result (98.83%). These findings investigate the attitudes toward hand washing and respiratory hygiene compliance among patients and visitors. Thus, a patients and visitors’ education model was applied for promoting hand hygiene and cough manners actions.

**Conclusions**

As the evaluation of education model, the percentage of observers allowed hand washing actions (86.09%) and cough manners (68.87%) after the education were increased. Therefore, the patients and visitors’ education model is efficient in promoting hand washing and respiratory hygiene compliance.

## Session: Hand hygiene

### H16 The effectiveness of alcohol-based hand rub bottle holder: an assessment with hand hygiene compliance rate and a satisfaction of emergency department personnel.

#### Sumawadee Skuntaniyom, Kumthorn Malathum, Pirawadee Tipluy

##### Ramathibodi Hospital, Bangkok, Thailand

###### **Correspondence:** Sumawadee Skuntaniyom (su_meaw@hotmail.com)

**Background**

Hand hygiene (HH) is a known effective measure for the prevention of healthcare-associated infections and spread of antimicrobial resistant organisms. Unfortunately a compliance rate with healthcare workers in emergency department is very low because of rushed working environment. The objective of this study focused on the interventions at the point of care (POC) to improve the compliance, or “system change” according to the WHO hand hygiene strategies, which key aspect was the provision of alcohol based hand rub on a new design bottle holder that optimized the acceptance and usage.

**Materials and methods**

The first phase was the baseline period set on 3 months baseline (January to March 2015) observation of HH compliance and continue the second phase using the bottle holder (April to June 2015). An anonymous, self-administered questionnaire, had distributed to 126 emergency healthcare workers (HCWs) to assess their behaviors and attitudes toward hand hygiene compliance and their satisfaction.

**Results**

Overall, 86.5% (109 of 126)of HCWs (36 nurses, 19 physicians) satisfied with the device and 87.3% believed they could improve HH compliance.Overall compliance significantly increased from 19.7% to 59% (P-valve = .045), 0 to 27.3% before patient contact, 11.5% to 57.7% after patient contact, 27.3% to 60.0% before clean/aseptic procedures, 48.1% to 64.0% after body fluid exposure/risk, and 17.1% to 61.9% after touching patient surroundings.

**Conclusions**

The device successfully provide easy access to the bottle holder, which is critical for its success in improving HH compliance in one of the busiest area in the hospital.

## Session: Hand hygiene

### H17 Electronic hand hygiene auditing tool in a Thai tertiary care hospital

#### Sangwan Paengta, Ratchanee wongsaen, Sutthiphun thanomphan, Samettanet Tariyo

##### Infection Control and Prevention Unit, Nakornping Hospital, ChaingMai, Thailand

###### **Correspondence:** Sangwan Paengta (kundaeng11@gmail.com)

**Background**

Hand Hygiene compliance among hospitalized personnel is a one of safety indicator. In the past, our hospital had used the paper-based system for auditing hand hygiene compliance. The majority of the data entry, collection and analysis were performed manually which was complicated and time-consuming.We aimed to create an electronic Hand Hygiene Auditing Tool to measure hand hygiene compliance.

**Materials and methods**

The study was conducted in a tertiary-care hospital in Thailand. A development process using five steps of the Program Development Life cycle(PDLC). 1) Requirement gathering and analysis 2.)Design on Google Docs access methods transition.3.)Program testing. 4.) Implementation on mobile phones. 5.) Maintain the program/system. All steps were conducted in a month (April 2016). The Electronic Hand Hygiene Auditing Tool is the result of this study.

**Results**

Both methods of data collection were compared. The results demonstrated that the duration of the processes decreased from 5 months to 1 month. Questionnaires return rate increased from 86% to 100%.The assessors’ satisfaction rate was 80%.

**Conclusions**

The Electronic Hand Hygiene Auditing Tool was qualified by reducing the number of hours. Rapid assessment and real time reporting provided valuable information to Infection Control and Prevention team.

## Session: Hand hygiene

### H18 Effects of using hand hygiene program to knowledge and infection prevention and control practices for multi-drug resistant organisms (MDROs) among nursing personnel and patients with care-givers in Surgical and Orthopedic Department in Nakornping hospital

#### Buachan Thongchuea, Pattama Khamfu, Sutthiphan Thanomphan

##### Nakornping Hospital, Chiang Mai, Thailand

###### **Correspondence:** Sutthiphan Thanomphan (kaynine@hotmail.co.th)

**Background**

Hand hygiene is the efficiency and low cost policy to prevent hospital-associated infections (HAIs) with multi-drug resistant organisms (MDROs). The quasi- experimental research aimed to evaluate the effect of the Hand Hygiene Program (HHP) on knowledge and practices in prevention of MDROs. Participants are 27 ICWNS, 140 RNs, 75 nurse aides, and 218 patients and their caregivers.

**Material and methods**

The study design was before-and after design, conducted during 1 February 2016 to 31 August 2016 in Surgical department and Orthopedics department. Participants were examined by questionnaire regarding knowledge about hand hygiene to assess their knowledge.A structured observation form was used to evaluate hand hygiene practice.The questionnaire in which the content validity was examined by 5 panel experts and the validity index was 0.8.The reliability observer was 1. Data were analyzed by Student’s t-test and descriptive statistics as appropriate.

**Results**

The study revealed that participants’ knowledge in hand hygiene increased from 75.7% to 89.0% in ICWNs, from 64.5% to 80.4% in nursing personnel, and from 62.3% to 79.4% in patients and care givers for significant ( p <0.001). Hand washing rates increased from 43.0% to 51.1% in moment 1, and from 66.5% to 92.7% in moment 5.

**Conclusions**

The collaboration of the ICWN network in Surgical and Orthopedics department contribute to the successful in increasing hand hygiene knowledge and behavior motivation. Nevertheless HAIs with MDROs was increased in this study. Other measures such as isolation precautions may be needed.

## Session: Hand hygiene

### H19 Impact of a multimodal intervention programme on hand hygiene compliance at the paediatric intensive care unit and immunocompromised ward of King Chulalongkorn Memorial hospital, Bangkok, Thailand

#### Wipaporn Natalie Songtaweesin^1^, Suvaporn Anugulruengkit^1,2^, Rujipat Samransamruajkit^3^, Darintr Sosothikul^4^, Ornanong Tansrijitdee^5^, Anry Nakphunsung^5^, Patchareeyawan Srimuan^1^, Jirachaya Sophonphan^2^, ThanyaweePuthanakit^1,2^

##### ^1^Department of Pediatrics, Faculty of Medicine, Chulalongkorn University, Bangkok, Thailand; ^2^Research Unit in Pediatric Infectious Diseases and Vaccines, Faculty of Medicine, Chulalongkorn University, Bangkok, Thailand; ^3^Division of Critical Critical Care, Faculty of Medicine, Chulalongkorn University, Bangkok, Thailand; ^4^Division of Hematology-Oncology, Faculty of Medicine, Chulalongkorn University, Bangkok, Thailand; ^5^Department of Pediatrics, King Chulalongkorn Memorial Hospital, Bangkok, Thailand

###### **Correspondence:** Wipaporn Natalie Songtaweesin (wipaporn@doctors.org.uk)

**Background**

Good hand hygiene (HH) practices are a simple and cost-effective strategy to limit pathogen transmission between patients. This study explores the effect of a multimodal hand hygiene promotion program on HH compliance amongst healthcare workers.

**Materials and methods**

A prospective study was conducted at the pediatric intensive care unit (PICU) and pediatric immunocompromised ward at King Chulalongkorn Memorial Hospital, Bangkok, Thailand. Interventions performed were: HH promotion videos sent to staff via mobile phone, hand hygiene signs at the bedside, distribution of portable alcohol gel bottles, and HH promotion culture led by senior staff members. All interventions were tailored according to pre-intervention opinion surveys with staff. HH compliance was assessed by direct observation using the WHO 5-moments for hand hygiene (WHO5HH) – before touching patients, before clean/aseptic procedures, after body fluid exposure risk, after touching patients, and after touching patient surroundings. 200 opportunities in total were observed monthly.

**Results**

In December 2015, pre-intervention, overall HH compliance rates were 50%. Between January and June 2016, post-intervention, overall HH compliance increased to 72%. When divided into the five moments for hand hygiene, hand washing prior to touching patients significantly improved following intervention from 43.8% to 85.1% (p < 0.001) on the immunocompromised ward and from 44.4% to 88.9% (p < 0.001) on the PICU. Hand hygiene after touching patient surroundings remained low. HH compliance was highest amongst nurses.

**Conclusions**

A multimodal HH promotion campaign tailored towards the local population was effective in increasing HH compliance overall. However, HH after touching patient surroundings remained low post campaign.

## Session: Hand hygiene

### H20 Implementation of multimodal hand hygiene improvement strategies in government hospitals

#### Kunyanut Payuk^1^, Wilawan Picheansathian^2^, Nongkran Viseskul^2^

##### ^1^Chao Phya Abhaibhubejhr Hospital, Prachinburi, Thailand; ^2^Faculty of Nursing, Chiang Mai University, Chiang Mai, Thailand

###### **Correspondence:** Kunyanut Payuk (kunyanut_nuch@hotmail.co.th)

**Background**

The World Health Organization (WHO) has developed multimodal hand hygiene improvement strategies to support hospitals to improve hand hygiene and thus reduce hospital-acquired infection. The aim of the study was to study the implementation of these multimodal strategies, obstacles, and supports to promote hand hygiene in government hospitals including university hospitals, regional hospitals, and general hospitals.

**Materials and methods**

The samples were 59 infection control nurses who implement multimodal hand hygiene improvement strategies. The data collection instrument was the Hand Hygiene Self-Assessment Framework questionnaire developed by the WHO. The questionnaire also asked about obstacles and support.

**Results**

Most of the samples (61.02%) implemented multimodal hand hygiene improvement strategies at an intermediate level, followed by basic and advanced levels (22.03% and 16.95%, respectively). Fifty percent of the university hospitals had an advanced level. Most of the regional hospitals and the general hospitals (57.14% and 67.57% respectively) had an intermediate level. The highest score for implementation was the system change for hand hygiene (median score, 90.00), followed by reminders in the workplace, training and education, evaluation and feedback, and institutional safety climate for hand hygiene (median score, 72.50, 60.00, 60.00 and 50.00, respectively). All samples encountered obstacles and needed support when implementing hand hygiene improvement programs. Personnel were the first obstacle, followed by management, facilitative equipment, and budget, respectively. The greatest need was for environmental and equipment support, followed by support from staff and management support.

**Conclusions**

The government hospitals should improve implementation of multimodal hand hygiene strategies by addressing obstacles and providing support in order to promote hand hygiene and reduce hospital-acquired infection.

## Session: Hand hygiene

### H21 Can alcohol based hand formulations be used in the entire hospital as an antiseptic and preoperative hand disinfection?

#### Elizabeth DeNardo^1^, Rachel Leslie^1^, Todd Cartner^1^, Luciana Barbosa^1^, Heinz-Peter Werner^2^, Florian H.H Brill^3^, Julia Yaeko Kawagoe^4^

##### ^1^GOJO Industries, Inc., Akron, OH, USA; ^2^HygCen International GmbH, Schwerin, Germany; ^3^Dr. Brill+Partner GmbH, Hamburg, Germany, Hospital Albert Einstein, Sao Paulo, Brazil; ^4^Hospital Albert Einstein, Sao Paulo, Brazil

###### **Correspondence:** Elizabeth DeNardo (denardoe@gojo.com)

**Background**

In health care settings alcohol based hand antiseptics can be used with two different purposes: 1- as a hand rub (ABHR) used by health care workers to reduce the load of transient microorganism from their hands during patient care; 2-as a preoperative hand disinfection (ABSS) used by the surgical staff to eliminate transient microorganisms, reduce resident microorganisms from the hands, and maintain microorganism levels below baseline for the duration of surgery. A product must meet established regulatory standard criteria to be used in the entire hospital. This study aimed to evaluate the antimicrobial efficacy, skin compatibility, and end user acceptance of two new alcohol based formulations.

**Material and methods**

Two novel ABHR gel and foam 70% ethanol were tested by ASTM E1174 and European Norm (EN) 1500 for the in vivo hygienic hand rub, and by EN12791 for surgical hand disinfection. Skin irritation was evaluated according to the 21-Day Cumulative Irritation procedure and skin hydration measured by corneometer device. End user acceptance was conducted during trial periods through surveys.

**Results**

Both formulations met all the in vivo efficacy tests to be considered as ABHR and ABSS with sustained effect of 3 hours. No potential skin irritation was demonstrated and end user showed preference over other products.

**Conclusions**

Well-formulated ABHR gel and foam containing just 70% ethanol meets global efficacy norms for ABHR and ABSS, are mild to the skin, and accepted by end users. Both formulations are recommended to the entire hospital following specific protocols of usage for ABHR and ABSS as described by the World Health Organization.

## Session: Hand hygiene

### H22 Not all alcohol-based hand rubs are equal: formulation is a critical component for the antimicrobial efficacy and is more important than alcohol concentration alone

#### Elizabeth De Nardo^1^, Sarah Edmonds- Wilson^1^, David Macinga^1^, Patricia Mays-Suko^2^, Collette Duley^2^

##### ^1^GOJO Industries, Inc, Akron, Ohio, USA; ^2^Bioscience laboratories, Inc, Bozeman, Montana, USA

###### **Correspondence:** Elizabeth De Nardo (denardoe@gojo.com)

**Background**

Many alcohol based hand rub (ABHR) formulations with different alcohol concentrations and delivery formats are available in the market, making difficult the selection of those products.In this study we compared the in vivo antimicrobial efficacy of commercial available ABHR and the World Health Organization (WHO) recommended formulation used as benchmarks and determine the key factors that influence the antimicrobial efficacy.

**Materials and methods**

Two novel ABHR formulations (70% ethanol gel and foam) were evaluated according to global standards protocols: American Society for Testing and Materials ASTM E1174 (Health Care Personnel Hand wash [(HCPHW)] and European Norm (EN) 1500. Additionally, using E1174, the efficacy of these formulations was compared against seven commercially available ABHRs and WHO recommended formulations containing alcohol from 60% to 90% at a more realistic 2-mL volume application.

**Results**

The novel ABHR formulations met efficacy requirements for both HCPHW and EN 1500 when tested at volumes typically used in these methods. Moreover, these formulations met HCPHW requirements when tested at 2-mL. In contrast, the commercial ABHRs and World Health Organization formulations failed to meet HCPHW using a 2-mL application. Importantly, product efficacy did not correlate with alcohol concentration and format.

**Conclusions**

ABHR are complex formulations, combining alcohol with various ingredients that can influence the overall antimicrobial efficacy. Formulation as a whole and is more important than alcohol concentration alone for efficacy. Product format has the same efficacy. When selecting ABHR, ask for the in vivo efficacy results based on standard protocols and the volume of the product used to meet the criteria for HCPHW test.

## Session: Hand hygiene

### H23 Effectiveness of education, observation and feedback to hand hygiene compliance in healthcare workers

#### Phan Thi Hang^1^, Tran Thi Thuy Hang^2^, Tran Thi My Hanh^2^, Christopher Gordon^3^

##### ^1^Quality Management Division, Hung Vuong Hospital, Ho Chi Minh City, Vietnam; ^2^Infection Control Department, Hung Vuong Hospital, Ho Chi Minh City, Vietnam; ^3^University of Sydney, New South Wales, Sydney, Australia

###### **Correspondence:** Tran Thi Thuy Hang (thuyhangytcc@gmail.com)

**Background**

Hand hygiene compliance of healthcare workers (HCWs) is very important. This research aimed to determine the improvement of knowledge and hand hygiene compliance following an educational program and observation, feedback strategy at some important departments of Hung Vuong hospital, a tertiary obstetric and gynecological hospital in Vietnam. We aimed to determine the hand hygiene compliance rate and the mean knowledge score before –after the interventions.

**Materials and methods**

Intervention started from September 2014 to May 2015, included three periods: (1) pre-intervention to determine hand hygiene compliance rate and knowledge score, (2) holding training classes, auditing and feed backing monthly, (3) post-intervention to re-determine compliance rate and knowledge score.To assess HCWs knowledge and compliance, we used WHO Hand Hygiene Knowledge Questionnaire for Health-Care Workers and Observation Form.

**Results**

Two hundred and six participants were included. The total of hand hygiene opportunities is 3151. All participants significantly improved knowledge scores from baseline to 2 months post educational intervention with mean difference (SD): 1.5 (2.5); p < 0.001).Hand hygiene compliance increased significantly from baseline across all sites (43.6% [95%CI: 41.1-46.1] to 63% [95%CI: 60.6-65.3]; p < 0.0001). The compliance rate of categories and “Five hand hygiene moments” compliance rate also had improvement.

**Conclusions**

Training and observation with feedback on hand hygiene has improved hand hygiene knowledge and compliance rate of HCWs.

## Session: Hospital Epidemiology& Surveillance

### HE1 Cardiac surgery infection prevention monitoring (CSIPM) tool-results of pilot study

#### Dorairajan Sureshkumar^1^, Roopa Durairaj^2^, Anusha Rohit^1^, Saujanya Saravanakumar^3^, Jothymani Hemalatha^4^

##### ^1^Madras Medical Mission, Chennai, Tamil Nadu, India; ^2^SRM Institutes for Medical Sciende (SIMS) Hospital,Chennai, India; ^3^Kasturba Medical College Manipal, Karnataka, India; ^4^100 Degree Fahrenheit (F), Chennai, India

###### **Correspondence:** Dorairajan Sureshkumar (dskinfdis@gmail.com)

**Background**

Post -operative infections are the leading cause of non-cardiac complications after cardiac surgeries in the developing world. Most of these infections are preventable, if the prevention measures were strictly followed. However, monitoring of these parameters is difficult in resource-limited settings. Here we reported initial results of infection prevention monitoring using CSIPM tool.

**Materials and methods**

This cross sectional study was carried out to pilot test the usage of CSIPM tool by infection prevention professionals in five cardiac centers in Chennai among patients undergoing cardiac surgeries. This CSIPM tool was created based on the Association for Professionals in Infection Control and Epidemiology (APIC) guidelines that included parameters such as antibiotic timing, antibiotic choice, hair removal technique, skin preparation, and pre-operative MRSA screening and intranasal mupirocin application.

**Results**

51 patients who underwent cardiac surgeries in 5 centers were assessed using CCSIPM tool. The results are as follows. Infection Prevention parameter Centre 1 (13 patients) Centre 2 (9 patients) Centre 3 (10 patients) Centre 4 (7 patients) Centre 5 (12 patients) were participated. First dose of antibiotic prophylaxis (within 1 hour of incision) was 40/51 (78.43%). Choice of prophylactic antibiotics (as per guidelines) was 45/51 (88.23%). Hair removal methods (clippers use) was 30/51 (58.82%) Surgical site skin preparation (cholrhexidine based methods) was 23/51 (45.09%). Pre-op MRSA screening was 0/51 (0%) Universal mupriocin administration was 21/51 (41.17%).

**Conclusions**

The CSIPM tool was easy to monitor infection prevention measured in cardiac surgery patients in Chennai. There was wide variability in implementing infection prevention measured among hospitals. There was an urgent need to test CSIPM tool in large multicenter studies before universal recommendation.

## Session: Hospital Epidemiology& Surveillance

### HE2 Retrospective study of mortality and species distribution among patients with *Candida* bloodstream infection: a single hospital experience in japan

#### Ryuichi Hirano^1,5^, Yuichi Sakamoto^2^, Shoji Yamamoto^3^, Naoki Tachibana^4^

##### ^1^Department of Pharmacy, Aomori Prefectural Central Hospital, Aomori, Japan; ^2^Department of Laboratory Medicine, Aomori Prefectural Central Hospital, Aomori, Japan; ^3^Department of Pharmacy, Aomori Prefectural Central Hospital, Aomori, Japan; ^4^Department of Laboratory Medicine, Aomori Prefectural Central Hospital, Aomori, Japan; ^5^Department of Clinical Laboratory Medicine, Hirosaki University Graduate School of Medicine, Aomori, Japan

###### **Correspondence:** Ryuichi Hirano (r.hirano1115@gmail.com)

**Background**

The mortality rate among patients with bloodstream infection caused by *Candida* species is relatively high. However, prognostic factors and species distribution of this disease have not yet been elucidated in detail. The aim of this study was to examine prognostic factors for candidemia using the mortality rate and *Candida* distribution among patients with candidemia.

**Materials and methods**

Seventy-five patients diagnosed with candidemia in Aomori Prefectural Central Hospital between January 2007 and December 2013 were enrolled in this study. Mycological data were collected from the records of each patient’s blood culture tests. Odds ratios (ORs) for death were analyzed using a multivariate stepwise logistic regression analysis.

**Results**

Twenty (26.6%) patients died within 30 days of being diagnosed with candidemia. Advanced age (OR = 1.1, 95% confidence interval = 1.01-1.23, *P* = 0.04) was a significant risk factor for a high mortality, whereas removal of a central venous catheter (OR = 0.03, 95% confidence interval = 0.002-0.3, *P* = 0.01) was associated with a lower mortality rate. Seventy-six *Candida* species were isolated from blood cultures; *Candida albicans* 28 (36.8%), *Candida parapsilosis* 23 (30.2%), *Candida guilliermondii* 16 (21.0%), *Candida glabrata* four (5.2%), *Candida tropicalis* two (2.6%), and *Candida* species three (3.9%) that could not be identified. The 30-day mortality rates for *Candida albicans* was 42% (12/28), which was significantly higher than that of *Candida parapsilosis* (4.3%, 1/23).

**Conclusions**

This study indicated that persistent of central venous catheter was detrimental to patients with candidemia. Close attention may be required in cases of elderly patients.

## Session: Hospital Epidemiology& Surveillance

### HE3 Post-quake infection control intervention on nursing homes for seniors

#### Miho Miura, Fumiyo Hieda, Yoshiro Sakai, Hiroshi Watanabe

##### Division of Infection Control and Prevention, Kurume University Hospital, Kurume, Japan

###### **Correspondence:** Miho Miura (miura_miho@kurume-u.ac.jp)

**Background**

After the earthquake in Kumamoto on April 14th, 2016, Disaster Medical Assistance Team (DMAT) and Japan Medical Association Team (JMAT) immediately supported the shelters and medical facilities in the disaster area. However, the arrivals of DMAT and JMAT to nursing homes were delayed due to lack of information and no transportation by landslides. We started supporting nursing homes as JMAT members 16 days after the quake. This study aimed to report about our infection control intervention in the disaster area.

**Materials and methods**

We conducted a survey on the occurrences of infectious gastroenteritis and influenza at nursing homes, and interviewed the healthcare workers about problems on infection control. Then, we offered guidance on infection control necessary to each facility.

**Results**

We visited eleven nursing homes between April 30th and May 2th, 2016 to find two residents with symptoms of influenza and one with symptoms of infectious gastroenteritis. The most frequent questions from the healthcare workers at nursing homes were how to clean and sterilize medical devices, and how to clean their hands appropriately.

**Conclusions**

It is important to educate health care workers at nursing homes on a regular basis regarding infection control, while the occurrence time of infection varies depending on the disaster’s type and scale.

## Session: Hospital Epidemiology& Surveillance

### HE4 Microbiological profile of device-related healthcare infections

#### Melecia Velmonte, Silverose Ann Bacolcol, Allan Alde, Keitleen Chavez, Arlene Joy Esteban, Aisa Jensen Lee

##### Infection Prevention and Control Office, Manila Doctors Hospital, Manila, Philippines

###### **Correspondence:** Melecia Velmonte (silveroseann@yahoo.com)

**Background**

The Manila Doctors Hospital – Infection Prevention and Control Office (IPCO) reviewed the surveillance data of device-related healthcare infections in Intensive Care Unit (ICU). This study aimed to determine the prevalence of device-related healthcare infections in ICU, identify most frequently isolated organisms and its antibiotic susceptibility.

**Materials and methods**

Profile of pathogens isolated from patients with device-related infections (DRI) was analyzed from surveillance data collected by Infection Prevention and Control nurse from 2014 to 2016.

**Results**

Three years surveillance data of DRI revealed 32 DRI developed among patients, nineteen (19) ventilator-associated pneumonia (VAP), seven (7) central line-associated blood stream infection (CLABSI), and six (6) catheter-associated urinary tract infection (CAUTI). *Klebsiella pneumoniae* Carbapenemase (+) KPC (8), *Serratia marcescens* (3), and *Stenotrophomonas maltophilia* (3), were the pathogens isolated from VAP, MRSA (4) and *Acinetobacter baumannii* (2) from CLABSI. *E. coli* and *Klebsiella pneumoniae* both ESBL (+) and *Enterococcus faecalis* were isolated from patients with CAUTI. *Klebsiella pneumoniae* carbapenemase positive was sensitive only to three antibiotics: amikacin, gentamicin and colistin. The carbapenems, aminoglycocides, extended spectrum penicillin, ciprofloxacin, levofloxacin were still effective against most of the gram negative bacteria isolated. MRSA was sensitive only to linezolid, daptomycin and vancomycin.

**Conclusions**

The most antibiotic-resistant pathogen KPC was isolated in patients with VAP on prolonged mechanical ventilation and MRSA in patients with central line of more than two weeks. It is recommended that devices be removed when no longer required to reduce risk of DRI, overuse of antibiotics, and emergence of antibiotic resistant strains. Strict implementation of infection prevention and control practices should be enforced.

## Session: Hospital Epidemiology& Surveillance

### HE5 A 3-year comparison of methicillin-resistant *Staphylococcus aureus* (MRSA) epidemiology in an acute-care hospital and affiliated intermediate-and long-term care facilities

#### Angela Chow, Jia-Wei Lim, Pei-Yun Hon, Aung-Aung Hein, Grace Tin, Vanessa Lim, Brenda Ang

##### Tan Tock Seng Hospital, Singapore, Singapore

###### **Correspondence:** Angela Chow (Angela_Chow@ttsh.com.sg)

**Background**

Methicillin-resistant *Staphylococcus aureus* (MRSA) is the most common healthcare-associated multidrug resistant organism. Despite the interconnectedness between acute hospitals and intermediate- and long-term care facilities (ILTCs), the comparative epidemiology of MRSA between the institutions is not well understood. This study aimed to compare the epidemiology of MRSA in an acute hospital with its affiliated ILTCs longitudinally.

**Materials and methods**

We conducted period prevalence surveys screening for MRSA in June-July 2014-2016, in an acute hospital (AH) and affiliated intermediate-care (ITC) and long-term care (LTC) facilities in Singapore. Nasal, axilla, and groin swabs were cultured on selective chromogenic agar. Multivariable logistic regression models were constructed for comparison of MRSA prevalence.

**Results**

A total of 5458 patients were screened. MRSA prevalence was higher in ITCs (36.6%) than LTCs (22.2%) and AH (12.4%) (P < 0.001). In ITCs, MRSA prevalence increased significantly from 29.9% (2014) to 45.7% (2015) (P < 0.001) and decreased to 32.4% (2016) (P < 0.001). In LTCs, MRSA prevalence was similar in 2014 (20.4%) and 2015 (19.8%), but increased to 25.6% in 2016 (P = 0.049). MRSA prevalence in AH remained stable (2014: 11.8%, 2015: 13.5%, 2016: 11.9%, P = 0.44). Median length of stay (LOS) in LTCs (439 days) was significantly higher than in ITCs (22 days, P < 0.001) and AH (9 days, P < 0.001). After adjusting for age, gender, LOS, and year of screening, MRSA colonization was significantly higher in ITCs (OR 4.28, 95%CI 3.65-5.03) and LTCs (OR 2.21, 95%CI 1.80-2.71) than the AH.

**Conclusions**

MRSA prevalence is higher in ILTCs than the AH. Active surveillance and infection control strategies at acute hospitals should also be instituted at ILTCs.

## Session: Hospital Epidemiology& Surveillance

### HE6 Comparative epidemiology of vancomycin-resistant *enterococcus* (VRE) in an acute-care hospital and its affiliated intermediate- and long-term care facilities: a 3-year longitudinal study

#### Angela Chow, Aung-Aung Hein, Jia-Wei Lim, Pei-Yun Hon, Vanessa Lim, Grace Tin, Brenda Ang

##### Tan Tock Seng Hospital, Singapore, Singapore

###### **Correspondence:** Angela Chow (Angela_Chow@ttsh.com.sg)

**Background**

Vancomycin-resistant *Enterococcus* (VRE) infections in acute hospitals are increasing, but little is known about VRE colonization in intermediate- and long-term care facilities (ILTC). This study aimed to make longitudinal comparisons on the epidemiology of VRE in an acute hospital and its closely-affiliated ILTCs.

**Materials and methods**

We conducted period prevalence surveys screening for VRE in June-July 2014-2016, in an adult acute tertiary-care hospital (AH) and 6 of its affiliated ILTCs, in Singapore. Stool or rectal swabs were obtained and cultured using selective chromogenic agar. Epidemiologic data was obtained from electronic medical records. To compare differences in VRE prevalence, multivariable logistic regression models were constructed.

**Results**

Of 5359 patients screened, 9.8% were VRE colonized. VRE prevalence was significantly higher in the AH (14.2%) than in intermediate-care (ITC) (7.6%) and long-term care (LTC) (0.8%) facilities (P < 0.001). In the AH, VRE prevalence decreased from 19.8% in 2014 to 14.0% in 2015 to 8.9% in 2016 (P < 0.001). In ITCs, VRE prevalence increased from 5.1% in 2014 to 9.9% in 2015 (P = 0.012) and was 7.4% in 2016 (P = 0.19). VRE prevalence remained low in LTCs (2014: 0.3%, 2015: 0.8%, 2016: 1.1%, P = 0.45). After adjusting for age, gender, length of stay, and year of screening, VRE colonization was significantly higher in the AH than ITCs (OR 1.96, 95%CI 1.55-2.48, P < 0.001) and LTCs (OR 22.52, 95%CI 9.95-50.97, P < 0.001).

**Conclusions**

VRE prevalence was highest in the AH but has declined over the years. Prevention and control efforts in acute hospitals should be enhanced to further reduce VRE colonization and prevent transmission to ILTCS.

## Session: Hospital Epidemiology& Surveillance

### HE7 How different is the epidemiology of carbapenem-resistant *enterobacteriaceae* (CRE) in an acute-care hospital from its affiliated intermediate- and long-term care facilities?

#### Angela Chow, Grace Tin, Aung-Aung Hein, Vanessa Lim, Jia-Wei Lim, Pei-Yun Hon, Brenda Ang

##### Tan Tock Seng Hospital, Singapore, Singapore

###### **Correspondence:** Angela Chow (Angela_Chow@ttsh.com.sg)

**Background**

Carbapenem-resistant *Enterobacteriaceae* (CRE) colonization in acute hospitals is increasing, but little is known about CRE colonization in intermediate- and long-term care facilities (ILTC). This study aimed to compare the epidemiology of CRE longitudinally in an acute hospital and affiliated ILTCs.

**Materials and methods**

Cross-sectional studies were conducted with CRE screening in June-July 2014-2016, in an adult acute tertiary-care hospital (AH) and its 6 closely-affiliated ILTCs in Singapore. Stool or rectal swabs were cultured using selective chromogenic agar. Epidemiologic data were obtained from electronic medical records. Multivariable logistic regression models were then constructed for independent comparison of CRE prevalence between healthcare facilities.

**Results**

A total of 5359 patients were screened (2014: 1675, 2015: 1794, 2016: 1890). CRE prevalence was significantly higher in the AH (2.13%) than in intermediate-care (ITC) (1.29%) and long-term care (LTC) (0.35%) facilities (P < 0.001). CRE prevalence remained low In the AH (2014: 2.25%, 2015: 2.06%, 2016: 2.08%, P = 0.96) and ITCs (2014: 1.13%, 2015: 0.44%, 2016: 2.30% P = 0.045). Only 4 patients (all in 2015, 1.1%) were colonized with CRE in LTCs. Median length of stay (LOS) in LTCs (439 days) was higher than in ITCs (22 days, P < 0.001) and AH (9 days, P < 0.001). After adjusting for age, gender, LOS, and year of screening, CRE colonization was higher in the AH (OR 4.11, 95%CI 1.16-14.53) and ITCs (OR 2.49, 95%CI 0.66-9.32) than LTCs.

**Conclusions**

CRE colonization has remained low in the AH and ITCs, and negligible in LTCs. However, further studies are required to identify high-risk patients for targeted surveillance, to prevent CRE from becoming endemic.

## Session: Hospital Epidemiology& Surveillance

### HE8 Risk factors and causes of death in dengue fever– a retrospective study at one medical center in south Taiwan

#### Huwi-chun Chao, Chiu-Yin Yeh

##### Chi Mei Medical Center, Intensive Care Medicine, Yongkang, Taiwan

###### **Correspondence:** Huwi-chun Chao (lgi0324@yahoo.com.tw)

**Background**

As Taiwan is situated in the high risk subtropical region, dengue fever has virtually become a seasonal infectious disease. This study aimed to determine risk factors and causes of death in dengue fever.

**Materials and methods**

Analysis was conducted on the 93 confirmed severe cases of dengue fever or dengue hemorrhagic fever reported to this hospital over the period between July 20 and September 30, 2015 in terms of gender, age, history of chronic diseases, warning signs and diagnostic criteria for severe conditions. Retrospective case study was also conducted to identify risk factors in dengue fever and dengue hemorrhagic fever as well as predictors of death among dengue fever cases for statistical analysis.

**Results**

Those susceptible to infection concentrated on older people aged over 65 (with an average age of 68); in total 73 cases had chronic diseases (with an average rate of 78.5%), among which hypertension and diabetes constituted the majorities; and based on symptoms, fever accounted for 83.51% while gastrointestinal bleeding was the most common at 38.7%. Of the 93 cases, there were 18 deaths.

**Conclusions**

It’s suggested that older people aged over 65 and with chronic diseases who are infected with dengue hemorrhagic fever must be closely monitored. To sum up, effective use of knowledge about risk factors and prognostic factors in dengue hemorrhagic fever can help epidemic prevention organizations to focus their limited resources on high risk groups and increase the effectiveness of prevention.

## Session: Hospital Epidemiology& Surveillance

### HE9 The exploration of risk factors for patients with severe influenza infection

#### Mei-feng Lo, Huwi-chun Chao

##### Intensive care unit, Chimei medical center, Tainan, Taiwan

###### **Correspondence:** Mei-feng Lo (t1901141@yahoo.com.tw)

**Background**

The importance of influenza is its rapid spreading of the epidemic in a wide range and serious complications, especially bacterial and viral pneumonia. Taiwan medical and public health system experienced huge impacts of such epidemics. Our study integrated epidemiological results to improve the effectiveness of influenza surveillance and assess the effectiveness of health policies for influenza prevention.

**Materials and methods**

We retrospectively reviewed recorded data of patients admitted in the intensive care unit who were diagnosed as severe influenza with respiratory failure during January 1, 2015 to December 31, 2015. We also recorded their clinical outcomes and history of influenza vaccine administration. We analyzed their prognosis, predictors of death and their possible correlations.

**Results**

Sensitivity analysis was performed to assess the severity of patients admitted in the ICU. Mechanical ventilation was estimated for epidemiological week of hospitalization. These patients had low proportion of treatment with antiviral medication in the first period of the study.

**Conclusions**

Influenza deaths were increased. It may contributed from aging of the population, underestimated the needs for better prevention modalities, including more effective vaccines and vaccination programs for elderly persons.

## Session: Hospital Epidemiology& Surveillance

### HE10 Incidence of carbapenem–resistant Enterobacteriaceae in Suratthani Hospital

#### Chonlada Piwpong (chonladapiwpong@gmail.com)

##### Infection Control Nurse, Suratthani Hospital, Suratthani, Thailand

**Background**

Carbapenem–resistant Enterobacteriaceae (CRE) infection in the hospital is a major problem that has a direct impact to patients, health personnel and hospital. This study aimed to study the incidence rate of CRE and to investigate factors associated with CRE in the hospital.

**Materials and methods**

Data were collected by CRE from October, 1 2013 to September, 30 2015, analyzed by descriptive statistics, Chi-square test, Fisher’s exact test and Mann-Whitney U test.

**Results**

The results revealed that in 2015 the total of patient days was 281,622 days or 60,961 discharged patients. CRE infection was diagnosed 35 times. The nosocomial infection rate was 0.12 times/1000 patient days and 0.57 times/1000 discharged patient. The majority of infections were ventilator-associated pneumonia (34.80%); catheter-related urinary tract infection (30.40%). Pathogens were *Klebsiella pneumoniae* (60.70%), *Escherichia coli* (26.80%) and *Enterobacter cloacae* (8.93%). Most of the patients were immunocompromised host (92.70%), received catheter insertion (80.00%). Admitted in medical ward (46.30%), followed by surgical ward (42.00%). Patients with CRE infection had a higher cost of antibiotic and a longer length of stay than those who were not infected (111,864.00 and 115,23.25 baht; 67.00 and 26.70 days, respectively). Moreover, the length of stay in the hospital of the study group was significantly higher than those in the control group (p < .05).

**Conclusions**

Controlling and preventing CRE infection in medical ward and surgical ward were important and necessary. Especially among patients who were admitted in the hospital for longer than 4 weeks.

## Session: Hospital Epidemiology& Surveillance

### HE11 Changing antibiotic prophylaxis from fosfomycin to cefazolin did not change the trend of postcraniotomy surgical site infections

#### Songyos Rajborirug, Ploypailin Preechawetchakul, Yada Pruekrattananapa, Tharntip Sangsuwan, Silom Jamulitrat

##### Prince of Songkla University, Hat Yai, Songkla, Thailand

###### **Correspondence:** Songyos Rajborirug (ic_conference@yahoo.com)

**Background**

Routine use antimicrobial prophylaxis is an essential part of brain surgery due to the potential devastating consequences of postcraniotomy surgical site infection (PC-SSI). In the late of year 2014, after the decreasing rates of PC-SSI in Songklanagarind Hospital from vigorous control measures, we decided to change the antibiotic prophylaxis from fosfomycin (FOS) to cefazolin (CZO). This study aimed to evaluate the trend of PC-SSIs after changing antibiotic prophylaxis.

**Materials and methods**

The surveillance data were reviewed together with patient medical records to access the trend of PC-SSIs and identify the factors needed for risk adjustment.

**Results**

Information of 3250 craniotomies or craniectomies were reviewed and 157 PC-SSIs were documented in the patients underwent the operations during January 2005 through June 2016. Since the late of year 2007, FOS prophylaxis were used more frequently due to the recognition of methicillin-resistant *Staphylococcus aureus* outbreak. After the downward trend of PC-SSIs began in year 2012, prophylaxis was changed from FOS to CZO and the trend of PC-SSIs did not change after that.

**Conclusions**

Changing surgical prophylaxis from FOS to CZO did not increase the risk of PC-SSI.

## Session: Hospital Epidemiology& Surveillance

### HE12 Analysis of vancomycin-resistant enterococci (VRE) at a tertiary care hospital in northern Thailand: assessing epidemiology, cost and length of stay

#### Ratchanee Wongsaen^1^, Sungwan Paengta^1^, Napatnun Nilchon^2^, Sutthipun Thanompan^1^, Samattanet Tariyo^1^

##### ^1^Infection Control and Prevention Unit, Nakornping Hospital, ChaingMai, Thailand; ^2^Department of Laboratory , Nakornping Hospital, ChaingMai, Thailand

###### **Correspondence:** Ratchanee Wongsaen (wong_saen@hotmail.com)

**Background**

After the first identified at a tertiary care hospital in northern Thailand since 2014, VRE have spread rapidly and become a problem implicated in health care associated infections (HAIs). Our study aimed to analyze epidemiology, cost and length of stay among patients having VRE at a tertiary care hospital in northern part of Thailand.

**Materials and methods**

We conducted a one-year prospective study in 82 patients admitted in Inpatient Department from October 2015 to September 2016 who were colonized or infected with VRE. The data source for surveillance was laboratory-based system. Data were analyzed by using descriptive statistics.

**Results**

From October 2015 to September 2016, we found VRE in 82 cases (59.7% in female and 40.3% in male). The incidence rate of VRE was 0.03:1000 patient-days. Most cases were found in medical department (87.8%). Incidence of VRE-infected patients was 59.7% (49/82 cases). Majority of these cases (43/82 cases, 52.4%) were HAIs. Incidence of patients colonized with VRE was 40.3% (33/82 cases). The most common sites of the infections were catheter-associated urinary tract infections. The average of hospitalization cost and the cost of antibiotic therapy for each patient were 96,844 Baht and 30,721 Baht respectively. The average length of stay in hospital for each patient was 26.2 days.

**Conclusions**

As a result of this study, most of VRE-infected patients were HAIs. The impacts of the infection increased both in cost and length of hospital stay.

## Session: Hospital Epidemiology& Surveillance

### HE13 Incidence and risk factors of ventilator associated pneumonia in newborn at the national children’s hospital, Vietnam

#### Ngai Le, Dung Khu

##### National Children's Hospital, Dong Da, Hanoi, Vietnam

###### **Correspondence:** Ngai Le (ngai.lekien@nhp.org.vn)

**Background**

Newborn could be seen as patients who are potentially exposed to ventilator associated pneumonia (VAP) at high risk. Identify the incidence as well as risk factors for VAP in newborn would be much necessary for prevention application. This study aimed to identify incidence and risk factors of VAP in NICU at the National Children’s Hospital (NCH) in Vietnam.

**Materials and methods**

This cohort prospective study was conducted between January and December in 2012. After univariate analysis, multivariate regression analysis was used to handle all statistically significant risk factors**.**

**Results**

602 neonates with mechanical ventilation (MV) were enrolled in our study. Among them, 151 neonates with VAP were determined with 159 episodes. Incidence rate (IR) was 25.1% (151/602); incidence density rate (IDR) was 31.7/1000 ventilator days (159/5018 ventilator days). With univariate analysis, these risk factors of VAP in newborn were: very low birth weight (<1500 gr) (OR: 1.9;95%CI: 1.2-2.8), transfusion during MV (OR:4.51; 95%CI:1.35-3.10), Use of corticosteroids during MV (OR:2.23; 95%CI:1.2-4.13), use of vasopressor during MV (OR: 2.05;95%CI:1.35 -3.10), reintubation before occurrence of VAP (OR: 1.6; 95%CI:1.04 – 2.46), duration of MV > 10 days (OR: 2.3; 95%CI:1.4 – 3.6). With multivariate regression analysis, “blood transfusion during MV” and “duration of MV for > 10 days” were the risk factors of VAP in newborn (OR: 2.91 & 3.29; 95%CI: 1.06-8.02 & 1.004-10.8).

**Conclusions**

IR & IDR of VAP in newborn at the NCH in Vietnam still was high (25.1% & 31.7/1000 ventilator days); main risk factors for VAP in newborn were “blood transfusion during MV” and “duration of MV for > 10 days”

## Session: Microbiology

### M1 Antibiotic sensitivity dynamics of *Staphylococcus aureus*, taken from patients with soft tissue infections (SSTI)

#### Svetlana Kolesnichenko^1^, IlyaAzizov^2^, Alyona Lavrinenko^1^, Yerbol Tishkambayev^1^

##### ^1^Karaganda State Medical University, Karaganda, Kazakhstan; ^2^Institute of Antimicrobial Chemotherapy, Smolensk, Russia

###### **Correspondence:** Svetlana Kolesnichenko (svetofor08@gmail.com)

**Background**

It does not cease to be an actual problem of the skin and soft tissue infections. For several years *S. aureus* occupies the leading position among the agents of these diseases.This study aimed to reflect the dynamics of sensitivity to antibiotics of *Staphylococcus aureus* (S. aureus), taken from patients with STI.

**Materials and methods**

Evaluation of the sensitivity to antibiotics *S. aureus* was conducted by retrospective methods. Material - wound discharge of patients with STI. Isolation of the pathogen was carried out by classical bacteriological methods. Species belonging was determined by the time-of-flight methods of mass spectrometry. Sensitivity to antibiotics was determined by disk diffusion methods according to CLSI standards.

**Results**

From 2013 to 2015 years, 179 samples were studied. During the study period, the percentage of detection of *S. aureus* ranged between 55% and 75% (P > 0.05). There is 100% sensitivity of the isolated strains in 2013 and 2016 to oxacillin, cefoxitin and vancomycin; in year 2014 2.3% of strains were resistant to oxacillin and cefoxitin. Sensitivity to the aminoglycosides group also retained for three years, but in 2014 4% of strains were resistant to gentamicin. Sensitivity to rifampicin and linezolid remains at 100%. 2.2% of strains were isolated resistant to fluoroquinolones group in 2014. MLS-resistant strains in the amount of 10% were isolated in 2013. And in year 2014 17.6% strains were resistant to lincomycin and 13.7% to azithromycin.

**Conclusions**

*S. aureus* keeps leading position among pathogens of skin and soft tissue infections, without any increase of level of antibiotic-resistance (p > 0.05).

## Session: Microbiology

### M2 Urinary tract infections in pregnancy women

#### Alyona Lavrinenko^1^, Ilya Azizov^2^, Yerbol Tishkambayev^1^, Asylkhan Alibecov^1^,Svetlana Kolesnichenko^1^, Yekaterina Serbo^1^

##### ^1^Karaganda State Medical University, Karaganda, Kazakhstan; ^2^Institute of Antimicrobial Chemotherapy, Smolensk, Russia

###### **Correspondence:** Alyona Lavrinenko (lavrinenko.alena@gmai.com)

**Background**

Urinary tract infections are common and can progress to serious infectious complication in pregnant women. Epidemiology and antibiotic susceptibility are important to guide the most appropriate and safe treatment during pregnancy.

**Materials and methods**

A quantitative urine culture was done. Microorganisms was identified by MALDI-TOF (mass-spectrometer Microflex, Bruker). Determination of the antimicrobial susceptibility of the isolated strains was performed by disk diffusion methods, using MIC breakpoints from CLSI 2012 guidelines.

**Results**

The prevalence of urinary tract infections in pregnant women was 22.5%. There were 87 pregnant women with positive urine samples of 10^6-10^8 CFU/ml. *E. coli* was the most common isolated pathogen (55.2%). The most active antibacterial agents against *E.coli* were fluoroquinolones. All *E. coli* strains were susceptible to fosfomycin, trimethoprim, amikacin, isepamicin, netilmicin. The susceptibility of nitrofurantoin was 93.8%, ampicillin-sulbactam was 97.3%, gentamicin and sisomicin were 95.2%, and ampicillin was 53.3% among *E. coli* strains

**Conclusions**

*E. coli* was the most common causative agent of urinary tract infections in pregnant women in this study. Antibiotics with highest susceptibility are not considered safe according to their pregnancy category. Nitrofurantoin was likely to be the appropriate treatment in terms of safety and high susceptibility.

## Session: Microbiology

### M3 Evaluation of phenotypic screening tests for carbapenemase-producing Enterobacteriaceae

#### Youngwon Nam^1^, Jae Hyeon Park^1^, Yun Ji Hong^2^, Taek Soo Kim^3^, Jeong Su Park^2^, Kyoung Un Park^2^, Eui-Chong Kim^3^

##### ^1^Armed Forces Daejeon Hospital, Daejeon, Korea; ^2^Seoul National University Bundang Hospital, Seoul, Korea; ^3^Seoul National University Hospital, Seoul, Korea

###### **Correspondence:** Taek Soo Kim (fish9422@me.com)

**Background**

Rapid screening and identification of carbapenemase producing Enterobacteriaceae (CPE) is clinically needed for fast isolation and proper antibiotics treatment of infected patients. Although modified Hodge test (MHT) is widely used as the standard screening test for CPE, it has lack of sensitivity, specificity, and inavailability to identify particular strain. We compared MHT with another methods: ChromID CARBA and ChromID OXA to verify which test is more suitable for screening of CPE.

**Materials and methods**

We collected 9 identified CPE specimens (two KPCs, one VIM, two NDM-1, one OXA-48, and two OXA-282 strains). We take 10 external quality control specimens (nine KPCs and one VIM-27) from College of American Pathologists. Each specimen was directly inoculated to ChromID CARBA, blood agar plate (BAP) and Mueller-Hinton agar. Then, it was reinoculated to MHT plate and ChromID OXA after 24 hours of incubation. All plates were read after 24 hours of cultivation on the plate after the last inoculation.

**Results**

MHT inoculated from BAP revealed 84% (16/19) of positivity result. It was unable to recovered NDM-1 and OXA-48 isolates. MHT inoculated from MHA was positive on 78% (15/19) of specimens and negative on a KPC (*Klebsiella pneumoniae*) isolate. ChromID OXA test was positive on all three OXA-specimens. ChromID CARBA revealed 94.7% (18/19) of positivity. The negative result of ChromID CARBA was observed on KPC (*Morganella morganii*) specimen.

**Conclusions**

Since ChromID CARBA shows higher sensitivity, ChromID CARBA can be a helpful test for screening of CPE.

## Session: Microbiology

### M4

**Withdrawn**

## Session: Microbiology

### M5

**Withdrawn**

## Session: Microbiology

### M6 The correlation between blood culture positive rate and blood collection volume in a teaching hospital

#### Yao-Shen Tung^1,2,3^, An-Chi Chen^1,2^ , Shen-Min Huang^1,2^ , Yui-Yein Yang ^1,2,3^, Li-Hung Wu^1,3^

##### ^1^Show Chwan Memorial Hospital, Changhua, Taiwan; ^2^Departments of Medical Laboratory, Show Chwan Memorial Hospital, Changhua, Taiwan; ^3^Infection Control Office, Show Chwan Memorial Hospital, Changhua, Taiwan

###### **Correspondence:** Yao-Shen Tung (yaushen51@gmail.com)

**Background**

Blood culture is considered a major method for diagnosis of bacteremia. Blood volume obtained for culture is an important factor in detecting bacteremia. However, the majority of hospitals in Taiwan do not pay attention to the criteria for an ideal volume during collection.

**Material and methods**

During a 1-year study period (1 September 2014 – 31 Aug 2015), there were 18,888 blood culture samples from 26 clinical units outpatient, emergency, intensive care unit and general wards. The BD BACTEC Blood Volume Monitoring system (Becton Dickinson, Sparks, MD) and BACTEC FX were used to identify the total blood volume that was obtained.

**Results**

An average blood volume was 3.80 ml. and the positive rate of blood culture was 10.4%. The emergency room had a lowest mean blood volume (2.20 ml) with a positive blood culture rate of 10.4%. The surgical intensive care unit had a highest mean blood volume (5.70 mL) with a positive blood culture rate 15.6%.

**Conclusions**

In addition to a number of blood culture set, amount of blood taken for blood culture plays an important role for the positive rate. According to the CLSI M47 blood culture guidelines, the recommended blood volume taken from adult is at least 10 mL per bottle. The recommended blood volume from the blood bottle company is 8-10 ml. Adherence to the CLSI recommendation could improve the positive rate, which would result in reduction of antibiotic overuse, reduction of hospital expenditure as well as improvement of quality of care.

## Session: Microbiology

### M7 Set up a process to improve the *C.difficile* diagnosis

#### Chin-cheng Lin^1^, Fu-chieh Chang^2^, Chang-pan Liu^2^

##### ^1^Department of infection, Mackay memorial hospital, Taipei, Taiwan; ^2^Infection control center, Mackay memorial hospital, Taipei, Taiwan

###### **Correspondence:** Fu-chieh Chang (maple0711@gmail.com)

**Background**

*Clostridium difficile* infection (CDI) is a symptomatic infection due to the spore forming bacterium; *Clostridium difficile.* Symptoms include watery diarrhea, fever, nausea, and abdominal pain. CDI spreads by fecal bacterial spores. Spores may contaminate environmental surfaces and further spread by healthcare workers. Risk factors for CDI include antibiotic or proton pump inhibitors use, hospitalization, other health problems, and older age. Diagnosis can be done by stool culture or testing for the bacteria's DNA or toxins.

**Material and methods**

This study was conducted between March and October 2016. A total of 30 stool samples were transferred using 95% EtOH as a transport buffer and were processed by using the chromagar as a selective media. Toxin Screening was performed by using the multiple step EIA kit as. Finally, we used the PCR methods to validate the final result.

**Results**

Before this process, the culture positive ratio was very low (7%) in our hospital. The CDI ratio is only 69/100,000 patients while the CDI ratio of other Medical in Taiwan is more than 100/100,000 patients. After implementation the new diagnostic scheme, the culture positive ratio increased to 26% and the CDI ratio was higher than before. We also performed the GDH EIA test which revealed the similar results to the culture methods.

**Conclusions**

Our study confirmed that the GDH EIA may be an alternative option for diagnosis of CDI. Furthermore, use of 95% EtOH as a transport media would improve the CDI ratio.

## Session: Microbiology

### M8 Laboratory efforts to in-time enhance malaria infection control: a case report

#### Tzu Hao Lien^1^, Jia Hao Chang^2^, Yu Shan Huang^3^, Yi Shun Chen^1^

##### ^1^Laboratory Medicine Department, National Taiwan University Hospital Hsin-Chu Branch, Hsin-chu, Taiwan; ^2^Department of Emergency Medicine, National Taiwan University Hospital Hsin-Chu Branch, Hsin-chu, Taiwan; ^3^Division of Infectious Diseases, National Taiwan University Hospital Hsin-Chu Branch, Hsin-chu, Taiwan

###### **Correspondence:** Yi Shun Chen (lm0718hch@yahoo.com.tw)

**Background**

Malaria, eradicated for over 40 years in Taiwan, is categorized as type 2 mandatory communicable diseases. About 30 to 50 imported malaria cases are still diagnosed each year, with the vast majority in travelers returning from South Asia, Africa and Oceania.

We reported a confirmed malaria case announced total 8 cases in 2015, to CDC. The patient was previously misdiagnosed with common cold in other health care institution, without improvement later, visited our hospital and was eventually diagnosed with imported malaria infection. By this report we hope to share the experience of in-time diagnosis and emphasize its benefit on containment of malaria.

**Material and methods**

A 23-year-old woman with cough and high fever visited our emergency department on Sep. 29, 2015. The doctor, through enquiry of recent travel history, was informed of her 2-week stay in Burkina Faso of Africa 3 weeks before. Besides, laboratory data showed severe thrombocytopenia, jaundice and elevated liver enzymes. Imported malaria infection was highly suspected. The doctor ordered immediate microscopic examination of blood smear, and was notified the observation of many ring forms in red blood cells. Falciparum malaria was diagnosed. Specimens were subsequently collected; according to the guidelines of Taiwan CDC, and referred for further polymerase chain reaction (PCR) testing also provided by CDC laboratory.

**Results**

Positive falciparum malaria result was confirmed by PCR testing.

**Conclusions**

Enquiry of travel history to patients with fever is the key point to accurate diagnosis of malaria infection. Combined with in-time gold standard blood smear examination, effective infection control of malaria and higher quality of health care could be achieved.

## Session: Microbiology

### M9 Anionic thickener in lubricating gel formulation reduce an antimicrobial activity of chlorhexidine gluconate solution

#### Chalermpong Saenjum^1^, Sasithorn Sirilun^1^, Teerapat Ouirungrog^2^, Phisit Ouirungroj^2^

##### ^1^Department of Pharmaceutical Sciences, Faculty of Pharmacy, Chiang Mai University, Chiang Mai, Thailand; ^2^PoseHealthCare Co., Ltd., Kannayao, Bangkok, Thailand

###### **Correspondence:** Chalermpong Saenjum (chalermpong.saenjum@gmail.com)

**Background**

Chlorhexidine gluconate (CHG) is a cationic bis-biquanide biocide with low mammalian toxicity and broad-spectrum antibacterial activity. Positively charge of CHG molecules bind to negatively charge of phospholipid in bacterial cell wall coursing membrane disruption. Incompatible of anionic thickener in lubricating gel formulation can negate the efficacy benefits of CHG. This study was carried out to investigate the in vitro chemical and biological incompatibilities of marketed lubricating gels with CHG solution.

**Materials and methods**

Five marketed lubricating gels containing anionic thickener (Carbomer) and two marketed lubricating gels containing nonionic thickener were collected to investigate for *in vitro* kinetic chemical and biological incompatibilities with 2%w/v CHG in water. The contact time was 15, 30, 45, 60, 90, and 120 seconds.

**Results**

The results demonstrated that, five marketed lubricating gels containing Carbomer reduced the amount of CHG (32.5 – 45.5%), which was measured by reversed-phase HPLC technique and other two marketed lubricating gels containing nonionic thickener maintained the amount of CHG. The antimicrobial activity revealed that 2%w/v CHG solution achieved the 5.69 and 5.45 log_10_ reduction for *Staphylococcus aureus* ATCC25923 and *Escherichia coli* ATCC25922, respectively. The antimicrobial activity was dramatically reduced to the 1.40 – 2.70 log_10_ reduction for *S. aureus* ATCC25923 and 1.02 – 2.11 log_10_ reduction for *E. coli* ATCC25922 after 30 seconds mixed with five marketed lubricating gels containing Carbomer. Two marketed containing nonionic thickener maintained the antimicrobial activity of 2%w/v CHG solution.

**Conclusions**

It can conclude here that, anionic thickeners in lubricating gel formulation may neutralize positively charge and reduce the antibacterial activity of CHG.

## Session: Microbiology

### M10 Pathogenic organisms contamination on mobile phones using by students in clinical microbiological laboratory practice

#### Suwanna Trakulsomboon, Patcharee Prasajak

##### Faculty of Medical Technology, Rangsit University, Pathumthani, Thailand

###### **Correspondence:** Suwanna Trakulsomboon (suwanna.tra@rsu.ac.th)

**Background**

Mobile phone serves as a major reservoir of pathogenic microbes and may act as major carriers for transmission of pathogens from laboratories. The aim of the study is to determine pathogenic bacterial contamination on mobile phones using by medical technology students in clinical microbiology laboratory practices.

**Materials and methods**

Bacterial contamination were isolated and identified from overall surface of 50 mobile phones after touching by medical technology students before and during clinical laboratory microbiology practice.

**Results**

Species of pathogen contaminated on mobile phones used by 54% (27 from 50) of students were identical with the pathogenic organisms that were given for practice in each student. A total of 105 bacterial isolates were found on student’s mobile phones collected before practice. These were *S. epidermidis* 92%, *Bacillus* spp. 90%, MSSA 12%, GNF 10%, *S. saprophyticus* 2%, *Micrococus* spp. 2%, *Enterococcus*spp. 2%. Sample collected during clinical microbiology practice found 122 bacterial isolates These isolates were identified as Bacillus spp. 90%, *S. epidermidis* 88%, MSSA 22%, GNF 16%, *P. vulgaris* 4%, *K. pneumoniae* 4%, *E. cloacea* 4%, *E. aerogenes* 4%, *S. rubidaeae* 4%, *S. saprophyticus* 2%, *S. marcescens* 2%, *P. agglomerans* 2%, *C. freundii* 2%, *S*. Paratyphi A 2%. Significant higher numbers of bacterial isolates (p < 0.05) obtained from student’s mobile phones during clinical microbiology laboratory practice were observed in comparison with those obtained before practice.

**Conclusions**

These results indicated that mobile phone can act as a mobile reservoir for laboratory and hospital infection. Therefore, mobile phone using should be prohibited during clinical laboratory practice. In the additional; mobile phone decontamination and hand washing; should be strictly included as essential tools for laboratory safety and infection control.

## Session: New Technology in Infection Prevention

### NT1 Trial use and evaluation of disinfecting port protectors on central venous catheter in hematology ward in Hong Kong

#### Maryanne W.N. Kwok, Lady S.H. Ng, Lindy M.T. Wong, Lenina S.L. Poon, Mary K.L. Lai, Holly H.S. Cheng, S.K. Fong, Cindy F.Y. Leung

##### Department of Medicine, Queen Elizabeth Hospital, Hong Kong, China

###### **Correspondence:** Maryanne W.N. Kwok (maryanne-kwok@hotmail.com)

**Background**

Intraluminal contamination on the hubs of needless connector (NC) can colonize and migrate along the Central venous catheters (CVCs) and further lead to Catheter-related bloodstream infection (CRBSI), one of the most deadly and costly healthcare-associated complication. Current practice is to rub the connectors with 2% chlorhexidine (CHG) for 15 seconds, unfortunately the compliance rate is 60% for nurse and 40% for intern. This trial is to compare the effectiveness and compliance of using a new sterile 70% isopropyl alcohol impregnated disinfecting port protector (Curos, 3 M).

**Materials and methods**

From 1st June - 31st Aug 2016 (3-months period), all hematologic patients with Peripherally Inserted Central Catheter (PICC), Hickman catheter and Hemostar catheter were using Disinfecting Port Protector on NC, following manufacturer’s instruction without 15 seconds 2% CHG rubbing.

**Results**

No significant difference in CRBSI rate comparing to the existing practice (rub).Protocol compliance rate for nurses and intern increased from 60% (rub) to 90% (protector) and 40% to 85% respectively during the first week and maintained >95% thereafter. Most nurses responded that the new protocol is preferred which can ensure “always-cleaned & protected” hub reduce contamination risk of CVC blood cultures and time saving.

**Conclusions**

Based on the evidence shown, we implemented change in our practice using disinfecting port protectors.

## Session: New Technology in Infection Prevention

### NT2 Preemptive therapy for the prevention of cytomegalovirus disease in high-risk (D+/R-) kidney transplant recipients

#### Jumpei Hasegawa^1,2^, Hiroki Shirakawa^3^, Sachiko Wakai^1^, Makiko Mieno^4^, Shuji Hatakeyama^5,6^

##### ^1^Department of Nephrology, Tokyo Metropolitan Health and Medical Treatment Corporation Okubo Hospital, Kabukicho, Shinjuku-ku, Tokyo, Japan; ^2^Department of Urology, Tokyo Women’s Medical University, Kawadacho, Shinjuku-ku, Tokyo, Japan; ^3^Department of Urology, Tokyo Metropolitan Health and Medical Treatment Corporation Okubo Hospital, Kabukicho, Shinjuku-ku, Tokyo, Japan; ^4^Department of Medical Informatics, Center for Information, Jichi Medical University, Yakushiji, Shimotsuke-shi, Tochigi, Japan; ^5^Division of General Internal Medicine/Division of Infectious Diseases, Jichi Medical University Hospital, Yakushiji, Shimotsuke-shi, Tochigi, Japan; ^6^Department of Internal Medicine, Tokyo Metropolitan Health and Medical Treatment Corporation Okubo Hospital, Kabukicho, Shinjuku-ku, Tokyo, Japan

###### **Correspondence:** Shuji Hatakeyama (shatake-tky@umin.ac.jp)

**Background**

Universal prophylaxis and preemptive therapy are used to prevent cytomegalovirus (CMV) disease after transplantation. However, the optimal strategy has not been identified. This study aimed to evaluate optimal prevention strategies, particularly in high-risk recipients (donor-positive/recipient-negative [D+/R-] CMV serostatus pairs).

**Materials and methods**

We compared CMV infection/disease incidence and patient and graft outcomes in high-risk (D+/R-) and intermediate-risk (D+/R+ or D-/R+) kidney transplant recipients managed by preemptive therapy.

**Results**

We studied 118 recipients (21 high-risk, 97 intermediate-risk; median follow-up >1,100 days). Asymptomatic CMV infection and CMV disease developed significantly more frequently in high-risk than in intermediate-risk patients (38.1% vs. 16.5%, *P* = 0.04; 28.6% vs. 3.1%, *P* < 0.01, respectively). A higher peak CMV antigenemia titer (112 vs. 19 cells, *P* < 0.01) and a longer duration of antigenemia (28 vs. 16 days, *P* = 0.02) were observed among high-risk patients. Among high-risk patients, all CMV infection and CMV disease cases developed within the first 3 months and 9 months, respectively, after transplantation. Survival analysis showed no significant difference in mortality rates (log-rank *P* = 0.63) and graft loss rates (log-rank *P* = 0.50) between the groups. Graft rejection occurred more frequently among high-risk patients, but the difference was not significant (log-rank *P* = 0.24). Graft rejection was significantly more common in patients who developed CMV infection/disease than in those who did not (log-rank *P* < 0.0001), regardless of CMV serostatus.

**Conclusions**

Further studies comparing universal prophylaxis and preemptive therapy, particularly in high-risk patients, are required to identify the optimal strategy to reduce graft rejection rates.

## Session: New Technology in Infection Prevention

### NT3 Development of risk management guideline for infectious disease outbreaks during disasters

#### Mariko Tateishi, Mutsuko Mihashi, Yuka Sato

##### Kurume University School of Nursing, Kurme, Fukuoka, Japan

###### **Correspondence:** Mariko Tateishi (tateishi_mariko@med.kurume-u.ac.jp)

**Background**

Japan is a natural disaster-prone archipelago. However few guidelines for public health activities have focused on infectious disease outbreaks during disasters. We have created the risk management guideline for infectious diseases during disasters to develop a comprehensive support system for prevention of infectious disease outbreaks in disasters.

**Materials and methods**

Interviews were conducted with fifteen nurses who worked in any disasters occurred since the Great Hanshin-Awaji Earthquake in 1995. The interview data as well as published documents were analyzed. Factors influencing infectious disease outbreaks were extracted as categories by six researchers. Furthermore, measures were extracted while universalizing these measures.

**Results**

Following categories were extracted: “Restroom”, ”Water management”, “Hygiene products”, “Hygiene behavior to be clean” “Environment”, “Food hygiene”, and “Situations of infectious diseases”. There were 38 measures in “Restroom”, 31 measures in ”Water management”, 36 measures in “Hygiene products”, 30 measures in “Hygiene behavior to be clean”, 28 measures in “Environment”, 39 measures in “Food hygiene”, 52 measures in “Situations of infectious diseases”.

**Conclusions**

Measures could be visualized as a risk management guideline for infectious diseases outbreaks. This guideline is regarded as useful for preparing future disasters. However, it is necessary to consider that measures vary according to what kind of, where, and when disasters occur as the guideline was extracted from disaster experiences in Japan.

## Session: New Technology in Infection Prevention

### NT4 Development of novel formulation against antibiotic-resistance microbes

#### Chalermpong Saenjum^1^, Manu Deeudom^2^, Prasit Tharavichitkul^2^, Teerapat Ouirungrog^3^, Phisit Ouirungroj^2^

##### ^1^Department of Pharmaceutical Sciences, Faculty of Pharmacy, Chiang Mai University, Chiang Mai, Thailand; ^2^Department of Microbioloigy, Faculty of Medicine, Chiang Mai University, Chiang Mai, Thailand; ^3^Pose Health Care Co., Ltd., Kannayao, Bangkok, Thailand

###### **Correspondence:** Chalermpong Saenjum (chalermpong.saenjum@gmail.com)

**Background**

The rapid emergence of resistant bacteria is occurring worldwide. The prevalence of antibiotic resistance among the aforementioned bacteria has been increasing. This study was carried out to develop novel formulation against antibiotic-resistance microbes using for cross-transmission and cross-contamination control.

**Materials and methods**

The novel formulation was developed using quaternary ammonium compounds as major active ingredient and synergistic with three minor active ingredients. The antimicrobial activity against antibiotic-resistance microbes, methicillin-resistant *S. aureus* ATCC 25923, vancomycin-resistant enterococci, *A. baumannii*, *P. aeruginosa* ATCC 27853, ESBL-producing *E. coli, K. pneumoniae, P. mirabilis, Salmonella enteritidis*, *B. subtilis*, *S. maltophilia*, *C. perfringen*, *C.neoformans* **,** *T. rubrum, C. difficile*, Multidrug-resistant *M. tuberculosis* (MDR-TB), *M. tuberculosis* H37Rv (reference strain),*M. avium* (nontuberculous mycobacteria; NTM), was also investigated.

**Results**

The results indicated that, the novel formulation in the concentration of 0.1% exhibited the antimicrobial activity against methicillin-resistant *S. aureus* ATCC 25923, vancomycin-resistant enterococci, *A. baumannii*, *P. aeruginosa* ATCC 27853, *ESBL-producing E. coli, K. pneumoniae, Salmonella enteritidis*, *B. subtilis*, *S. maltophilia*, *C. perfringen*, *C.neoformans* **,** *T. rubrum,* and *C. difficile* in 1 minute of exposure time and 5 minutes for *P. mirabilis*. Moreover, the concentration of 0.5% exhibited the antimicrobial activity against Multidrug-resistant *M. tuberculosis* (MDR-TB), *M. tuberculosis* H37Rv (reference strain), *M. avium* (nontuberculous mycobacteria; NTM) in 1 minute. The oral acute toxicity in Wistar rat in both sexes was greater than 5,000 mg/kg body weight.

**Conclusions**

It can conclude here that, the novel formulation exhibited the potent antimicrobial activity against antibiotic-resistance microbes and also showed the safety in animal study.

## Session: Outbreak Investigations

### OI1 *Ralstonia picketti* outbreak in a hemodialysis centre – the role of microbiology laboratory

#### Terrence Chinniah, Jackson Tan, Kavitha Prabu, Sartaj Alam, Aung Kyaw Wynn, Rashidah Ahmad, Amalina Sidek, Dg Azizah Samsuddin, Noraini Ajis, Aliyah Ahmad, Susylawathi Magon, Boon Chu

##### Raja Isteri Pengiran Anak Saleha Hospital, Bandar Seri Begawan, Brunei Muara, Brunei Darussalam

###### **Correspondence:** Terrence Chinniah (trctrc64@gmail.com)

**Background**

Infections in hemodialysis (HD) patients are often associated with vascular access. Certain organisms like *Ralstonia picketti* may infect vascular access and subsequently contaminate and linger in the water treatment and reprocessing system of dialysis centers and trigger outbreaks, through direct contamination of water and dialysis filters. This study was performed to determine the source of outbreak of this organism.

**Materials and methods**

Blood cultures are done on hemodialysis patients with fever and isolated organisms are identified by VITEK 2, VITEKMS, API20E and API20NE. Antibiotic sensitivity is performed according to Clinical Laboratory Standard Institute guidelines 2016.

**Results**

Microbiology laboratory isolated *Ralstonia picketti* in blood cultures from four patients who underwent hemodialysis on the same day at a same center, similar antibiogram of all these isolates lead to a suspicion of an outbreak. Repeated blood cultures and the jugular venous catheter tip from one of the patient grew the same pathogen leading to the suspicion of this patient as the index case of the outbreak. Routine cleaning, disinfection and sterilization of the water treatment system did not stop further patients from experiencing rigors and infection with the same pathogen. No new patient was identified after the cessation of dialysis reprocessing in the center. This confirms *Ralstonia pickettii* has infected the dialysis reprocessing system from the index case that was dialyzing via a jugular catheter, and subsequently, infecting other patients in the center.

**Conclusions**

*Ralstonia pickettii* can be encountered in dialysis facility through contamination of water treatment and reprocessing system, resulting in outbreaks. Early identification of pathogen is essential to prevent escalation of outbreaks in dialysis centers.

## Session: Outbreak Investigations

### OI2 Survey on the epidemiologic characteristics of carbapenem- *resistant Klebsiella pneumoniae* in a tertiary hospital in Beijing

#### Jiqiu Kuang^1^, Yan Gao^1^, Shoujun Wang^1^, Yunxiao Hao^1^, Rong Liu^1^, Dongmei Li^1^, Hui Wang^2^

##### ^1^Infection Control and Prevention Dept, Peking University People's Hospital, Beijing, China; ^2^Clinical Laboratory, Peking University People's Hospital, Beijing, China

###### **Correspondence:** Jiqiu Kuang (phkuang@163.com)

**Background**

*Klebsiella pneumoniae* are important pathogen in healthcare-associated infections. Therefore, Carbapenem-resistant *Klebsiella pneumonia* (CRKP) causes infections with high mortality rates, which the ratio of increases in the recent years. This study aimed to investigate the epidemiological characteristics of CRKP in a tertiary hospital in Beijing and seek for control strategy.

**Materials and methods**

Retrospective survey and temporal-spatial distribution analysis were used to CRKP cases newly diagnosed from Jan to April, 2016 and pulsed-field gel electrophoresis (PFGE) was used to analyze the homology of CRKP isolates.

**Results**

In the 25 CRKP cases, the median duration of hospitalization within 90 days before detected was 27 days (5-90 days), the duration of using Carbapenems was a median of 12 days (0-43 days), 10 cases had history inpatient of other hospitals before admission. The major sources of specimens with CRKP positive were blood, respiratory tract and urinary tract, with a positive rate of 32%, 32% and 20%, respectively. Homology test was performed in 20 CRKP cases from 11 wards. MLST analysis showed that 19 cases were all ST11 subtype producing KPC-2 enzyme; PFGE results showed that 8 cases were type 1, 10 cases were type 2, with 2 unclassified. Temporal-spatial distribution of type 1 and type 2 cases suggested that there might be contact transmission between cases.

**Conclusions**

It is necessary to establish regional multidrug-resistant organisms monitoring and tracking network and information should be shared between departments and hospitals during patient referral, which was the key strategy for CRKP prevention and control.

## Session: Outbreak Investigations

### OI3 Lesson learnt from a norovirus outbreak in developmental disabilities unit

#### Ng Po Yan (carolpoyan@gmail.com)

##### Caritas Medical Centre, Hospital Authority, Hong Kong, China

**Background**

Acute gastroenteritis due to Norovirus is highly infectious and always causes outbreaks in settings where people have close contact. It affects people of all age groups and tends to be more common during winter. Developmental Disabilities Unit (DDU) in Caritas Medical Centre (CMC) resided around 100 children with severe disability who need high dependency of care. A Norovirus outbreak was confirmed in January of 2016, which triggered by an infected kid who infected by family members after home leave.

**Materials and methods**

We conducted a comprehensive health surveillance to detect any risk of cross infection and early detection for isolation.

**Results**

Three children resided in the same dormitory developed gastroenteritis symptoms within 2 days and laboratory investigation revealed norovirus infection. Followed by active case finding, 4 more children in the same dormitory confirmed to have norovirus infection. All infected children with signs and symptoms were cohorted. Infection control measures enhanced, including hand washing instead of alcoholic hand rubbing after handling excreta; children from the infected dormitory were retained from school; disposable utensils were used for artificial tube feeding; differentiate clean and dirty team for procedures. Furthermore, 7 children on the same floor of DDU were confirmed to have Norovirus in their stool specimen. Three was no new case after 2 incubation periods. The outbreak was declared over afterward. All isolation measures discontinued after 2 weeks.

**Conclusions**

A comprehensive health surveillance for all child who returned from home, information including the health condition of close relatives or care givers in order to have early detection and isolation of infectious diseases.

## Session: Outbreak Investigations

### OI4 The efficacy of PCR-based ORF typing methods of MRSA in nosocomial infection control in NICU

#### Hisanori Nishio, Hitomi Mori, Yoshiko Morokuma, Takaaki Yamada, Makiko Kiyosuke, Sachie Yasunaga, Kazuhiro Toyoda, Nobuyuki Shimono

##### Kyushu University Hospital, Fukuoka, Japan

###### **Correspondence:** Hisanori Nishio (hisanorinishio@gmail.com)

**Background**

It is difficult to control nosocomial infection of methicillin-resistant *Staphylococcus aureus* (MRSA) in Neonatal intensive care unit (NICU). Recently, a new epidemiological analysis of MRSA, polymerase chain reaction (PCR)-based Open Reading Frame (ORF) Typing (POT) methods, was developed. We controlled MRSA outbreak in NICU by this POT methods.

**Materials and methods**

We investigated nasal colonization of MRSA in neonatal patients once a week. From May 2014 to May 2016, in all the isolated MRSA strains, we performed genotyping of MRSA by this POT method. Based on these results, we took measures against nosocomial infection control of MRSA.

**Results**

MRSA outbreak occurred sporadically in our NICU. By this POT method, we clarified nosocomial transmission of MRSA in NICU and educated NICU staffs to perform proper and thorough hand hygiene with alcohol rub. As a result, usage amount of alcohol hand rub increased twofold and MRSA outbreak terminated earlier than before.

**Conclusions**

The education based on genotyping could lead to improvement of hand hygiene compliance. Our results suggest that this POT method is effective in nosocomial infection control of MRSA in NICU.

## Session: Outbreak Investigations

### OI5 New algorithm for clustering of spa typing data

#### Dmitriy Babenko^1^, Anar Turmuhambetova^1^, Antonella Cheşcă^2^, Mark A Toleman^3^

##### ^1^Karaganda State Medical University, Karaganda, Kazakhstan; ^2^Transilvania University of Braşov, Braşov, Romania; ^3^Cardiff University, Cardiff, Wales, UK

###### **Correspondence:** Dmitriy Babenko (streampost@gmail.com)

**Background**

Staphylococcal protein A (spa) typing is one of the modern subspecies typing of *S. aureus.* It is considered as a fast, discriminating, and very reproducible methods. However, the current approaches using commercial automated spa typing is limited by high cost. We present the algorithm for cluster analysis based on spa typing.

**Materials and methods**

*S. aureus* genomes from GenBank were used to determine MLST and spa types in silico (Seqsphere). Grouping of STs into clonal complexes (CC) was performed using eBURST (≤1 allele difference in CC). Spa types were clustered into groups based on genetic distance calculated by pairwise alignment using Needle-Wunsch algorithm with some modifications (R statistics) and according to EDSI model (with 1 value for each event). Concordance between spa CC and MLST CC was estimated with adj.Rand index (R statistics). Spa with <4 repeats were excluded from the analysis.

**Results**

Of 4976 *S. aureus* genomes allowed to determine, they were assigned to 218 different ST and 507 spa types. After removing spa types with ≤3 repeats, 4934 genomes underwent the further analysis. There were 32 multilocus sequence typing (MLST) CC (4919 isolates) and 15 singletons based on eBURST algorithm and 29 spa CC (4901 isolates) and 33 singletons using our approach. The concordance between spa clusters and MLST CC was 90.4% (95% CI; 89.2-91.5%).

**Conclusions**

Our study showed that spa typing is useful in analysis of clonal structure. It had high agreement with MLST CC.

## Session: Outbreak Investigations

### OI6 Why does MLST scheme for *S.aureus* provide lower discriminatory power?

#### Dmitriy Babenko^1^, Anar Turmuhambetova^1^, Antonella Cheşcă^2^, Mark A Toleman^3^

##### ^1^Karaganda State Medical University, Karaganda, Kazakhstan; ^2^Transilvania University of Braşov,Braşov, Romania; ^3^Cardiff University, Cardiff, Wales, UK

###### **Correspondence:** Dmitriy Babenko (streampost@gmail.com)

**Background**

Multilocus sequence typing (MLST) is an unambiguous technique for characterizing microorganisms based on the sequencing of housekeeping genes providing standardized strain nomenclature. However, this approach usually have lower resolution in comparison with other typing methods. One of the possible reason is slowly evolving housekeeping genes. We investigate the reasons of lower resolution power of MLST scheme for *S.aureus*.

**Methods**

*S.aureus* genomes were used to extract MLST types. MLST profile (allelesin 7 loci; arcC, aroE, glpF, gmk, pta, tpi, and yqiL) was used to calculate genetic distance and further single-linkage clustering for grouping into MLST clonal complex with *ape* in R package. Simpson and adjusted Rand indices were calculated with *untb* and *vegclust* R packages.

**Results**

Of 6532 *S.aureus* genomes, there were 242 different strain types by MLST. Discriminatory power of MLST typing was 84.7% (95% CI 84.3-85.2%). The levels of resolution ability of each locus ranged from 70 to 80%, except for *glpF* locus which had 31%discriminating ability. High concordance between loci of MLST scheme was observed between *aroE, gmk, pta, tpi* ranged from 86.5% to 92.9%. MLST with 4 loci (*arcC, glpF, pta, yqiL*), previously excluded the loci with high interlocus concordance (*aroE, gmk and tpi*), demonstrated the similar discriminatory ability (83.2%, 95%CI 82.8-83.7%) in comparison with classical MLST scheme. Concordance between MLST with 7 loci and MLST with 4 loci was 0.945 (95%CI 0.94-0.95) at type level and 0.95 (95%CI 0.95-0.96) at clonal level.

**Conclusions**

Loci with high interlocus concordance included in MLST scheme for *S.aureus* typing did not provide a significant increase in the discriminatory power of the methods as a whole.

## Session: Outbreak Investigations

### OI7 Comparison of seven genotyping methods for *Staphylococcus aureus*: in SILICO study

#### Dmitriy Babenko^1,^ Anar Turmuhambetova^1^, Ilya Azizov^2^, Antonella Cheşcă^3^, Mark A Toleman^4^

##### ^1^Karaganda State Medical University, Karaganda, Kazakhstan; ^2^Institute of Antimicrobial Chemotherapy, Smolensk, Russia; ^3^Transilvania University of Braşov, Braşov, Romania; ^4^Cardiff University, Cardiff, Wales, UK

###### **Correspondence:** Dmitriy Babenko (streampost@gmail.com)

**Background**

Typing methods is known to play important role in epidemiological investigations and by now, many typing methods using different principles have been developed to differentiate *Staphylococcus aureus* isolates.In silico comparison seven different typing methods (MLST, MVLST, spa, MLVA, cgMLST, wgMLST and panGenome) in terms of discriminatory power and level of concordance.

**Materials and methods**

MLST, MVLST, spa, MLVA, cgMLST, wgMLST, panGenome types were determined on *S.aureus* genomes (n = 4976) in silico using *FindMyFriends* and *rBLAST* R packages R and USEARCH tool. Clustering types into groups was performed using parameters: maximal difference 1 allele for MLST, MVLST and MLVA; 1 event according *EDSI* model for spa and 10% cut-off for cgMLST, wgMLST and panGenome. Discriminatory power (Simpson index Diversity-SID) and concordance (adjusted Rand-AR) were calculated with *untb* and *vegclust* R packages.

**Results**

PanGenome, wgMLST and cgMLST demonstrated ultimate discriminatory power (SID = 1.0). MLVA, MVLST and spa showed lower resolution ability with 97.7%(95%CI;97.7-98%), 91.9%(95%CI;91.4-92.5%) and 90.7%(95%CI;90.2-91.3%), respectively. MLST had the lowest Simpson index of 0.854(95%CI;0.848-0.859). The highest concordance (84.5%) was between wgMLST and cgMLST on type level. In other cases, the agreement was no more 40%. On cluster level, there were better congruence – 95.6% in wgMLST-cgMLST pair, 90.4% for spa-MLST, 88% between panGenome and wgMLST/cgMLST, from 82.6% to 85.6% between MVLST and cgMLST/wgMLST/MLST. The lowest concordance were between MLVA and other typing methods (less than 61%).

**Conclusions**

CgMLST, wgMLST and panGenome typing showed ultimate discriminatory power. Concordance between different typing methods were poor at type level, excluding wgMLST-cgMLST. On cluster level, the agreement was better achieving 85% and more.

## Session: Outbreak Investigations

### OI8 Genetic variation of *S.aureus* isolates within and between MLST types

#### Lyudmila L Akhmaltdinova^1^, Anar Turmuhambetova^1^, Antonella Cheşcă^2^, Dmitriy Babenko^1^

##### ^1^Karaganda State Medical University, Karaganda, Kazakhstan; ^2^Transilvania University of Braşov, Braşov, Romania

###### **Correspondence:** Lyudmila L Akhmaltdinova (immunol.lab@gmail.com)

**Background**

Multilocus sequence typing (MLST) as a genotyping methods has been extensively used for study of evolution and population dynamics of *S.aureus*. The knowledge about genetic distances of genomes within and between MLST types estimated on wgMLST can be useful for understanding the population structure.We explored the variation within and between MLST types of global collection of *S.aureus* using wgMLST typing.

**Materials and methods**

6645 *S.aureus* genomes from GenBank were analyzed with SeqSphere + to determine MLST and wgMLST types. Data with more than 10% missing values were excluded from analysis. Genetic distance was calculated with Nei formula (NeiM., 1972) using wgMLST data with 2103 loci.

**Results**

Eight MLST types including 5591 *S.aureus* were determined: ST5 (n = 1673), ST8 (n = 1392), ST22 (n = 987), ST398 (n = 632), ST105 (n = 534), ST36 (n = 161), ST239 (n = 128) and ST609 (n = 84). The mean of variation within MLST type was 2.9% (95%CI;0.9-5.0). In some STs, such as ST239, ST5, ST8, the genetic distance reaches 15.8%, 17.4% and 21%, respectively. In general, the difference between isolates from different STs ranged 65-92%. However, in some cases, the difference between *S. aureus* from different MLST types were much lower – 28% (ST239-ST609), 14% (ST8-ST239), 11% (ST8-ST609) and 5% (ST5-ST105).

**Conclusions**

Analysis of global collection *S.aureus* isolated from different sources and places demonstrated high genome difference between MLST types differing by 6 alleles. While genetic distances within and between closest STs might be overlapped what makes impossible to find appropriate cut-off for *S.aureus* clustering using wgMLST data to differentiate MLST with SLV adequately, at least in some cases.

## Session: Outbreak Investigations

### OI9 Benchmarking of carbapenem-resistant *Acinetobacter baumannii* outbreaks: a tertiary hospital in a developing country study

#### Mark Albert Magsakay^1^, Angelo Macatibag^1^, Maria Fe Tayzon^1^, Jeannica Kriselle Lerios^2^

##### ^1^Hospital Infection Control and Epidemiology Center, The Medical City, Quezon, Metro Manila, Philippines; ^2^Department of Cardiology, The Medical City, Quezon, Metro Manila, Philippines

###### **Correspondence:** Mark Albert Magsakay (tmc07102@gmail.com)

**Background**

An outbreak of multi-drug resistant pathogen is considered a major hospital problem. It may lead to temporarily cessation on operation and even permanent disqualification to serve. *Acinetobacter* spp*.* is one of the rising multidrug-resistant organisms known to cause significant hospital-associated outbreaks. This is a summary of Carbapenem-resistant *Acinetobacter baumannii* (CRAB) outbreaks experienced by The Medical City (TMC) during 2011 and 2016.

**Material and methods**

In 2011, TMC – Hospital Infection Control and Epidemiology Center implemented including antimicrobial stewardship, contact precautions, hand hygiene and environmental decolonization. A multi-disciplinary approach to stop the outbreak was performed by the team which include immediate cohorting of patients, surveillance cultures among patients, and an indwelling infection preventionist in the intensive care unit to conduct audit and feedback. Further, the approach from the first CRAB outbreak was adopted in 2016.

**Results**

A sizable outbreak of CRAB in TMC was most likely caused by cross-transmission through hand carriage of healthcare workers. Outbreaks can be controlled through initiation of comprehensive infection control measures including Antibiotic Stewardship and thorough hand hygiene. 2011 CRAB outbreak was controlled after 126 days, while 2016 event was contained in 9 days from the date of detection of outbreak.

**Conclusions**

A comprehensive infection control approach is effective in curtailing outbreaks secondary to *A.baumannii*. Commitment of healthcare workers are still of prime importance in implementation of infection control practices for TMC to prevent outbreak.

## Session: Outbreak Investigations

### OI10 The SNP-typing of nosocomial strains of *A. baumannii* isolated in 5 tertiary hospitals of The Central Kazakhstan

#### Ilya Azizov^1^, Alyona Lavrineko^2^, Dmitry Babenko^2^, Eugene Sheck^1^, Mikhail Edelstein^1^

##### ^1^Institute of Antimicrobial Chemotherapy, Smolensk, Russian Federation; ^2^Karagamnda State Medical University, Karagamnda, Kazakhstan

###### **Correspondence:** Ilya Azizov (ilya.azizov@antibiotic.ru)

**Background**

*Acinetobacter baumannii* are common bacterial nosocomial pathogen caused large number of hospital infections. Different methods are using for molecular typing *A. baumannii* strains (PFGE, MLVA, MLST, whole-genome MLST) but only part of used regions of DNA have high discriminate rate. Present methods are characterized as difficult for routine using and expensive. Simple SNP-based methods of clonal typing are previously described. The study is to examine SNP-based study of clonal distribution of nosocomial strains isolated in tertiary hospitals of the Central Kazakhstan.

**Materials and methods**

Ninety-nine nosocomial carbapenem resistant strains of *A. baumannii* in 5 tertiary hospitals of the Central Kazakhstan have been collected during 2015-2016 in frame of National Multicenter Study. The isolating were standard bacteriological methods. The identification had been done by MALDI-TOF mass spectrometry and detection species specific carbapenemases OXA-51. OXA-23 carabapenemases had been detected by using comercial kit for PCR. The sensitivity to antimicrobials was done by dilution methods according EUCAST criteria. The SNP typing was done in all obtained isolates.

**Results**

All of studied srtains were producer of OXA-23 carbapenemases and resistant to beta-lactams, aminoglycosides, tetracyclines, chloramphenicol, ftorquinolones. The sensitivity was detected only for colistine (MIC_50_ = 1 mg/l). More 99% of studied strains were in second International Clonal Group (ICL2).

**Conclusions**

The population of nosocomial *A. baumannii* strains in The Central Kazakhstan was characterizing as monoclonal and present The Second International clonal Group. This clonal group had not registered in Kazakhstan before 2015.

## Session: Outbreak Investigations

### OI11 An outbreak investigation of type A influenza among healthcare providers in a regional hospital in Southern Taiwan

#### Tzu-Yin Liu, Lih-Yue Li, Chiung-Wen Chan, Hui-Chuan Pan, Tun-chieh Chen

##### Kaohsiung Municipal Ta-Tung Hospital, Kaohsiung, Cianjin, Taiwan

###### **Correspondence:** Tzu-Yin Liu (ned740206@yahoo.com.tw)

**Background**

In June 2015, three nurses had Flu-like symptoms in our medical, surgical and neurological wards within couple days.

**Material and methods**

We started outbreak investigation in medical, surgical and neurological wards.

**Results**

We identified 9 nurses with Influenza A infections. After investigation, we found that compliance of wearing surgical masks while caring patients among healthcare providers is adequate but the compliance of hand hygiene is inadequate. Furthermore, they neglected their Flu-like symptoms and did to practice good respiratory hygiene or cough etiquette. Although these healthcare providers are able to follow the infection control standards while taking care a patient, they fail to follow the rule while contacting with staff members. These resulted in outbreak among healthcare providers. The outbreak was successfully controlled by introducing fever surveillance, advocating respiratory hygiene and cough etiquette and reinforcing environmental clearness.

**Conclusions**

Of these findings, enhanced hand hygiene and associated infection control strategies should be implemented to effectively reduce the transmission of type A influenza.

## Session: Outbreak Investigations

### OI12 The development of guideline for outbreak control of varicella in in-patient department of Somdet Chaopraya institute of psychiatry

#### Wipa Vanishakije, Warisra Jaikampun

##### Somdetchaopraya institute of psychiatry, Klongsan, Bangkok, Thailand

###### **Correspondence:** Wipa Vanishakije (ihappy_t@yahoo.com)

**Background**

This research and development was a cohort study from 2013 to 2015. Firstly, varicella disease had impacts on hospital administration: 1) unable the ward having patient(s) who had varicella infections to admit new patients, 2) infected non-immunized hospital staff. Secondly, the disease interfered normal hospital services: 1) the affected ward was unable to admit new patients for 4 months because it contained the infected patients 2) an affected ward increased amount of new patients in others under limited resources of staff and facilities. In consequences, it increased patients’ complains on hospital services. This study aimed to develop a guideline for controlling an outbreak of chickenpox in in-patient department and to evaluate the use of this guideline.

**Materials and methods**

This study was separated into 3stages: 1) isolating patients having varicella infections, prohibiting admission of new patients in the units and evaluated contact persons by testing for varicella immunoglobulin G (IgG), 2) isolating patients having varicella infections but allowed admission of new patients into the units, evaluated 275 contact persons by testing for varicella IgG and used protective isolation room for patients who were at risk for acquiring the disease and 3)isolating patients having varicella infections, allowed admission of new patients, evaluated 143 contact persons by testing for varicella IgG, used protective isolation room for high-risk patients and vaccinated individuals who had negative results for varicella IgG.

**Results**

No additional patients having varicella infection were found even though the hospital did not stop accepting new patients.

**Conclusions**

The study is in progress to create screening criteria to identify patients having varicella infection before hospitalization.

## Session: Outbreak Investigations

### OI13 Investigation and control of a carbapenem-resisitant *Klebsiella pneumoniae* outbreak in a subacute respiratory care center

#### Pei-Chen Cheng^1^, Huey-Jen Huang^1^, Shu-Ju Huang^1^, Yu-Huan Huang^1^, Su-YinLi^1^, Su-Fang Yu^1^, Jian-Feng Li^2^, Yu-Ping Wu^3^, Yuan-Ti Lee^2^

##### ^1^Infection Control Team, Chung Shan Medical University Hospital, Taichung, Taiwan; ^2^Infectious Diseases Division, Chung Shan Medical University Hospital, Taichung, Taiwan; ^3^Department of Nursing Medicine, Chung Shan Medical University Hospital, Taichung, Taiwan

###### Corresponding author: pejan0322@gmail.com

**Background**

From September 16 to October 10, 2016, six cases of Carbapenem-resisitant *Klebsiella pneumoniae* (CRKP) related healthcare-associated infections, that were catheter-associated urinary tract infections, were identified and investigated in a 16-bed subacute respiratory care center (RCC) of a medical center.

**Materials and methods**

Infection control personnel had identified this event through routine surveillance, and implemented some infection control measures immediately. The interventions included the following points: (1) cohort care for the infected patients was introduced; (2) daily environmental cleaning with 5000 ppm bleach was implemented; (3) the other patients were followed with active screening for CRKP colonization; (4) environment and healthcare workers were screened for CRKP colonization; (5) healthcare professionals were educated for contact precautions and hand hygiene.

**Results**

Among 138 swab cultures from RCC environment, CRKP were isolated from a bedside table and a physiological monitor of RCC05 and an infusion pump of RCC15. We collected 12 samples of HCWs’ hands and found CRKP from one nurse, who was a key nursing staff of RCC05 patient. Anal swab screening in contact patients showed two of CRKP colonization that contacted withRCC05 and RCC15 patients.

**Conclusions**

The study showed that poor compliance of hand hygiene among staff and wards’ environment contamination was the reasons for this outbreak. The implementation of hand hygiene and ward environment cleaning and disinfection along with other infection control interventions were very important. However, this incident has not yet been ceased. Ongoing monitoring and enforcement of infection control measures are still required.

## Session: Outbreak Investigations

### OI14 Analysis on patients with diarrhea caused by *Clostridium difficile* infection in metropolitan hospitals of some area in Taiwan

#### Chiao-Hui Lin, Ping-Chin Chang

##### Chi Mei Medical Center, Liouying, Tainan, Taiwan

###### **Correspondence:** Chiao-Hui Lin (susan891224@gmail.com)

**Background**

Incidence of *Clostridium difficile* infection (CDI) in Taiwan has not been widely studied, because the yield of culture based diagnostic test was low and the clinical characteristics of CDI had limited studies in the past. However many studies found the incidence of CDI increased in recent years. This study aimed to analysis on patients *Clostridium difficile* infection to prevent cross infection.

**Materials and methods**

The following data was retrospectively reviewed; clinical evaluation, operational definition/diagnosis, diagnostic test using rapid molecular detection of toxigenic *C. difficile*. The medical records was reviewed and collected in case assessment form, which included underlying chronic diseases, past medical history, history of hospitalization, recent operation, past and current medication, particularly antibiotics use, and nutritional status.

**Results**

One-year incidence in our hospital was 18.1%. Of the patients with CDI, 4 patients underwent chemotherapy, 1 patient had intestinal surgery, 2 patients were on hemodialysis, and 1 patients had diabetes mellitus and long-term use of steroids. All patients were hospitalized within 90 days, and received antibiotics. The medication duration was 1-2 weeks. The hospitalization time was 8-14 days. The mean age of patients was 60 years old.

**Conclusions**

The development of rapid and reliable diagnostic tools is important to identify CDI. Effective treatment, prevention strategies are needed to control this rising infection.

## Session: Outbreak Investigations

### OI15 *Mycoplasma pneumoniae* outbreak in a medical record department at a tertiary care hospital, northern Thailand

#### Samatanet Tariyo, Sangwan Paengta, Ratchanee Wongsaen, Suttsiphan Thanompan

##### Infection Control and Prevention Unit, Nakornping Hospital, ChaingMai, Thailand

###### **Correspondence:** Samatanet Tariyo (Keng_khonsuy@hotmail.com)

**Background**

In September 2015, the infection control nurse at a tertiary care hospital, northern Thailand was notified of an influenza-like illness (ILI) outbreak among the health care workers (HCWs) in a medical record department. We conducted an outbreak investigation to determine the etiologic agent, identify additional cases, and implement control measures.

**Materials and methods**

An outbreak investigation of respiratory infections among HCWs in a medical record department was promptly initiated. HCWs with influenza ILI were enrolled during August -September 2015. Respiratory swabs were collected fromthe HCWs with ILI andwere tested by real-time reverse transcriptase polymerase chain reaction (RT-PCR) for respiratory pathogens.

**Results**

*Mycoplasma pneumoniae* is a significant cause of respiratory disease in this outbreak. The infection control team alerted HCWs to the outbreak and recommended prevention measures.

**Conclusions**

*Mycoplasma pneumoniae* should be considered as a possible cause of respiratory disease in HCWs in outbreak setting. Morbidity from outbreaks in hospital might be minimized if facilities closely monitor for respiratory disease clusters, promptly report outbreaks to infection control team, prioritize diagnostic testing in outbreak situations, and timely implement strict infection control measures to halt transmission.

## Session: Outbreak Investigations

### OI16 Surgical site infections following coronary artery bypass graft: a 3-year surveillance data on prevalence and microbiology

#### Sumawadee Skuntaniyom, Kumthorn Malathum, Suchada Sukkra

##### Ramathibodi Hospital, Bangkok, Thailand

###### **Correspondence:** Sumawadee Skuntaniyom (su_meaw@hotmail.com)

**Background**

Surgical site infections (SSI) following coronary artery bypass graft (CABG) procedures is a devastating complication with significant morbidity, mortality, and extra-cost. This study aims to describe the incidence of SSI and causative pathogens following CABG surgery in a university hospital during 2013-2015.

**Materials and methods**

Hospital records and post-discharge surveillance data between 2013 and 2015 were reviewed. SSI was diagnosed according to the US-CDC’s surveillance definitions.

**Results**

There were 682 patients undergone CABG surgery. Of these, 54 (7.92%) had SSI; 23 at sternal sites and 31 at leg harvest sites; 52 classified as superficial SSI and 2 as deep SSI. This prevalence is higher than what have been reported. In this study, proportion of Gram-positive and Gram-negative bacteria was 11% each. Of 54 cases with SSI, 3 cases had polymicrobial infection. Gram-positive bacteria included coagulase negative staphylococci 18.2%, *Enterococcus* spp*.* 13.6%, and *Staphylococcus aureus* 9.1%. Gram-negative bacteria were *Escherichia coli* 13.6%, *Klebsiella pneumoniae* 4.5%, *Enterobacter cloacae* 4.5% and *Pseudomonas aeruginosa* 27.3%. There was no difference in the prevalence of infection among surgeons. One-third of patients received prophylactic antibiotic very closely to operation, (≤30 minutes) and it was discontinued mostly within 48 hours after surgery, while 8.1% of all patients received antibiotic for longer than 48 hours.

**Conclusions**

Post CABG SSI was high in this study, probably related to inappropriate administration of prophylactic antibiotic. In-depth study is needed to delineate other risk factors which could affect the implementation of preventive strategies.

## Session: Occupational Health/Vaccine

### O1 Safety of 4-dose vial presentation of the pneumococcal non-typeable *Haemophilus influenzae* protein D-conjugate vaccine in infants: a phase III randomized study

#### Khalequ Zaman^1^, Sheikh Farzana Zaman^1^, Farzana Zaman^1^, Asma Aziz^1^, Sayeed-Bin Faisal^1^, Magali Traskine^2^, Javier Ruiz-Guiñazú^2^, Dorota Borys^2^

##### ^1^International Centre for Diarrheal Disease Research, Dhaka, Bangladesh; ^2^GlaxoSmithKline (GSK) Vaccines, Wavre, Belgium

###### **Correspondence:** Khalequ Zaman (kzaman@icddrb.org)

**Background**

The 4-dose vial (with preservative) of pneumococcal non-typeable *Haemophilus influenzae* protein D-conjugate vaccine (PHiD-CV, GSK Vaccines) was developed to improve logistics of and adherence to immunization programmes. The aim of this study was to assess reactogenicity and safety of the investigational PHiD-CV 4-dose vial presentation in infants.

**Materials and methods**

In this phase III, mono-centre, observer-blind study (NCT02447432) conducted in Bangladesh, 6-10-week-old infants, randomized 1:1, received primary vaccination with either PHiD-CV 4-dose (4-dose group) or PHiD-CV 1-dose vial (preservative-free, 1-dose group) at 6/10/18 weeks of age. DTPw-HBV/Hib and polio vaccines were (co)-administered at 6/10 weeks of age. Solicited and unsolicited adverse events (AEs) within 4 and 31 days post-vaccination, respectively, and serious AEs (SAEs) throughout the study were assessed.

**Results**

The total vaccinated cohort comprised 160 infants in each group. The most commonly reported PHiD-CV injection site and general solicited AEs were pain (after 13.1% and 13.4% of doses in 4-dose and 1-dose groups, respectively) and irritability/fussiness (after 23.3% and 26.6% of doses, respectively). The overall/dose incidences of other injection site (redness and swelling; 2.4%-5.9%) and general AEs (drowsiness, loss of appetite and fever [axillary temperature ≥37.5 °C]; 10.0%-15.6%) were within similar ranges between groups. Unsolicited AEs were reported after 2.1% and 3.7% of doses, and SAEs were reported for 4 and 9 infants in 4-dose and 1-dose groups, respectively, none was considered as vaccination-related. Two infants died (sepsis, 4-dose; septic shock, 1-dose).

**Conclusions**

PHiD-CV 4-dosevial presentation (with preservative) had an acceptable reactogenicity and safety profile, similar to PHiD-CV 1-dose vial (preservative-free) in infants.

**Acknowledgements**

GlaxoSmithKline Biologicals SA.

## Session: Occupational Health/Vaccine

### O2 Immunogenicity of 4-dose vial presentation of pneumococcal non-typeable *Haemophilus influenzae* protein D-conjugate vaccine in infants: a phase III randomized study

#### Khalequ Zaman^1^, Sheikh Farzana Zaman^1^, Farzana Zaman^1^, Asma Aziz^1^, Sayeed-Bin Faisal^1^, Magali Traskine^2^, Javier Ruiz-Guiñazú^2^, Dorota Borys^2^

##### ^1^International Centre for Diarrheal Disease Research, Dhaka, Bangladesh; ^2^GSK Vaccines, Wavre, Belgium

###### **Correspondence:** Khalequ Zaman (kzaman@icddrb.org)

**Background**

To facilitate multi-dose use, GSK Vaccines developed the pneumococcal non-typeable *Haemophilus influenzae* protein D-conjugate vaccine (PHiD-CV) 4-dose vial, which contains preservative. The aim of this study was to demonstrate non-inferiority of the immunogenicity of investigational PHiD-CV 4-dose versus licensed 1-dose vial presentation in infants.

**Materials and methods**

In this phase III, observer-blind study (NCT02447432) conducted in Bangladesh, 6-10-week-old infants, randomized 1:1, received PHiD-CV primary vaccination at ages 6/10/18 weeks with either 4-dose (4-dose group) or 1-dosevial (preservative-free, 1-dose group). DTPw-HBV/Hib and polio vaccines were (co)-administered at ages 6/10 weeks. Immune responses (antibodies against pneumococcal serotypes [22F-ELISA] and opsonophagocytic activity [OPA]; anti-protein D antibodies [ELISA]) were measured. Non-inferiority of PHiD-CV 4-dose versus 1-dose for each vaccine pneumococcal serotype (VT) and vaccine-related serotype 19A (confirmatory objectives) in terms of antibody geometric mean concentration (GMC) ratios was assessed.

**Results**

Of 320 vaccinees, 154 (4-dose) and 146 (1-dose) were included in the according-to-protocol cohort for immunogenicity. Non-inferiority criterion (upper limit of 2-sided 95% confidence interval of the antibody GMC ratios [1-dose/4-dose] <2-fold) was met for each VT and 19A. For each VT, ≥97.9% of infants in each group had antibody concentrations ≥0.2 μg/mL, except for 6B (84.4%, 4-dose; 84.9%, 1-dose) and 23F (89.0%, 4-dose; 94.5%, 1-dose); for 19A, ≥80.1% of infants in both groups. OPA for each VT and 19A, and anti-protein D responses were within similar ranges between groups.

**Conclusions**

Immunogenicity of PHiD-CV 4-dose vial (with preservative) was non-inferior to 1-dose vial (preservative-free) in terms of antibody GMC ratios for each VT and 19A post-primary vaccination in infants.

**Acknowledgements**

GlaxoSmithKline Biologicals S.A.

## Session: Occupational Health/Vaccine

### O3 Multi-interventional strategy to reduce percutaneous injuries in a non-profit private hospital

#### Wendy Wai Yee Lam^1^, May Chow^2^, Lucy Choy^2^, Joseph Kam^1^

##### ^1^Infection Control Unit, Canossa Hospital (Caritas), Hong Kong, China; ^2^Canossa Hospital (Caritas), Hong Kong, China

###### **Correspondence:** Wendy Wai Yee Lam (wylam@canossahospital.org.hk)

**Background**

Percutaneous injuries resulting from contaminated sharp devices among healthcare workers (HCWs) is a major concern of staff safety in hospitals, with increased risk HIV, hepatitis B and C infections. Percutaneous injuries were at top five risks in our hospital in 2010-2013, with >60% of injuries related to operating theatre (OT). We aimed to study the impact of a multi-interventional strategy on incidence of percutaneous injuries at a non-profit private care hospital in Hong Kong.

**Materials and methods**

Canossa Hospital (Caritas) is a 148-bed non-profit private care hospital treating approximately 10,000 patients yearly with 400 HCWs. Between 9-10 incidents of percutaneous injury occurred annually in 2010-2013. From 2013, a multi-interventional strategy was introduced to reduce incidence of percutaneous injuries, including: identifying risks of bloodborne infections exposure, formulation of ward policy guidelines, staff education and training to create safe workplace environment, enhancement of reporting system, individual counseling by infection control nurse, provision of safety-engineered sharps devices (e.g. needle counter in OTs), verbal-visual reminder (meetings, posters). Compliance audits were performed in OTs after implementation.

**Results**

Number of percutaneous injuries declined from between 9-10 incidents in 2010-2013 to 5 and 3 incidents in 2014 and 2015 respectively. The proportion of injured staff showed an initial decrease of 50% (p = 0.1, chi-square test) in 2014 and then significant reduction of 70% (p < 0.05) in 2015.

**Conclusions**

We achieved reduction in percutaneous injuries using multi-interventional strategy. Implementation of guideline formulation, education, individual counselling after incident, verbal-visual reminders, safety devices and performance audits led to an improvement in hospital staff safety.

## Session: Occupational Health/Vaccine

### O4 Epidemiology of sharp injury in a Malaysian teaching hospital

#### Sharifah Azura Salleh^1^, Razila Yacob^2^, Siti Rokiah Yusof^2^, Nordiah Awang Jalil^1^

##### ^1^Department of Medical Microbiology and Immunology, Universiti Kebangsaan Malaysia Medical Centre, Cheras, Kuala Lumpur, Malaysia; ^2^Infection Control Unit, Universiti Kebangsaan Malaysia Medical Centre, Cheras, Kuala Lumpur, Malaysia

###### **Correspondence:** Sharifah Azura Salleh (drazura@ppukm.ukm.edu.my)

**Background**

Accidental sharp injury with the risk of contracting blood-borne infection is a major occupational hazard for healthcare workers (HCWs). The aim of this study is to look at the epidemiology of sharp injury in Universiti Kebangsaan Malaysia Medical Centre (UKMMC) between 2013 and 2016.

**Materials and methods**

All sharp injury incidents were included. Data was obtained from the record of HCWs with sharp injury incident reported to Infection Control Unit UKMMC between 2013 and August 2016.

**Results**

Total of 312 incidence of sharp injury reported over the 4 years study period. The highest incidence was in 2013 with 97 reported cases, followed by 81 cases in 2015, 79 cases in 2014 and 55 in 2016. The top 3 locations that reported sharp injury during the 4 years were Medical Wards (69 cases), General Operating Theatre (30 cases) and Surgical Ward (30 cases). During this study period, 19.6% cases occurred sharp injury in relation to blood taking procedure (n = 61). Waste collection is the second commonest situation for sharp injury at 13.8% of reports (n = 43) and 35 cases reported during disposing sharps into sharp bins (11.2%). Doctors reported the highest incident with 126 cases (40.4%) while nurses reported 62 cases (19.9%) and various groups of HCWs constitutes the remaining 39.7%.

**Conclusions**

All HCWs are at risk of sharp injury however certain groups are at higher risk. The rate of sharp injury is persistently high despite the ongoing education program for HCWs in our institution.

## Session: Occupational Health/Vaccine

### O5 Effects of increasing flu vaccination coverage on staff sick leaves: incremental cost benefit analysis

#### Jose Paulo Flor, Nicolo Andrei Añonuevo, Marko Bautista, V Jay De Roxas, Justine Vergara, Maria Lourdes Millan, Marion Kwek, Jose Lito Acuin

##### Asian Hospital and Medical Center, Muntinlupa, National Capital Region, Philippines

###### **Correspondence:** Nicolo Andrei Añonuevo (naanonuevo@asianhospital.com)

**Background**

Influenza can be a serious disease, since health care workers may care for or live with people at high risk for influenza-related complications. This study aimed to increase staff vaccination rates in 2015 and to know whether the additional cost is serving its worth, which shows the incremental cost - benefit ratio of increasing vaccine coverage.

**Materials and methods**

In 2015, the team conducted unit by unit information campaigns through floor to floor dissemination of information about free vaccination in the Employee Health Clinic, and lastly “I am Asian” jackets were provided to staff who underwent the vaccination.

**Results**

In 2014, the team vaccinated 820 employees (73% vaccination rate) among 1117 total number of employees. While in 2015, vaccination of 1030 employees (88% vaccination rate) among 1174 total number of employees was conducted. In 2016, 1379 employees (89% vaccination rate) among 1544 total number of employees were vaccinated. This represented an additional 427 employees who got vaccinated in 2016. The number of sick leave days decreased by 40%, from 96 days in 2014 to 58 days in 2015. We assumed conservatively that each sick leave day cost the hospital 468.00 pesos.

**Conclusions**

Annual influenza vaccination incurs at most a net cost of 77 centavos or 770 pesos for the current workforce of 1,000 staff members. This is a cost beneficial intervention which must be continued. For this reason, in 2016, the team used the quadrivalent vaccine which cost 650 pesos. This will determine the cost benefit ratio of this enhanced program in 2017.

## Session: Occupational Health/Vaccine

### O6 Cost implications and control of adult varicella among healthcare workers in a tertiary hospital in Manila Doctors Hospital, Philippines

#### Aisa Jensen Lee, Melecia A. Velmonte, Silverose Ann A. Bacolcol, Allan Alde, Keitleen Chavez, Arlene Joy Esteban

##### Infection Prevention and Control Office, Manila Doctors Hospital, Manila, National Capital Region, Philippines

###### **Correspondence:** Silverose Ann A. Bacolcol (silveroseann@yahoo.com)

**Background**

In 2015, there have 17 documented cases of adult varicella zoster virus (VZV) among healthcare workers in a tertiary hospital in the Philippines. The purpose of this study was to determine the cost implications of the occurrence of VZV among healthcare workers and control the transmission of the virus.

**Materials and methods**

From 2015 to 2016, the Infection Prevention and Control Office conducted an outbreak investigation on the cases of VZV among healthcare workers. This involved contact tracing and review of medical history of the healthcare workers. A survey on the immunization history of all healthcare workers (HCW) was done. Outbreak control was also recommended through vaccination of all exposed that have no active immunity on the virus. The calculated cost of the treatment for HCWs was done.

**Results**

Result of the investigation revealed that 18% of the cases are cross transmission from one healthcare worker to his other co-workers. Most of the healthcare workers who are on the prodromal stage of the virus still reported to work. The cost of the treatment per personnel who have VZV was calculated at 23,400 PHP (479 USD) while the cost of the VZV is at 4,000 PHP (83 USD). Among all HCWs 68% have no active immunity to VZV while 60% have no vaccination to VZV.

**Conclusions**

There is a need to strengthen VZV information campaign among HCW. Mandatory immunization of all susceptible staff to VZV must be recommended and barriers to vaccination uptake among HCWs must be studied. Reiteration of the sick leave policy must be emphasized.

## Session: Occupational Health/Vaccine

### O7 Nursing experience with one postoperative oral cancer patient using transdisciplinary care model

#### Ching-I Ting (alice3030301@yahoo.com.tw)

##### Chi-mei medical center intensive care unit, Tainan, Taiwan

**Background**

Oral cancer patients are susceptible to postoperative structural defects of tissues such as in the orofacial region and jaw bones, which cause changes in the facial appearance, defects of physical functions such as dysphagia, slurred speech and eating difficulties and consequent negative impacts on quality of life. Each of these patients, therefore, represents a highly complicated case with multiple problems and requires collaboration between a wider range of specialty departments on case discussion and provision of care to reduce their postoperative complications and improve their quality of life.

**Materials and methods**

The healthcare provided to the patient and the family through the transdisciplinary care model included: (1) alleviation of wound pain for the patient; (2) planning of training sessions for rehab from dysphagia; and (3) continuing coaching for the patient’s mother on caregiving for the patients at home.

**Results**

This paper, based on the reason for and the definition of transdisciplinary care, presents for the reference of clinical practitioners leaders of medical institutions a specifically addressed strategy of evidence-based transdisciplinary practice, integrating the care plans suggested by all the teams and providing healthcare and rehab plan that reduced and improved the patient’s wound pain and dysphasia.

**Conclusions**

The evidence-based clinical practice of transdisciplinary care is a key trend of care in medical environments. Using transdisciplinary evidence-based care as the foundation helps healthcare professionals across disciplines to understand and respect the spirit of collaborative care and obtain best treatment strategies through communications, thereby providing good quality of care.

## Session: Occupational Health/Vaccine

### O8 Infection control on air ambulance

#### Sunisa Dissayasriroj (sunisa@aircharterthailand.com)

##### Medical Wings, Siam Land Flying, Bangkok, Thailand

**Background**

The introduction of ASEAN Economic Community (AEC) and Thai government’s policy in creating an international center for medical treatment, there has been a significant increase in number of patients travelling into Thailand for treatments. Patients are travelling on commercial aircrafts as well as air ambulance when their conditions are urgent or critical. Air ambulance is a well-recognized method of transportation by the insurance company which Medical Wings has been serving patient from around the world since 1999. At Medical Wings, we focus on Safety, quality care and infection control. On occasion, we receive patient from facility which is not able to adequately assess infectious disease so our medical team are the first group to meet patient and preparation is the utmost important in a successful mission. We aimed to prevent the exposure of patients, visitors and healthcare workers to communicable or infectious diseases by stressing maintenance of sound habits in personal hygiene and individual responsibility in infection control, monitoring and investigating potentially harmful infectious exposures, providing care to personnel for work-related illnesses, identifying infection risks related to employment and instituting appropriate preventive measures.

**Materials and methods**

1.Employee health, 2.Post-handover surveillance, 3.Isolation Precautions, 4.Disinfection & sterilization, 5.Patient care, 6.Education & training, 7.Environmental control, 8. Research

**Results**

1. Patient’s infection rate 0%, 2.HAIs rate 0%, 3. Results of swab test and microbial air sampler: pre & post deep cleaning effective more than 99.9%, 4. Employee satisfaction rate more than 90%

**Conclusions**

We focus directly to prevent infection. Educating medical and non-medical caregivers are our key strategy.

## Session: Prevention of MDROs

### PM1 Successful control of carbapenem-resistant Enterobacteriaceae outbreaks

#### Terrence Rohan Chinniah, Kavitha Prabu, Rashidah Ahmad, Susylawathi Magon, Jauharatud DiniSuhaimi, Aizzuddin Mirasin, Nurul Morni, Boon Chu, Azizah Samsuddin, Aliyah Ahmad, Amalina Sidek, Noraini Ajis, Amalina AbuBakar, Amanie Shafiee, Julaini Safar

##### Raja Isteri Pengiran Anak Saleha Hospital, Bandar Seri Begawan, Brunei Muara, Brunei Darussalam

###### **Correspondence:** Terrence Rohan Chinniah (trctrc64@gmail.com)

**Background**

Infection with Carbapenem-resistant Enterobacteriaceae (CRE) is increasing worldwide leading to high morbidity and mortality. No CRE cases were detected in Brunei till April 2013. This study aimed to control the outbreak as soon as it’s detected by microbiology laboratory.

**Materials and methods**

All specimens at microbiology laboratory are cultured according to routine laboratory methods and identified by VITEK 2, VITEK MS, API 20E and API 20NE. Antibiotic susceptibility test is performed according to Clinical Laboratory Standard Institute (CLSI) guidelines. Enterobacteriaceae showing resistant to carbepenems, namely ertapenem, imipenem and meropenem, were rechecked by VITEK 2 XL, Kirby-bauer method and Minimum Inhibitory Concentration by E-strips. If it still remain resistance to carbapenem, clinicians and infection control team was alerted immediately by email and phone. All these were found to be carbapenemase producing CRE, by Modified-Hodge test recommended by CLSI.

**Results**

One case of CRE was identified in April 2013. No case was detected during the year of 2014. From March 2015 till October 2015, sixteen cases of CRE were confirmed. Another small outbreak of four cases was noted from January 2016 till March 2016. No cases were identified since then.

**Conclusions**

Following identification of one case in April 2013 barrier nursing and isolation were re-visited to seal spread from this case. Outbreak of 15 cases in 2015 was mainly among critical care patients. Due to vigorous infection control measures this outbreak was control. Similarly the smaller outbreak of 5 cases in 2016 was also controlled by vigorous infection control practices. This experience of ours underlines the importance of early detection of CRE and strict infection control measures.

## Session: Prevention of MDROs

### PM2 A continuous quality improvement (CQI) program of prevention and control of multiple drugs resistant organisms (MDROs) infections in a respiratory medical ward

#### Ng Po Yan, Leung Annie, Fung Yuk Ling, Lau Edna, Luk Kristine

##### Infection Control Team, Caritas Medical Centre, Hospital Authority, Hong Kong, China

###### **Correspondence:** Ng Po Yan (carolpoyan@gmail.com)

**Background**

There was methicillin-resistant *Staphylococcus aureus* (MRSA) endemic and multidrug resistant *Acinetobacter baumannii* (MDRAB) increased in a male respiratory medical ward since 2014. This program aimed to control the spread of MDROs by bundle of infection control enhancement.

**Materials and methods**

A pilot CQI program was conducted in the respiratory ward since December 2015 by enhancing the quality care for 5 basic care procedures: hand hygiene, catheter care, nasogastric tube care, incontinence care and perianal care, promoting hand hygiene by training senior nurses in the ward as advocators to monitor the compliance of different disciplinary staff and improving the decontamination of respiratory equipment by standardizing as high level disinfection or replaced by disposable equipment.

**Results**

All ward staffs had been audited on 5 basic care procedures in 1Q 2016. Total 19 senior nurses in the ward were trained to be the hand hygiene auditor. Hand hygiene compliance rate was improved to over 90%. Respiratory equipment was either disposable or undergone high level disinfection at the end of program. Disinfection of respiratory equipment in ward was eliminated. Both MDRAB and MRSA has been decreased. MRSA hospital acquired infection (HAI) rate decreased from 1.3 in September 2015 to 0.6 in March 2016. There had no new MDRAB clinical cases from November 2015 to March 2016. The successful CQI program had been implemented to other medical wards in our hospital.

**Conclusions**

MDROs increase the mortality of patients and it is a great challenge to patient’s safety and prolonged hospital stay. Good compliance in basic infection control measures is a key factor to battle with MDROs in hospital.

## Session: Prevention of MDROs

### PM3 Comprehensive strategy to reduce MRSA by dedicated certified nurse in infection control (CNIC) involvement at community hospital in Japan

#### Satoshi Shinomiya, Kumiko Yamamoto, Kayoko Kjiwara, Mitsuhiro Yamaguchi

##### Minoh City Hospital, Minoh, Japan

###### **Correspondence:** Satoshi Shinomiya (fxdl822@gmail.com)

**Background**

In Japan the government has decided to pay incentives for infection control (IC) interventions from 2012. Detection of methicillin-resistant *Staphylococcus aureus* (MRSA) is useful for evaluating efficacy of IC interventions in healthcare facilities. Our study would like to evaluate if comprehensive strategy of IC interventions are effective via monitoring MRSA rates.

**Materials and methods**

We investigated MRSA incidence per 1,000 patient-days from April 2007 to March 2016. Comprehensive strategy of IC interventions we’ve taken during study period were 1) full-time dedicated CNIC involvement since 2009, 2) routine reporting of multidrug-resistant organisms (MDROs) to CNIC, and 3) Personnel Protective Equipment (PPE) holder and portable hand sanitizer introduction.

**Results**

The mean detection rate of 1.29 was statistically decreased to 0.71 after the implementation of the IC interventions.

**Conclusions**

The detection of healthcare-associated MRSA has reduced after implementing comprehensive strategy. Key element of comprehensive strategy is an intervention by dedicated CNIC.

## Session: Prevention of MDROs

### PM4

**Withdrawn**

## Session: Prevention of MDROs

### PM5 Are topical nasal antiseptics effective in reducing methicillin-resistant *Staphylococcus aureus* (MRSA) colonization?

#### Angela Chow, Grace Tin, Wei Zhang, Pei-Yun Hon, Bee-Fong Poh, Kalisvar Marimuthu, Brenda Ang

##### Tan Tock Seng Hospital, Singapore, Singapore

###### **Correspondence:** Angela Chow (Angela_Chow@ttsh.com.sg)

**Background**

Methicillin-resistant *Staphylococcus aureus* (MRSA) is a growing clinical problem in intermediate-care facilities. Nasal carriage could be a source of nosocomial transmission, and decolonization could reduce institutional MRSA prevalence. We evaluated the effect of octenidine nasal gel, coupled with universal chlorhexidine baths, in reducing MRSA prevalence.

**Materials and methods**

We conducted a quasi-experimental before-after study, screening all inpatients for MRSA in a rehabilitation facility in Singapore, in June-2014, July-2015, and July-2016. Nasal, axilla, and groin swabs were cultured on selective chromogenic agar. Universal chlorhexidine baths were implemented in 2014-2015. Octenidine nasal gel for MRSA-colonizers was added in 2016. Multivariable logistic regression models were constructed to assess for differences in MRSA prevalence.

**Results**

A total of 257 patients (97 in 2014, 77 in 2015, 83 in 2016) were screened. MRSA prevalence was 33.0% in 2014, 39.0% in 2015, and 19.3% in 2016 (P = 0.020). Groin colonization decreased from 21.7% (2014) and 28.6% (2015), to 14.5% (2016) (P = 0.094). Nasal colonization decreased from 17.5% (2014) and 21.8% (2015), to 6.0% (2016) (P = 0.020). Median length of stay (LOS) was longer in 2016 (20 days) compared to 2014 (14 days, P = 0.043) and 2015 (13 days, P = 0.012), although age and gender distributions were similar. After adjusting for age, gender, and LOS, MRSA colonization was significantly higher in 2014 (OR 2.69, 95%CI 1.23-5.89) and 2015 (OR 4.00, 95%CI 1.77-9.02) than 2016.

**Conclusions**

Topical nasal octenidine, coupled with universal chlorhexidine baths, can reduce MRSA colonization prevalence in intermediate-care facilities. Larger studies should be conducted to validate the findings and assess for applicability in other settings.

## Session: Prevention of MDROs

### PM6 The influence of disease burden for patients infected with multidrug-resistant pathogens

#### Ming-Chin Chan^1^, Chih-Chien Wang^2^

##### ^1^Infection Control Office, Tri-Service General Hospital, National Defense Medical Center, Taipei, Taiwan; ^2^Department of Pediatrics, Tri-Service General Hospital, National Defense Medical Center, Taipei, Taiwan

###### **Correspondence:** Chih-Chien Wang (jmj621115@gmail.com)

**Background**

Since the use of antibiotics, drug-resistant pathogens were inevitably appeared. It is undoubtedly a huge challenge to clinical practices. Infections with multidrug-resistant Pathogens would result in higher mortality rate and extra medical expenses.

**Materials and methods**

This research collected and analyzed healthcare-associated bloodstream infection by multi-drug resistant pathogens including *Acinetobacter baumannii*,* Pseudomonas aeruginosa*, Vancomycin-resistant enterococci (VRE), *Staphylococcus aureus* and *E. coli* in Tri-Service General Hospital between 2011 and 2014. The cases were collected by infection control nurses. All the information and the expenditure of ICUs or wards were analyzed and compared.

**Results**

The results showed the highest mortality was VRE, about 80% and 57% of mortality rate in ICUs and wards, respectively. The longest days from admission to infection was *Staphylococcus aureus*, about 55.4 and 74.5 days in ICU and ward, respectively. Moreover, pathogen that has the longest days from infection to outpatient or expired was *E. coli*, with the average of 59.6 and 41.6 days in ICUs and wards, respectively. As for the expenditure, patients infected with *E. coli* in ICUs, and patients infected with VRE in wards cost the most expenses, which was about 15,333 and 5,000 US dollars, respectively.

**Conclusions**

The expenditure for nosocomial infections with multi-drug resistant pathogens in ICUs is higher than in wards, especially infected with *E. coli*, the other four pathogens caused about 6,333-10,000 US dollars in ICUs. In wards, it is about 3,667-4,667 US dollars infected with VRE, *Staphylococcus aureus* and *E. coli*. The antibiotics cost is the highest when infected with VRE.

## Session: Prevention of MDROs

### PM7 The effectiveness of body wash employing 2% chlorhexidine to reduce healthcare-associated infections in a medical ICU of a medical center

#### Shu-Ju Huang^1^, Huey-Jen Huang^1^, Su-Fang Yu^1^, Huan-Yu Huang^1^, Pei-Chen Cheng^1^, Jian-Feng Li^1,2^, Yuan-Ti Lee^1,2^, Chiung-Ling Lai^3^, Min-Chi Lu^4^

##### ^1^Infection Control Team, Chung Shan Medical University Hospital, Taichung, Taiwan; ^2^Infectious Diseases Division, Chung Shan Medical University Hospital, Taichung, Taiwan; ^3^Department of Nursing Medicine, Chung Shan Medical University Hospital, Taichung, Taiwan; ^4^Infectious Diseases Division, China Medical University Hospital, Taichung, Taiwan

###### **Correspondence:** Shu-Ju Huang (nunu20101202@gmail.com)

**Background**

In recent literature, the use of 2% chlorhexidine gluconate (2% CHG) for skin disinfection via bathing could reduce bacterial colonization. Thereby, healthcare-associated infection (HAI) and multidrug resistant organisms (MDROs) were successfully reduced. In the Medical Intensive Care Unit (ICU) of a Medical Center in Central Taiwan, the density of HAI was 6.6% in 2013 and 28.6% of the isolated pathogens were MDRO. Therefore, 2% CHG body wipe was employed since Apr 2014.

**Materials and methods**

The periods of Apr 2013 - Mar 2014 and Apr 2014 - Mar 2015 were designated as stages of pre-implementation (pIMP) and bundle implementation (bIMP), respectively. For pIMP, daily shower gel bath was used. For bIMP, (1) 2% CHG towel was used to wipe whole body after routine soap bath. Besides, (2) infection control measures, including hand hygiene, sterile techniques, isolation, environmental cleaning and disinfection, and (3) antibiotic stewardship were strictly applied.

**Results**

At bIMP, the HAI density was down to 3.6% from 6.1% of pIMP. Similarly, MDROs rate decreased from 32.1% to 10.3%, Carbapenem-resistant *Acinetobacter baumannii* from 66.9% to 44.2%, Carbapenem-resistant *Pseudomonas aeruginosa* from 14.9% to 13.9%, MRSA from 34.9% to 15.1%, and VRE from 47.7% to 30.0%. However, carbapenem-resistant *Klebsiella pneumoniae* (CRE) increasing from 1.4% to 4.1%. The rise in CRE rate was attributed to the active surveillance of VRE and CRE for patients from long-term care facilities (LTCF) and respiratory wards since Aug 2013.

**Conclusions**

MDROs are prevalent in ICU and LTCF, and contribute a lot to HAI, infection control measures should be implemented cautiously. In this study, 2% CHG wipe was effective in reducing HAI and MDRO rates, and provided a simple strategy and resulted in the improved quality of daily ICU care.

## Session: Prevention of MDROs

### PM8 SHIP BE Program for multidrug-resistant *Acinetobacter baumannii* control in a crowded tertiary care hospital in southern Thailand

#### Sajeerat Kosol, Wantana Sakolwirat, Patchanee Paepong, Sawalee Jansanga, Pattarin Jaisamoot, Nuttha Thongnuanual, Chittima Srithong

##### Suratthani Hospital, Suratthani, Thailand

###### **Correspondence:** Sajeerat Kosol (bew.sajee@gmail.com)

**Background**

Suratthani Hospital is a tertiary care center providing care to patients in Southern Thailand with 880 beds and nearly 100% bed occupancy year round. The most common multidrug-resistant (MDR) bacteria causing infections in the hospital is *Acinetobacter baumannii*. Carbapenem-resistant *Enterobacteriaceae* have also been detected more frequently.

**Materials and methods**

The SHIP BE (S = Staff education, H = Hand Hygiene, I = Isolation/ Information, P = Personal Protective Equipment, B = Bathing with chlorhexidine gluconate (CHG), E = Environment) program was implemented between December 2015 and May 2016 as an effort to control such organisms among patients in general medical wards and medical intensive care units.

**Results**

Seventy nurses were enrolled in the educational program. After education, healthcare workers significantly gained more knowledge (Pre-intervention score 8.9/15 vs. Post-intervention score 10.4/15; *p* < .001). Compliance to isolation precautions increased significantly from 51.6 to95.5% (*p* < .001). For examples, hand hygiene compliance increased from 49.7% to 85.9%, wearing protective barriers increased from 58.6% to 91.9% and dedicated patient care items increased from 54.9% to 98.8%. Correctly performed environmental cleaning increased from 54.9 to 99%. We also use 2% chlorhexidine gluconate bathing frequently for patients (0% to 91.7%). The prevalence of MDROs decreased from 10.4% to 6.8%.

**Conclusions**

The SHIP BE program was effective in reducing the prevalence of MDROs although our hospital was very crowded.

## Session: Prevention of MDROs

### PM9 Successful control of XDR-*Acinetobacter baumannii* in a critical care unit

#### Somporn Somsakul, Kumthorn Malathum, Sutima Plongpunth

##### Ramathibodi Hospital, Bangkok, Thailand

###### **Correspondence:** Somporn Somsakul (rasom_aod@hotmail.com)

**Background**

Extensively drug-resistant *Acinetobacter baumannii* (XDR-Ab) is a growing problem in acute care medical facilities worldwide. The Intermediate Medical Intensive Care Unit (IMU) was one of the areas with highest prevalence of infection caused by this organism in our hospital, 16.47 episodes/1,000 patient-days during October-December 2015. This study aimed to reduce the incidence of XDR-Ab in IMU.

**Materials and methods**

This study was performed between October 1st, 2015 and March 31st, 2016. The IMU had 20 beds, 2 were single-bed isolation room but the remaining was in a common hallway divided into three 6-bed sections. Space between each bed was only 90 cm. A total of 396 patients were observed. After the first three months to determine the baseline prevalence, we implemented the control measures including cohorting patients with XDR-Ab in the same area, encouraging hand hygiene by providing alcohol hand rub solution at each bed and common areas in the ward, mandatory isolation gown and gloves for caring of patient with XDR-Ab, improving environmental cleaning technique and frequency.

**Results**

The prevalence of XDR-Ab was gradually decreased, from 16.47 episodes /1,000 patient-days in the first phase to 3.98 episodes /1,000 patient-days in the second phase.

**Conclusions**

This study suggests that cohorting along with stringent infection prevention technique was effective in controlling spread of drug-resistant organisms and it might be an option for the crowded patient care areas.

## Session: Prevention of MDROs

### PM10 Adherence of healthcare workers to guidelines for contact precautions in a tertiary care hospital

#### Mukkapon Punpop, Porntip Malathum, Kumthorn Malathum

##### Faculty of Medicine Ramathibodi Hospital, Bangkok, Thailand

###### **Correspondence:** Mukkapon Punpop (st_ac124@hotmail.com)

**Background**

The Medical Semi-intensive Care Unit (MSCU) at Ramathibodi Hospital provides care for 20 patients who shared a common hall with 0.9 meter space between beds. The prevalence of multidrug-resistant organisms is highest in the hospital. Contact precautions have been implemented in the past five years to control such organisms. This study aimed to explore adherence of health care workers (HCWs) and their opinions toward guidelines for contact precautions.

**Materials and methods**

Survey using questionnaires and direct, blinded, random observation was performed during regular work shifts from April to May 2015. Descriptive statistics were used to analyze the data.

**Results**

The adherence rate to gloving was highest, followed by gowning and hand hygiene (81%, 56% and 51% respectively). Hand hygiene was performed more after than before giving care to patients. Although they wore gowns and gloves before patient care, HCWs immediately removed gown and gloves infrequently afterward. Forty percent of the HCWs adhered to the guidelines in every step, whereas 14% did not adhere at all. Nurses adhered most to the guidelines (72%). HCWs agreed with the guidelines but were unable to adhere to the guidelines at every step because of the crowded environment and perceived work overload. Results of this study suggested that adherence to the guidelines for contact precautions needs more cooperation among HCWs and barriers to the adherence should be minimized to prevent widespread multi-drug resistant organisms, and thereby improving the quality of care.

**Conclusions**

The gloves compliance rate was highest than gown and hand hygiene. Overall compliance rate 40%. The study finding needs to be concerned by healthcare workers for improving the quality of care.

## Session: Prevention of MDROs

### PM11 Using the development and empowerment program followed role and competency of advanced practice nurse in extreme drug resistance organisms-infected patients caring in medical and surgical departments of Nakornping Hospital

#### Sutthiphan Thanomphan, Ratchanee Wongsaen, Kulada Peautiwat

##### Nakornping Hospital, Chiang Mai, Thailand

###### **Correspondence:** Sutthiphan Thanomphan (kaynine@hotmail.co.th)

**Background**

An Extreme Drug Resistance organisms (XDR) outbreak investigation at NKP Hospital in 2014 revealed that the hospital had 2 risk factors, which were inappropriate number of nurses and substandard infection prevention and control (IC) practices. The purpose of this developmental research was to develop a patient care model for XDR in medical and surgical departments, and to reduce the XDR infection rate.

**Materials and methods**

The methods of study were used Development and Empowerment Program (DEP), which followed role and competency of Advanced Practice Nurse (APN). Data were gathered from medical and surgical departments from 1 January to 30 September 2015. There were 3 purposive selected groups: 1) Case Manager Team, 2) Medical and Surgical Nursing Teams and 3) XDR infected cases. The research tools were: 1) DEP, 2) Data questionnaires, 3) The XDR IC practice observational forms and 4) The evaluation quality of XDR cases forms. Data were analyzed using descriptive statistics and t-test.

**Results**

The results of study suggested that a model for nursing care should consisted of 3 components: 1) providing XDR infection control activities in process of care, 2) developed and empowered case managers in role and competency of APN and 3) feedback outcomes of caring and supporting system. After using DEP, the XDR infection rate significantly decreased from 4.1 to 1.6 per 100 patient-days (*p* < 0.05).

**Conclusions**

This study showed that using DEP in case managers and nurses promoted participants learning in teams. The DEP also aligned XDR caring processes with IC standard practices that resulted in decreased XDR infection rate in hospital efficiency.

## Session: Prevention of MDROs

### PM12 Development of clinical pathway for prevention and control of multidrug-resistant organisms transmission in hospital

#### Nattawipa boon kirdram, Wilawan Picheansathian, Pimpaporn Klunklin

##### Faculty of Nursing, Chiang Mai University, Chiang Mai, Thailand

###### **Correspondence:** Nattawipa boon kirdram (spun.11@hotmail.com)

**Background**

Transmission of multidrug-resistant organisms in hospitals has a direct impact on patients, health care personnel, the hospital, community, and the nation. This developmental research aimed to develop a clinical pathway for prevention and control of multidrug-resistant organism transmission in the medical department at a regional hospital.

**Materials and methods**

Study samples included 131 health care personnel who worked in the medical department and related including: hemodialysis, echocardiogram, radiology and ultrasound, CT scan, MRI, and stretcher. Ten patients infected or suspected of multidrug-resistant organism infection at the medical department were included in the test. The process for developing was based on Cheah’s (2000) framework. Three development steps were thus included: assessment and situation analysis, designing, and testing the implementation. The data collection instruments consisted of a demographic data record form and questionnaire assessing the opinions of health care personnel towards. The content validity of the questionnaire was examined by 5 experts and the content validity index was 0.90. Data were analyzed using descriptive statistics and data categorization.

**Results**

The results included: screening for multidrug-resistant organisms, using contact precautions, using masks when performing splash-generating procedures, cleaning patients’ bodies, cleaning the immediate environment, reminding health care personnel of best practices and proper transfer between units, active surveillance, and limiting medical errors and adverse events. Most health care personnel agreed that this clinical pathway was clear, convenient, feasible, practical and appropriate for implementation in their units.

**Conclusions**

This research suggests that should be routinely implemented in the hospital.

## Session: Prevention of Site Specific Infections

### P1 Best practice in device management – an Indian experience

#### Geetha Samethadka (gsvb@rediffmail.com)

##### Consultant microbiologist and infection control officer, Bhagwan Mahaveer Jain Hospital, Bangalore, Karnataka, India

**Background**

Device Associated Infections (DAI) increase morbidity, mortality and hospitalization costs. They account for most Hospital Acquired Infections (HAI). Regular and systematic surveillance of DAI reduces the risk of infections and complications. This study aimed to minimize risk of DAI in the 500 bed tertiary care hospital by careful device management through strict aseptic measures, documentation of prospective site-specific surveillance and assessing risk factors and implement healthcare practices for indwelling devices and policies to manage critical care bundles.

**Materials and methods**

Hospital-wide prospective, site-specific DAI surveillance was carried out during Jan 2015 – July 2016 and practices standardized as per National Accreditation Board for Hospitals (NABH) guidelines. Ventilator Associated Pneumonia (VAP), Central Line Associated Blood Stream Infection (CLABSI), Catheter Associated Urinary Tract Infection (CAUTI) and Surgical Site Infection (SSI) were among the DAI surveillance indicators tracked. Surveillance registers placed at various locations were checked daily during infection control rounds to take necessary corrective/control actions. Random patient checks were carried out to verify entries.

**Results**

Training, surveillance and care bundle checks led to significant reductions in DAI rates (per 1000 device days). VAP rate dropped from 16.80 to 8.28 over 4654 ventilator days (1454 intubations), CLABSI rate from 3.29 to 1.16 over15040 central line days (3275 central line insertions) and CAUTI rate from 1.8 to 1.3 across 39945 catheter days (10945 Foley’s catheter insertions). SSI rates reduced from 0.35% to 0.23% over 14955 surgical cases.

**Conclusions**

Indwelling devices and access systems are integral to in-patient care. Prospective site-specific surveillance, strict aseptic measures, compliance to critical care bundles and involvement of clinicians, nursing and support staff in device management helped in reducing incidence of DAI.

## Session: Prevention of Site Specific Infections

### P2 Reduction of central line-associated bloodstream infections by an education-based intervention with a simple tool in acute care hospital setting

#### Naoko Suzuki, Hitomi Asada, Masao Katayama, Atsushi Komano

##### National Hospital Organization Nagoya Medical Center, Nagoya, Aichi, Japan

###### **Correspondence:** Naoko Suzuki (nasuzuki18@gmail.com)

**Background**

Blood culture (BC) is critical to diagnose central line-associated bloodstream infections (CLABSI). Erroneous blood culture leads to inappropriate use of antibacterial medicine. To increase the accuracy of laboratory confirmation of BSI, technically sound specimen collection is critical. This study aimed to evaluate an education-based intervention with a simple tool to improve the quality of laboratory confirmation of CLBSI in an acute care hospital setting.

**Materials and methods**

CLABSI surveillance was performed for 24 months on intensive care unit (ICU), and hematology and surgical wards from 2013 to 2014. At 9th month, we distributed a quick visual guide for blood specimen collection procedure. At 10th and 20th months, an education and training opportunities were enforced to nurses. We assessed the effectiveness of an education-based intervention by comparing CLABSI during the first and the second 12-month periods.

**Results**

The number of patients on central venous catheter was 820 and 736 during the first and the second periods, respectively. Central line days were 10,032 and 9,666. Similar number of BC was examined and CLABSI was confirmed on 24 and 14 cases. Among them laboratory-confirmed bloodstream infection (LCBI) 2 occupied 12 and 3 cases. The number of CLBSI and the rate of LCBI2 were reduced (*P* = 0.096 and 0.017, by Pearson’s test).

**Conclusions**

We demonstrated that our education-based intervention program was effective to reduce the rate of LCBI 2 among CLBSI. This is primarily due to the lowering the risk of contamination of common commensal in the blood specimen.

## Session: Prevention of Site Specific Infections

### P3 Reduced incidence of peripheral vascular catheter-related bloodstream infections following a change in catheter duration to every 4 days

#### Akihiro Sato, Itaru Nakamura, Hidehiro Watanabe, Tetsuya Matsumoto

##### Tokyo Medical University Hospital, Shinjyuku-ku, Tokyo, Japan

###### **Correspondence:** Akihiro Sato (world_packer2006@yahoo.co.jp)

**Background**

Catheter-related bloodstream infection (CRBSI) is the most common cause of healthcare-associated bloodstream infection in inpatients. Data on peripheral vascular catheter-related bloodstream infections (PVC-BSIs) are insufficient compared with data on central venous catheter-related bloodstream infections (CVC-BSIs). The protocol of duration of insertion of PVCs in our hospital was changed from every 7 days to every 4 days in March 2016. We aimed to compare the incidence of PVC-BSIs before and after the change.

**Materials and methods**

We retrospectively studied data from positive blood cultures of 122 patients diagnosed with PVC-BSIs from June 1, 2010 to August 30, 2016 at Tokyo Medical University Hospital.

**Results**

The number of incidents of PVC-BSIs in the years 2010 to 2016 was 4, 5, 11, 16, 13, 49, and 24 cases, respectively. The monthly average from January 2015 to February 2016 was 4.1 cases, but from March 2016 to August 2016 the monthly average was 2.7 cases.

**Conclusions**

The Centers for Disease Control and Prevention state that PVCs do not need to be replaced more frequently than at 96-hour intervals, but recommend replacement at least every 7 days to reduce the risk of infection and phlebitis in adults. In our hospital, we modified this protocol to every 4 days because we observed many cases where the catheter site was not examined daily and the records of insert duration. This protocol change resulted in a reduction in the incidence of PVC-BSIs and may therefore represent a method to reduce this type of infection.

## Session: Prevention of Site Specific Infections

### P4 A two-year hospital-wide surveillance of central line-associated bloodstream infections in a Korean hospital

#### Hye Kyung Seo^1^, Joo-Hee Hwang^1^, Myoung Jin Shin^1^, Su Young Kim^1^, Eu Suk Kim^1,2^, Kyoung-Ho Song^2^, Hong Bin Kim^2^

##### ^1^Department of Internal Medicine, Seoul National University Bundang Hospital, Seongnam, Republic of Korea; ^2^Infection Control Office, Seoul National University Bundang Hospital, Seongnam, Republic of Korea

###### **Correspondence:** Hye Kyung Seo (shkid79@naver.com)

**Background**

Surveillance and interventions of central line-associated bloodstream infections (CLABSIs) had mainly been targeted in intensive care units (ICUs). Central lines as invasive procedures are increasingly used in hospitals outside of ICUs, especially in immunocompromised or elderly people. We performed a hospital-wide surveillance of CLABSIs to evaluate the current status and find out the future strategic plans to decrease CLBASI rates.

**Material and methods**

All patients having central lines were screened for CLABSIs at a 1,328-bed tertiary teaching hospital between January 2014 and December 2015 using the electronic surveillance program. Clinical information including types of central lines was collected. CLABSI rates were calculated using the definitions of National health and Safety Network (NHSN).

**Results**

A total of 154 CLABSIs was identified, of which 72 and 82 occurred in the general wards and the ICUs (0.81 and 2.71 per 1,000 catheter days), respectively. For 71 CLABSI events diagnosed within one week, 48 non-tunneled CVCs and 16 PICCs were in place, respectively. However, for 113 CLABSIs diagnosed after a week, non-tunneled CVCs, tunneled CVCs, and PICCs accounted for 44, 33, and 36 cases, respectively. While the majority (72.2%) of CLABSIs in the ICUs was related with non-tunneled CVCs, tunneled CVCs (38.9%) and PICCs (36.8%) were the most common types in general wards.

**Conclusions**

CLABSI rates in general wards were relatively low, but long-term indwelling catheters were more frequently used than in ICUs. Therefore, a tailored intervention should be implemented to prevent CLABSIs according to the wards.

## Session: Prevention of Site Specific Infections

### P5 Planning and implementation of an infection control training course for home-care nursing team

#### Lai-Si Un, Choi-Ian Vong

##### Health Bureau of Macau SARS, Macau, Macau

###### **Correspondence:** Lai-Si Un (mercy.un@gmail.com)

**Background**

The lack of well-trained, experienced infection control personnel prevents healthcare-associated infections in home-care nursing team. During our practicum of the postgraduate specialty-nursing program, we collaboratively planned and implemented a training course for prevention catheter-associated urinary tract infection (CAUTI) to address this need.

**Materials and methods**

The course relied on the guideline for prevention of CAUTI of Centers for Disease Control and Prevention of the United States of America. The CAUTI prevention program of Centers for Disease Control of Taiwan, the CAUTI prevention bundle of Centre for Health Protection of Hong Kong, collaboration with community nurses of health center and resources available at the Hong Kong Polytechnic University. The 1-hour course consisted of lecture session and case discussion, trained 9 nurses and 6 nurse students.

**Results**

The result of evaluation questionnaire showed that course content, teacher performance met the trainees’ needs, and that 100% trainees acquired the necessary knowledge. 60% of the trainees were “satisfied” and other 40% were “very satisfied”.

**Conclusions**

Our experience can serve as a model for other organizations interested in strengthening infection control and prevention at local sites.

## Session: Prevention of Site Specific Infections

### P6 Implementing an effective surgical site infection (SSI) surveillance: basis for quality improvement

#### Jose Paulo Flor, Nicolo Andrei Añonuevo, Marko Bautista, V James De Roxas, Justine Vergara, Marion Kwek

##### Asian Hospital and Medical Center, Muntinlupa, National Capital Region, Philippines

###### **Correspondence:** Nicolo Andrei Añonuevo (naanonuevo@asianhospital.com)

**Background**

Surgical site infection and surveillance remains to be a challenge in a developing country. This study aimed to decrease surgical site infections through a systematic process and a real-time monitoring.

**Materials and methods**

The methods used are daily rounds to check the wound of the post-operative patients while admitted, follow up phone calls to patients every 2 weeks until the patient get passed beyond the window period of possible surgical site infection occurrence and bundles of care for prevention of SSI activation. Through the use of SSI surveillance along with the bundles of care, surgical site infection cases were captured proactively by the Infection Prevention and Control Unit and interventions were implemented immediately targeting the source of infection. Feedback was provided to the involved department whose surgical site infection cases went beyond the benchmark.

**Results**

Through the process initiated, there was a decrease in surgical site infections from 1.9% in 2013 to 0.9% as of August 2016. This downward trend was managed despite of expansion in the procedures included in the surveillance.

**Conclusions**

It is therefore concluded that SSI Surveillance and bundles of care are effective way to promote safety among post- operative patients.

## Session: Prevention of Site Specific Infections

### P7 Prevention of catheter- associated urinary tract infection in a community hospital in Singapore

#### Jocelyn Koh^1^, Sherly Agustinus^2^, Rozita Bte Abu Hassan^2^, Yin Phyu Thinn^3^, Benjamin Ng^4^

##### ^1^Department of Medical Services, Ang Mo Kio-Thye Hua Kwan Hospital, Singapore, Singapore; ^2^Department of Nursing Services, Ang Mo Kio-Thye Hua Kwan Hospital, Singapore, Singapore; ^3^Department of Allied Health, Ang Mo Kio-Thye Hua Kwan Hospital, Singapore, Singapore; ^4^ Department of Health Performance Office, Ang Mo Kio-Thye Hua Kwan Hospital, Singapore, Singapore

###### **Correspondence:** Jocelyn Koh (jocelyn_koh@amkh.org.sg)

**Background**

Catheter-associated urinary tract infection (CAUTI) is a common hospital acquired infection which can lead to prolonged hospitalisation and mortality. Ang Mo Kio-Thye Hua Kwan Hospital (AMK-THKH) is a 360-bedded Community Hospital in Singapore, providing inpatient rehabilitation and subacute care. The majority of patients are geriatric and referred from tertiary hospitals. A multidisciplinary Quality Improvement team was formed in March 2015 to reduce CAUTI in AMK-THKH. The median CAUTI rate was 5.3 per 1000 catheter days in 2014. This study aimed to reduce CAUTI rate by 30% in 3 years.

**Material and methods**

Analysis of CAUTI cases showed that the majority had an indwelling catheter (IDC) on admission and developed CAUTI with the same IDC. The identified gaps were: Inefficient CAUTI data collection, lapses in IDC care and no IDC removal protocol.

Changes tested and implemented in stages at a pilot site:

- Improved CAUTI data collection methods
- IDC reminder system
- Nurse-led protocol to empower nurses to remove catheters in simple cases
- Using S hook to keep urine bag below bladder level during ambulation
- Daily maintenance audit of IDC care
- Frontline staffs were regularly engaged and feedback gathered. The changes were modified, tested and improved after (Plan-Do-Study-Act) PDSA cycles.

**Results**

Preliminary results in the pilot wing showed a reduction in CAUTI rate although it is still early to tell if results are sustainable. Results on compliance to IDC care have also been encouraging.

**Conclusions**

We plan to test and spread the changes to other wards to reduce CAUTI rate in the hospital. Getting feedback and buy-in from the ground helps in designing sustainable changes.

## Session: Prevention of Site Specific Infections

### P8 Clinical audit of urinary catheter documentation in admitted patients

#### Soe Pyae Tun^1^, Su Mon Thi Ha^1^, Xue Xiaoting^2^, Lin Li^1^, Leyland Chuang^1^

##### ^1^Department of Medicine, Ng Teng Fong General Hospital, Singapore, Singapore; ^2^Department of Nursing Training & Development, Ng Teng Fong General Hospital, Singapore, Singapore

###### **Correspondence:** Su Mon Thi Ha (sumon.thiha@mohh.com.sg)

**Background**

Urinary tract infection (UTI) remains the commonest nosocomial infection worldwide. Major guidelines recommend indications for urinary indwelling catheter (IDC) be documented at time of placement. This facilitates prompt evaluation for removal subsequently, thereby minimizing inappropriate catheterization and risks of UTI. We aimed to evaluate UTI prevention guideline compliance by reviewing indications for IDC placement and frequency of proper documentation.

**Material and methods**

General ward patients with an IDC in place between 5th to12th August 2016 were identified by chart review. Demographic and clinical data related to IDC placement were extracted.

**Results**

Among 46 patients with IDCs, documentation of urinary catheterization indication was performed by doctors in 25 patients (54.4%) and by nurses in 39 (84.8%). Four patients had no indications documented. Only 22 patients had indications documented by both nurses and doctors, of which 19 (86.4%) matched. Twenty-four patients (52.2%) did not have complete documentation. The indication most frequently documented by doctors was retention of urine (39.1%), followed by strict intake-output monitoring (37.0%) and perioperative monitoring (4.4%).

**Conclusions**

Documentation of indications for urinary catheterization is not adequate despite guideline recommendations. Documentation by doctors was less frequent compared to nurses, and indications may not match, thus indicating that it may have been inappropriate. Lack of documentation may cause difficulties in deciding if continued use of IDC is needed, and resulted in delaying catheter removal and increasing risks of catheter associated UTIs.

## Session: Prevention of Site Specific Infections

### P9 Intensive care nurses’ knowledge, attitudes and practices on ventilator associated pneumonia bundle approach

#### Attanayaka Mudiyanselage Chulani Niroshika, Kaluarachchige Anoma Kaluarachchi Perera, Dimingo Kankanamalage Diana Grace Fernando, Bodhipakshage Rohini Hemamala

##### National Hospital of Sri Lanka, Colombo, Sri Lanka

###### **Correspondence:** Attanayaka Mudiyanselage Chulani Niroshika (chulani.niroshika@ymail.com)

**Background**

Ventilator Associated Pneumonia (VAP) develops in patients who are mechanically ventilated more than 48 hours in intensive care units (ICU). Bundle approach can prevent VAP. We aimed to describe knowledge attitudes and practices regarding VAP bundle approach.

**Materials and methods**

A descriptive cross sectional study was conducted among nurses in Intensive Care Units (ICU) at the National Hospital of Sri Lanka. Data were collected using a pre-tested self-administered questionnaire.

**Results**

The median of the total scores for knowledge was 62.9%. Of 229 participated, 10% scored more than 75%, 60.7% scored 55% - 74%, 22.7% scored 41% - 54% while 6.6% scored below 40% marks. Participants knowledge was not associated with previous training on intensive care (p = 1.06), level of education (p = 1.24) and period of service in an ICU (p = 0.65). Their knowledge were high regarding the use of closed suction systems (80.4%), correct positioning of patients (85.6%) and the correct angle of bed elevation ( 82.1%) to prevent VAP. They have poor knowledge on frequency of changing humidifiers (14.85%) and changing of suction systems (7.86%). Lack of knowledge was evident in preventive strategies such as; selecting the site of the ET tube (42.4%), when to change ventilator circuits (48.9%) and use of kinetic or standard beds (56%) to reduce the risk of VAP. Overall self- reported practices were 75.4%. Poor practices were reported in wearing a polythene apron during ET Suctioning (35.8%).

**Conclusions**

Overall knowledge and attitudes towards VAP bundle among participants were satisfactory. Establishment of written protocols and regular monitoring would lead to improved practice outcomes.

## Session: Prevention of Site Specific Infections

### P10 To explore the factors and improving methods of low rate of respirator-associated pneumonia in intensive care unit

#### Chiu-yin Yeh, Huwi-chun Chao

##### Chimei medical center, Tainan, Taiwan

###### **Correspondence:** Chiu-yin Yeh (yehchiuyin@yahoo.com.tw)

**Background**

Ventilator-associated pneumonia (VAP) with VAP bundles, should result in dramatic reductions in the incidence of VAP. Compliance with the VAP bundle has been most successful when all elements are executed together as a \"all or none\" strategy. VAP increases patient mortality, hospital stay and medical expenses. According to the latest VAP criteria of the American Disease Control Agency, there are many difficulties and blind spots in the diagnosis of VAP, and the low rate of VAP in this hospital is expected to be improved by the study of VAP low reporting rate.

**Materials and methods**

The study of the hospital VAP low reporting rate of the underlying causes: First, the clinical symptoms and chest X-ray interpretation of the lack of uniform standards, lack of education and training and advocacy. Second, the existing receiving process can not immediately grasp the patient condition. Third, to strengthen the quality of X-ray film and rules to track. The team developed the criteria and reporting process for the diagnosis of high-risk VAP patients by the Centers for Disease Control, in order to facilitate VAP bundle in the care of patients with high-risk VAP infection.

**Results**

The rate of VAP in our hospital increased from 1‰ to 2‰, but it was significantly lower than that of foreign VAP.

**Conclusions**

Difficulty in diagnosing patients and blind spots in VAP are also a major limitation for clinicians. Of VAPs have a low reporting rate. Therefore, the proposed medical institutions to standardize the hospital medical behavior and infection control measures to cohesion team consensus.

## Session: Prevention of Site Specific Infections

### P11 Using interdisciplinary collaborative care to reduce central line – associated bloodstream infection density in the medical intensive care unit

#### Yang, Hui-Chun, Chiu, Hsiang-Ju, Shih, Ya-Ling

##### Department of Nursing, En Chu Kong Hospital, Sanxia, New Taipei City, Taiwan

###### **Correspondence:** Yang, Hui-Chun (12823@km.eck.org.tw)

**Background**

In 2013, rate of central line – associated bloodstream infection (CLABSI) in the medical intensive care unit of our hospital was 3.74%, which was higher than of surgical intensive care unit (2.87%) and Taiwan Clinical Performance Indicator from 33 regional hospitals (3.47%). Infection control unit, medical quality management center, department of medical, and purchasing department joined to develop strategies to reduce CLABSI in the Medical Intensive Care Unit (MICU).

**Materials and methods**

There were 4 steps: (1) inter-professional education; (2) create a central line insertion cart; (3) CHANGE (C: CVC, H: hand washing, A: antiseptic liquid, N: neck, G: giant, E: evaluation); (4) unscheduled audits to assess the members’ three abilities, including cognition, affection and technical skills.

**Results**

Regarding these strategies, we gained three effective outcomes as follows: (1) the CLABSI incidence density reduced from 3.74‰ in 2013 to 2.41‰ in 2014 (implementation period) and 1.22‰ in 2015; (2) compliance and accuracy rates of proper hand-washing techniques increased up to 100% and (3) bloodstream infection density reduced from 0.92‰ in 2013 to 0‰ in Jan – Aug 2014 period.

**Conclusions**

CLABSI incidence density was still low in MICU: 0‰ in Jan.-Aug. 2016 period. The team members found out that the qualities of care need interdisciplinary collaboration and cooperation. Furthermore, our members deeply realized importance of learning inter-professional education to do inter-professional practice, and that would be better for not only patients’ life but also professions’.

## Session: Prevention of Site Specific Infections

### P12 Reducing use of indwelling urinary catheters and associated urinary tract infections

#### Yu-Shan Chien, Wan-Yi Lin, Chia-Yun Pan, Ying-Yun Chang, Chiu-Yuch Yea, Ming-Hsien Chu, Li-Chu Lee

##### Taipei Veterans General Hospital, Yilan, Taiwan

###### **Correspondence:** Yu-Shan Chien (s7892720503@yahoo.com.tw)

**Background**

Urinary tract infections are the most common site of infection in medical health care institutions. The American Hospital Infection Surveillance System (NNIS) estimates that two million people have annually urinary tract infection. Catheter-associated urinary tract infection is the most common type of nosocomial infection (about 40%). The opportunity of occurrence of urinary tract infection will increase if indwelling catheter is placed for more than seven days, and also once a catheter is placed, the daily incidence of bacteriuria is about 3-8%. In 2015, indwelling catheter associated urinary tract infection rate was 3.35‰ that higher than other units. In order to effectively reduce the indwelling catheter associated urinary tract infection, we organized the team to improve this problem, in order to reduce the use of indwelling urinary catheters, catheter-associated urinary tract infection rates, to maintain patient safety, and to improve quality of the care of indwelling catheter.

**Materials and methods**

(1) The concept of nursing staff was not correct, education and training were incomplete. (2) Physicians easily forgot patients with catheterization. (3) Paper glue caused skin damage easily, urine bag fixed rope too loose to touch the ground, and nursing assistants did not know the perineum cleaning methods. (4) The concept of nursing assistants was incorrect.

**Results**

The indwelling catheter associated urinary tract infection rate dropped to 0‰.

**Conclusions**

Through transdisciplinary, it could improve the quality of medical service and care, but also to enhance the care of the implementation of infection control.

## Session: Prevention of Site Specific Infections

### P13 Using of “bundle care” to reduce ventilator-associated pneumonia density in the surgical intensive care unit

#### Hsiang-Ju Chiu, Ya-Ling Shih, Hui-Chun Yang

##### Department of Nursing, En Chu Kong Hospital, New Taipei City, Taiwan

###### **Correspondence:** Hsiang-Ju Chiu (famy1981@gmail.com)

**Background**

Ventilator-associated pneumonia (VAP) is one of the most common nosocomial infection and an important issue in intensive care units (ICU). VAP density in SICU was as high as 12.1‰ in the fourth quarter of 2014. Two main reasons were found after analysis: (1) The compliance of hand hygiene was only 59.3%; (2) There was no guideline to follow when nurses executed oral care for endotracheal intubation patients.

**Materials and methods**

The following intervention measures to reduce VAP density: (1) Announce five timing of hand hygiene in unit regularly; (2) Check compliance and correct rate of hand hygiene; (3) Combine bundle care to set up the operating standards of oral care for endotracheal intubation patients; (4) Conduct education about VAP Bundle care; (5) When nurses perform oral care, use 0.12-0.2% CHG-containing; (6) Design creative reminder marked on towel and poster on the wall; (7) Paste red label at the place of 30 degree of head up; (8) Monitor regularly.

**Results**

Average VAP density was 0% from January to July in 2016.

**Conclusions**

We conclude that the implementation of VAP Bundle care, increase in software and hardware equipment, education, follow up, and monitor regularly can reduce VAP density effectively and the quality of care in ICU drastically. It can take forward to parallel implementing a hospital-wide program to enhancing patient safety effectively in 2016.

## Session: Prevention of Site Specific Infections

### P14 Analysis of ventilator-associated pneumonia and the effectiveness of VAP bundle intervention in ICU of one southern regional hospital

#### Lin Yu-Hsiu, Guo Siao-Pei, Leung Pak-On, Sie Mei-Fe, Chen Jyh-Jou

##### Chi Mei Medical Center, Liouying, Tainan, Taiwan

###### **Correspondence:** Lin Yu-Hsiu (t940808@mail.chimei.org.tw)

**Background**

Bundles strategies improve health care-associated rate in intensive care units. We use ventilator-associated pneumonia (VAP) bundle intervention to analyze the VAP rates and its effectiveness.

**Materials and methods**

The study design was a single group interrupted time series study. The VAP bundle (involves bedside elevation of 30-45 degrees, daily oral care with appropriate disinfectants, daily sedation discontinuation, venting of respirator tubing, daily assessment. Whether the removal of catheters) was implemented on all patients admitted to the ICU starting 2015. Data of VAP rates from 2015 and 2016 were pooled to compare the differences between observed values and predicted values before and after the intervention.

**Results**

Statistics in July 2015 ~ June 2016 compliance rate was 81.5%. (P = 0.789), the VAP rates of Department of Internal Medicine increased from 1.64 to 2.12 (p = 0.228), and that of Surgery decreased from 2.39 to 1.68 (p = 0.228), while that of Department of Respiratory Medicine decreased from 1.77 to 1.68 (p = 0.159), and the cardiology care unit was reduced from 0.72 to 0.48 (p = 0.49). Among them, 3BICU Internal Medicine Intensive Care Unit (ICU) had the best effect, which decreased from 2.5 to 0.0 (p = 0.001) before intervention, up to 13 months.

**Conclusions**

Our study found that the effective use of all component of the VAP bundles successfully reduce Ventilator-associated pneumonia infection rates.

## Session: Prevention of Site Specific Infections

### P15 Time series analysis of catheter-associated bloodstream infection and the effectiveness of CVC bundle intervention in ICU of one southern regional hospital

#### LinYu-Hsiu^1^, ChangYong-Yuan^2^

##### ^1^Chi Mei Medical Center, Liouying, Tainan, Taiwan; ^2^Kaohsiung Medical University, Kaohsiung, Taiwan

###### **Correspondence:** LinYu-Hsiu (t940808@mail.chimei.org.tw)

**Background**

Bundles strategies improve health care-associated rate in intensive care units. The aim of study was to assess an interrupted time series study of catheter-related bloodstream infection (CRBSI) rate after the intervention of the central vascular catheter bundle care. Effectiveness of the central vascular catheter bundle care, and the risk factors of central vascular catheter-related bloodstream.

**Materials and methods**

The CVC bundle was implemented on all patients admitted to the ICU starting April 2013. Data of CRBSI rates from 2012 and 2015 were pooled to compare the differences between observed values and predicted values before and after the intervention. CVC bundle care was implemented since April 2016.

**Results**

Our study showed that the central catheter bloodstream infection rate was 1.4‰ (P = .039), the ventilator-associated pneumonia (VAP) infection rate was 2.0‰ (P = .72), the catheter associated urinary tract (CA-UTI) infection rate was 2.35‰ (P = .78), and the ICU infection rate was 5.10‰ (P = .025). The overall central catheter utilization rate was 58.88% (P = .086); the mean length of ICU (intensive care unit) stay was 15.72 days, and the average length of a hospital stay was 43.57 days.

**Conclusions**

The time sequence analysis model was adopted to analyze the catheter-related bloodstream infection rate in 2013. Although it did not decrease, but only slowed down the trend of increase the CRBSI incidence. To effectively reduce the catheter-related bloodstream infection rate is to persistently support the combination of CVC bundle care and to enhance the compliance of the policy.

## Session: Prevention of Site Specific Infections

### P16 The clinical benefit of chlorhexidine gel in severe units was explored

#### Shu-Yuan Kuo, Yu-Hsiu Lin, Ji-Sheng Zhang, Pak-On Leung, Mei-Fe Sie, Jyh-Jou Chen

##### Chi Mei Medical Center, Liouying, Tainan, Taiwan

###### **Correspondence:** Shu-Yuan Kuo (a30163@mail.chimei.org.tw)

**Background**

Several studies have shown that proper salivary secretion may help or reduce the risk of VAP for maintaining oral hygiene and for clinical care through chlorhexidine mouthwash or gel. The Department of Nursing, Nursing Department, Infection Control Group jointly launched the case, the full use of chlorhexidine gel instead of the original chlorhexidine mouthwash, and with the adjustment of clinical work standards, trying to analyze whether the introduction of new care model can effectively reduce the VAP. And clinical care hours to achieve a win-win results.

**Materials and methods**

Through cross-team collaboration, it is planned to provide Chlorhexidine gel for oral care in June-July 2016, with a total service population of 628. Oral care takes about 15 minutes / person through the new chlorhexidine, and about 20 minutes with chlorhexidine mouthwash. Chlorhexidine gel (market price 50 grams / 650 yuan) and the care of people were relatively chlorhexidine mouthwash costs (market price of 200 grams (bottle) / 130 yuan) is relatively low.

**Results**

The use of chlorexidine gel containing chlorhexidine mouthwash compared with the use of oral disinfection can be reduced by about 42,250 yuan. Nursing care hours, about 78.5 hours per month can be reduced. The density of VAP was maintained at 0.64‰.

**Conclusions**

The use of chlorhexidine gel for mouth disinfection 54% less than the cost of mouthwash and save 33% of the time, not only reduce the cost of medical expenses also save the number of clinical nursing work, effectively improve the medical quality.

## Session: Prevention of Site Specific Infections

### P17 To investigate the feasibility and compliance of the male catheterization in clinical practice

#### Yan-Ru Chen, Yu-Hsiu Lin

##### Chi Mei Medical Center,Liouying, Tainan, Taiwan

###### **Correspondence:** Yan-Ru Chen (c89040507@yahoo.com.tw)

**Background**

Urinary tract infections (UTIs) are one of the most common sites of health care-associated infection, accounting for about 40%, of which about 70% are related to the use of catheters. In our subacute respiratory care unit, 62% of the CAUTI cases were men, whereas those who performed the technique clinically were nurse practitioner, through clinical monitoring and found that nurse practitioner to implement this technology compliance rate of only 25%. The aim of this study was to assess the feasibility and compliance of catheterization among male patients with competition and questionnaires.

**Materials and methods**

The standard operating procedures of "Male Catheterization" were developed and practiced in clinical practice by means of practical operation and questionnaires.

**Results**

A total of 93 participants were involved in this competition, with a rate of 96.88%. Among them, 9 of them (9.68%) were not up to 60 points, and 3 of them were below 80% according to the technical project contents, respectively:

1. Pre-hand hygiene.
2. The first disinfection and wait disinfectant dry.
3. The second disinfection.

After the competition, the questionnaire is investigated and analyzed as follows:

1. Professional nurse practitioner years: more than 5 years (36%) accounted for the highest.
2. 72% felt it was suitable for clinical practice.
3. Difficult to implement technology: the proportion of the first disinfection accounted for the highest (52%), followed by wearing sterile gloves to no Bacteria were connected to the catheter and urine bag (20%), and again the hand hygiene (16%).

**Conclusions**

This technology is standardized and it is recommended to incorporate the new training content of the Nurse Practitioner.

## Session: Prevention of Site Specific Infections

### P18 The impact of bundle care intervention reduce catheter-associated urinary tract infections (CAUTIs): experience in a medical center in Taiwan

#### Ying-Ling Chen, Chi-Fen Taou, Hsiao-Shan Chen, Hung-Jen Tang

##### Infection Control Committee, Chi Mei Medical Center, Tainan, Taiwan

###### **Correspondence:** Ying-Ling Chen (ingling1125@gmail.com)

**Background**

The impact of invasive medical measures on patients has both advantages and disadvantages. There are 15% to 25% of inpatients will use Foley catheter. Patients with Foley catheter may cause bacteriuria, asymptomatic bacteriuria, or even symptomatic urinary tract infection. According to the Taiwan Nosocomial Infections Surveillance System (TNIS), from 2005 to 2014, the most common type of health care association infection in the intensive care unit of the medical center was catheter-associated urinary tract infection, accounting for 33% to 44%. Using bundle care to reduce catheter-associated urinary tract infection (CAUTI).

**Materials and methods**

From 2015 June to 2016 June, we follow the Taiwan CDC's " Invasive Care Quality Improvement Program", use bundle care measures in internal care intensive care unit respiratory intensive care unit medical ward surgical ward and neurology ward. The five elements of CAUTI bundle measures are (1) Hand hygiene, (2) Insertion of catheter using aseptic technique, (3) Unobstructed and close drainage system, (4)Tendency to lower of fixed urine bag than bladder, (5) Assessing the needs of catheterization daily.

**Results**

During this period, the CAUTI of internal care intensive care unit was decreased from 2.71 to 2.43 per 1000 catheter-days, respiratory intensive care unit was decreased from 5.71 to 3.89 per 1000 catheter-days, medical ward was decreased from 3.78 to 1.95 per 1000 catheter-days, surgical ward was increased from 1.35 to 1.86 per 1000 catheter-days, and neurology ward was decreased from 4.37 to 3.26 per 1000 catheter-days.

**Conclusions**

This program validates that bundle care measures is helpful in reducing CAUTIs. Further to avoid the secondary bloodstream infection, reduce hospital stay and reduce the consumption of medical resources.

## Session: Prevention of Site Specific Infections

### P19 The effect of replacing small-volume nebulizers on contamination after patient surgery

#### Shin Yu Chen, Yin Yin Chen, Fu Der Wang

##### Infection control, Taipei Veterans General Hospital, Taipei, Taiwan

###### **Correspondence:** Shin Yu Chen (sychen17@vghtpe.gov.tw)

**Background**

Nebulizers are widely used in clinical for treating the disease of respiratory tract, which can aerosolize medicine to 0.5-10 μm to reaching the lower respiratory tract, so as to induce expectorant action or treat the inflammatory of respiratory tract. However, nebulizers can be contaminated by the exogenous route owing to improper care, so that increase risks of oral colonization or respiratory tract infection. The previous study showed that suggestion about the frequency of replacing nebulizers and nebulizers practices were vary variable and inconsistent. The aim of this study was to evaluate the effect of replacing nebulizers (at 24, 48 or 72 hours) in contamination.

**Material and methods**

A randomized controlled trial study is conducted from July 9, 2014 to February 2, 2015 in a surgical ward of a medical center in Taipei. During the study period, include above 20 years old people with steam in halation treatment. After participants agree to join the trail, randomise cases into 24, 48 or 72-hour group. In the protocol, researcher collect culture of nebulizers after used.

**Results**

Total 525 specimens of nebulizes are collected. The rate of contaminated nebulizers at 24 hours is 14.1%, which is significantly higher (p = 0.008) in 48 hours (24.3%) and 72-hour group (25.6%). Multivariate statistical analysis show that steam inhalation increase by one day with the contaminated risk of nebulizers increased by 12% (95% Confidence interval: 1.03-1.21, p = 0.003).

**Conclusions**

The replacing nebulizers more than two days will increase contaminated rates significantly. Therefore, health care workers should understand and comply with guidelines of steam inhalation clinical care to reduce the contaminated risk of nebulizer.

## Session: Prevention of Site Specific Infections

### P20 Educational programs increase the compliance of bundle care: experience in a regional hospital in Taiwan

#### Tzu-Ping Shih^1,3^, Chin-Yu Chen^1^, Su-Jung Chen^1,2^, Mei-chi Wu^1^, Wan-ju Yang^1^, Mei-ling Chou^1^, Man-Ling Yu^1^, Li-Chu Li^1^, Cheng-Wei Chu^1^, Wen-Hao Tsou^1^, Wen-Chih Wu^1^, Wen-Chi Cheng^1^, Cho-Ching Sun^1^

##### ^1^Taipei Veterans General Hospital Yuanshan Branch, Taipei, Taiwan; ^2^Institute of Public Health and School of Medicine, National Yang-Ming University, Taipei, Taiwan; ^3^Mary's Junior College Of Medicing And Management, Yilan, Taiwan

###### **Correspondence:** Tzu-Ping Shih (520inf@gmail.com)

**Background**

Taipei Veterans General Hospital Yuanshan Branch is a 471 bed regional hospital that provides single veterans acute and chronic care. There is a team of care providers helping the nursing staff manage patient’s daily care. Urinary tract infections (UTIs) are common hospital-acquired infections accounting for 15% of HAIs. Recent studies and guidelines suggested that multidisciplinary care bundle could effectively reduce the incidence of catheter-related urinary tract infections (CAUTIs). The aim of this study was to evaluate the impact of educational programs for care providers on compliance of CAUTIs bundles.

**Materials and methods**

The intervention study was conducted over a period of 3 years, between 2012 and 2015. During a pre-intervention phase, parameters of compliance including the correct rate of had hygiene and correct rate of aseptic techniques were collected and compared with those during the post-intervention phase. The educational programs included (1) posters reminders on the walls of bedside, (2) aseptic urine sampling and cleaning technique, (3) correct draining bag positioning, (4) frequent tests and feedbacks to supervisors, (5) hand hygiene.

**Results**

During the study period,a total of 21 care providers received the training programs. The pre-intervention phase was between 2012 and 2014, the post-intervention phase was 2015. The correct rate of hand hygiene increased from 72.7% to 95.2%; the correct rate of aseptic techniques increased from 54.5% to 92.8%.

**Conclusions**

Our investigation showed that implementing educational programs for care providers increased the compliance of bundle care of CAUTIs. However, whether the improvement reduced the incidence of CAUTIs needed further investigations.

## Session: Prevention of Site Specific Infections

### P21 Impact of implementing VAP bundle: experience in a veteran’s regional hospital in Taiwan

#### Tzu-Ping Shih^1,9^, Chin-Yu Chen^1^, Shu-Hua Lu^4^, Su-Jung Chen^1,7,8^, Hsin-Ling Yang^2^ ,Cheng-Yu Lu^2^, Man-Ling Yu^2^, Li-Chu Li^2^, Cheng-Wei Chu^3^, Wen-Hao Tsou^4^, Wen-Chih Wu^5^, Wen-Chi Cheng^5^, Cho-Ching Sun^6^

##### ^1^Infection Control Committee, Taipei Veterans General Hospital Yuanshan Branch, Taipei, Taiwan; ^2^Department of Nursing, Taipei Veterans General Hospital Yuanshan Branch, Taipei, Taiwan; ^3^Department of Medicine, Taipei Veterans General Hospital Yuanshan Branch, Taipei, Taiwan; ^4^Department of Surgery, Taipei Veterans General Hospital Yuanshan Branch, Taipei, Taiwan; ^5^Vice Superintendent, Taipei Veterans General Hospital Yuanshan Branch, Taipei, Taiwan; ^6^Superintendent, Taipei Veterans General Hospital Yuanshan Branch, Taipei, Taiwan; ^7^Institute of Public Health and School of Medicine, National Yang-Ming University, Taipei, Taiwan; ^8^Section of Infectious Diseases, Department of Medicine, Taipei Veterans General Hospital, Taipei, Taiwan; ^9^Department of Teaching units, ST. Mary's Junior College Of Medicing And Management, Yilan, Taiwan

###### **Correspondence:** Tzu-Ping Shih (520inf@gmail.com)

**Background**

Experience in a veteran’s regional hospital is a 471 bed (in intensive care unit is a 10 bed), many critically ill patients need to rely on ventilator for survived. Although preventable, ventilator-associated pneumonia (VAP) is a main cause of death due to healthcare-related infection in the intensive care unit (ICU). Recent studies and guidelines suggested that multidisciplinary care bundle could effectively reduce the incidence of catheter-related infections. The aim of this study was to assess implementation of VAP bundle, can be effective in preventing ventilator-associated pneumonia occurred.

**Materials and methods**

VAP bundle care was implemented in a medical ICU of our hospital from March 2015 to March 2016. The contents of VAP bundle checklist including daily (1) readiness-to-wean assessment daily, (2) diary “sedation vacation”, (3) hand hygiene, (4) oral care every 12 hours, (5) head-of-bed elevation above 30 degrees. Education, training, and regular feedback from external auditing units can encourage the clinical care team to improve implementation rates.

**Results**

The results showed a daily goal checklist implementation rate increase from 38% to 100%. The ventilator bundle compliance rate, assessed by external audit, increased from 62% to 98%. The ventilator usage rate decreased 8.1% after implanted VAP bundle. The implementation of the VAP bundle during 1 year. A record of 12-month period of zero VAP case from March 2015 to March 2016 had achieved.

**Conclusions**

Our experience has shown that the implementation of VAP bundle, can be effective in prevention ventilator-associated pneumonia occurred. In addition, staff education and regular feedback from external audit data can improve implementation rates.

## Session: Prevention of Site Specific Infections

### P22 Efficacy of ventilator-associated pneumonia care bundle

#### Nitchawan Hirunprapakorn, Kumthorn Malathum, Sirilux Apivanich, Ttipakorn Pornmee

##### Ramathibodi Hospital, Bangkok, Thailand

###### **Correspondence:** Nitchawan Hirunprapakorn (giftnitcha@gmail.com)

**Background**

Ventilator-associated pneumonia (VAP) is a major healthcare-associated infection worldwide. The incidence of VAP in our hospital was around 3 episodes/1000 ventilator-days for many years. This study aimed to reduce VAP rates among patients in ICUs.

**Materials and methods**

This prospective study was done among mechanically ventilated adult patients in every ICU between January 1st, 2014 and December 31st, 2015. A total of 40,601 ventilation days were observed. A 5-element care bundle including daily assessment of readiness for ventilator weaning, hand hygiene, aspiration precautions, prevention of contamination and oral care using 0.12% chlorhexidine mouthwash were introduced to all ICU. Infection control nurses regularly visited and supervised the practice and periodic meeting among ICU nurses were arranged. The adherence was monitored.

**Results**

The incidence of VAP was gradually decreased, from 3.1 in 2013, to 2.8 and 2.5 per 1,000 ventilator-days in 2014 and 2015, respectively. The adherence rate to hand hygiene before tracheal suctioning was 44.7% at the beginning and 66.3% during the last period of the study while adherence to other measures were 93.4% throughout.

**Conclusions**

This study suggests that participation and experience sharing among nurses, regular monitoring, and supervision significantly contributed to the reduction of VAP.

## Session: Prevention of Site Specific Infections

### P23 Poor predictive performance of the newnational healthcare safety network (NHSN) surgical site infection risk adjustment model for craniotomy

#### Chonnikarnt Beowsomboon, Songyos Rajborirug, Yada Pruekrattananapa, Tharntip Sangsuwan, Silom Jamulitrat

##### Department of Community Medicine, Faculty of Medicine, Prince of Songkla University, Songkla, Thailand

###### **Correspondence:** Chonnikarnt Beowsomboon (ic_conference@yahoo.com)

**Background**

CDC NHSN recently introduced the new risk adjustment model for standardized surgical site infection (SSI) rate. However this model has not been tested in other institutes. Our study aimed to evaluate the predictive performance of CDC NHSN surgical site infection risk adjustment model for craniotomy.

**Material and methods**

Surveillance of data for post-craniotomy infection were retrieved from Infection Control Unit of Songklanagarind Hospital. Which surveillance system use the former National Nosocomial Infections Surveillance System (NNIS) risk index for SSI risk adjustment. The data included 2437 patients underwent 3250 brain surgeries in the hospital from September 2004 to June 2016. Medical records were reviewed for additional information including emergency operation, traumatic brain injury, brain cancer, and duration of steroid administration. The predictive performance of the former NNIS risk index, new NHSN model, and our proposed model was then compared by mean of area under receiver operating curve (AUC).

**Results**

The study identified 157 operations with post craniotomy SSI while the former and new model predicted 64.3 and 174.9 SSIs respectively. AUC of the new model (56.3%; 95%C.I = 52.1-60.4) was significantly (P-value = 0.02) less than the former one (50.7%; 95%C.I = 46.2-55.2). We analyzed the data and construct a model which included brain cancer, duration of operation more than 3 hours, and contaminated or dirty/infected wound class. AUC of our model was 59.8% (95%C.I = 55.7-63.8).

**Conclusions**

The new CDC NHSN model is poor for risk stratification postcraniotomy SSI and may be improved by adding brain cancer variable into the model.

## Session: Prevention of Site Specific Infections

### P24 Effects of coaching on nurses’ knowledge and practices regarding urinary tract infection prevention in older persons in long-term care facilities

#### Itthaporn Kumkoom^1^, Nongyao Kasatpibal^2^, Jittaporn Chitreecheur^2^

##### ^1^Registered Nurse, Saint Louis Hospital, Bangkok, Thailand; ^2^Associate Professor, Faculty of Nursing, Chiang Mai University, Chiang Mai, Thailand

###### **Correspondence:** Itthaporn Kumkoom (dittha58@gmail.com)

**Background**

Catheter-associated urinary tract infections (CAUTIs) are a common infection among elderly in long-term care facilities (LTCFs). Nursing personnel play an important role in prevention of CAUTIs. This study aimed to examine the effects of coaching on nurses’ knowledge and practice regarding CAUTIs prevention in elderly in LTCFs.

**Materials and methods**

A quasi-experimental study was performed during October 2014 to January 2015. The participants were 16 nursing personnel working in LTCFs. All samples attended the four steps of coaching intervention including 1) preparation; 2) discussion; 3) active coaching; and 4) follow-up. The research tools consisted of a demographic data questionnaire, a knowledge test, an observation form, a coaching plan, and a manual for prevention of CAUTIs. Data were analyzed using descriptive statistics, paired t-test, and Chi-square test.

**Results**

All of participants were female. The mean age was 31.9 years old, and the mean working experience was 8.7 years. Most participants had no experience in training of CAUTI prevention in elderly patients (87.5%). After coaching, average knowledge scores on prevention of CAUTI among nursing personnel increased significantly from 13.3 points to 19.6 points (p < .001) and the proportion of correct practices on prevention of CAUTIs in nursing personnel was statistically higher than the pre-coaching rising from 74.3% to 98.0% (p-value < 0.001).

**Conclusions**

The findings of this study suggest that coaching could enhance knowledge and practice skills in prevention of CAUTIs among nursing personnel in the LTCFs. Application of coaching may improve knowledge and practices in prevention of CAUTIs among nursing personnel in other LTCFs.

## Session: Prevention of Site Specific Infections

### P25 Effectiveness of probiotic, prebiotic and synbiotic therapies in reducing postoperative complications: a systematic review and network meta-analysis

#### Nongyao Kasatpibal^1^, JoAnne D. Whitney^2^, Surasak Saokaew^3,4^, Kirati Kengkla^3^, Margaret M. Heitkemper^2^, Anucha Apisarnthanarak^5^

##### ^1^Division of Nursing Science, Faculty of Nursing, Chiang Mai University, Chiang Mai, Thailand; ^2^Department of Biobehavioral Nursing and Health Systems, School of Nursing, University of Washington, Seattle, WA, USA; ^3^Center of Health Outcomes Research and Therapeutic Safety (Cohorts), School of Pharmaceutical Sciences, University of Phayao, Phayao, Thailand; ^4^School of Pharmacy, Monash University Malaysia, Selangor Darul Ehsan, Malaysia; ^5^Division of Infectious Diseases, Thammasart University Hospital, Pratumthani, Thailand

###### **Correspondence:** Nongyao Kasatpibal (nongyaok2003@gmail.com)

**Background**

Microbiome-directed therapies are increasingly used preoperatively and postoperatively to improve postoperative outcomes. Recently, the effectiveness of probiotics, prebiotics, and synbiotics in reducing postoperative complications (POCs) has been questioned. This systematic review aimed to examine and rank the effectiveness of these therapies on POCs in adult surgical patients.

**Methods and methods**

We searched for articles from PubMed, Embase, Cochrane, Web of Science, Scopus, and CINAHL plus. From 2002 to 2015, thirty-one articles meeting the inclusion criteria were identified in the literature. Risk of bias and heterogeneity were assessed. Network meta-analyses (NMA) were performed using random-effects modelling to obtain estimates for study outcomes. Risk ratios (RRs) and 95% confidence intervals (CIs) were estimated. We then ranked the comparative effects of all regimens with the surface under the cumulative ranking (SUCRA) probabilities.

**Results**

A total of 2,952 patients were included. We found that synbiotic therapy was the best regimen in reducing surgical site infection (SSI) (RR = 0.28; 95%CI, 0.12-0.64) in adult surgical patients. Synbiotic therapy was also the best intervention to reduce pneumonia (RR = 0.28; 95%CI, 0.09-0.90), sepsis (RR = 0.09; 95%CI, 0.01-0.94), hospital stay (mean = 9.66 days, 95%CI, 7.60-11.72), and duration of antibiotic administration (mean = 5.61 days, 95%CI, 3.19-8.02). No regimen significantly reduced mortality.

**Conclusions**

This systematic review suggests that synbiotic therapy is the best regimen to reduce SSI, pneumonia, sepsis, hospital stay, and antibiotic use. Surgeons should consider the use of synbiotics as an adjunctive therapy to prevent POCs among adult surgical patients. Increasing use of synbiotics may help to reduce the use of antibiotics and multidrug resistance.

## Session: Prevention of Site Specific Infections

### P26 Factors associated with catheter-associated urinary tract infections in the surgical intensive care unit and medical step-down unit at Ramathibodi Hospital

#### Thanomvong Muntajit, Siriluk Apivanich, Kumthorn Malathum, Somporn Somsakul

##### Ramathibodi Hospital, Bangkok, Thailand

###### **Correspondence:** Thanomvong Muntajit (thanomvong.mun@mahidol.ac.th)

**Background**

The incidence of catheter-associated urinary tract infection (CAUTI) is a major healthcare-associated infection in Ramathibodi Hospital. The incidence was more than 10 episodes/1000 urinary catheter-day for many years. We studied the effect of the intervention programs to reduce the incidence of catheter-associated urinary tract infection.

**Materials and methods**

A quasi-experimental research was conducted in the surgical intensive care and medical step-down units. The baseline and intervention period was from January to June 2012; and from September 2012 to May 2013, respectively. The CAUTI bundle according to the CDC’s guidelines recommendation was employed.

**Results**

We found non-significant reduction in the rates of CAUTI (15.83 versus 13.12 episodes/1000 urinary catheter-day) and urinary catheter utilization ratio (0.76 versus 0.67). Removal of catheter was not successful in patients with urinary retention, which were major population in our study. Multiple logistic regression revealed that duration of hospitalization >10 days (OR 2.84, 95% CI = 1.35-5.97), duration of indwelling catheter >7 days (OR 6.03, 95% CI = 2.85- 12.75), and female gender (OR 1.97, 95% CI = 1.12-3.49) were associated with a higher incidence of CAUTI in the medical step-down unit, while >8 days of hospitalization was associated with CAUTI (OR 7.42,95% CI = 1.39- 39.64) in the surgical intensive care unit. The most common organisms identified in both units were fungus and *E. coli*.

**Conclusions**

Prolonged catheterization is associated with higher incidence of CAUTI and early removal of catheter may not be achievable in certain group of patients. Further study is needed to determine the optimum care of patients who need indwelling catheters.

## Session: Prevention of Site Specific Infections

### P27 Surgical site infection after obstetric and gynecology surgery in Hung Vuong Hospital, Vietnam

#### Hang Thi Phan^1^, Anh Pham Phuong Dinh^2^, Tuyet Thi Kim Nguyen^2^

##### ^1^Quality management division in Hung Vuong hospital, Ho Chi Minh city, Viet Nam; ^2^Infection control department in Hung Vuong Hospital, Ho Chi Minh city, Viet Nam

###### **Correspondence:** Anh Pham Phuong Dinh (thuyhangytcc@gmail.com)

**Background**

Surgical site infection (SSI) is the most common hospital acquired infection at obstetrics and gynecology hospital. SSI prolonged hospital stay and increased treatment costs. We aimed to identify incidence and causative organism of surgical site infection among cesarean section and gynecology surgery cases.

**Materials and methods**

A prospective cohort study was conducted at Hung Vuong Hospital, Viet Nam from January 2015 to May 2016. Women who underwent caesarean and gynecological surgery were included in the study. CDC criteria (2009) were used to diagnose SSI.

**Results**

29,681 women underwent caesarean and gynecological surgery. SSI rate was 1.1% (95% CI: 0.98 – 1.22%), in which 0.96% SSI after caesarean section (95% CI: 0.84 – 1.08%) and 1.7% SSI after gynecological surgery (6.26% SSI after hysterectomy with 95% CI: 4.91 – 7.79%). Average number of treatment days that with SSI is 9 ± 4.5 days. *Staphylococcus aureus* and *coagulase-negative staphylococci* were the most common organism caused SSI after caesarean section (50%) and *Escherichia coli* was the main organism for SSI after gynecology operation (66.7%).

**Conclusions**

The incidence of SSIs was limited because we only follow patients with SSIs who were treated in hospital. The incidence of SSIs after hysterectomy surgery was higher than other types of gynecologic surgery. It’s necessary to promote infection control to reduce SSI in hysterectomy surgery.

