# Erratum to: Abstracts from the 8th International Congress of the Asia Pacific Society of Infection Control (APSIC)

**DOI:** 10.1186/s13756-017-0194-z

**Published:** 2017-05-23

**Authors:** Teresa Conceição, Hermínia de Lencastre, Marta Aires-de-Sousa, Rocio Alvarez Marin, Marta Aires de Sousa, Nicolas Kieffer, Patrice Nordmann, Laurent Poirel, Wison Laochareonsuk, Sireekul Petyu, Pawin Wanasitchaiwat, Sutasinee Thana, Chollathip Bunyaphongphan, Woranan Boonsomsuk, Pakpoom Maneepongpermpoon, Silom Jamulitrat, Terrence Rohan Chinniah, Kavitha Prabu, Rashidah Ahmad, Susylawathi Magon, Jauharatud DiniSuhaimi, Aizzuddin Mirasin, Nurul Morni, Boon Chu, Azizah Samsuddin, Aliyah Ahmad, Amalina Sidek, Noraini Ajis, Amalina AbuBakar, Amanie Shafiee, Julaini Safar, Ming-Chin Chan, Chih-Chien Wang, Nattawipa Boonkirdram, Wilawan Picheansathian, Pimpaporn Klunklin, Hang Thi Phan, Anh Pham Phuong Dinh, Tuyet Thi Kim Nguyen

**Affiliations:** 1 Laboratory of Molecular Genetics, Instituto de Tecnologia Química e Biológica António Xavier, Universidade Nova de Lisboa, Oeiras, Portugal; 2 Laboratory of Microbiology and Infectious Diseases, The Rockefeller University, New York, NY USA; 3 Escola Superior de Saúde da Cruz Vermelha Portuguesa, Lisboa, Portugal; 4 Medical and Molecular Microbiology Unit, Department of Medicine, University of Fribourg, Fribourg, Switzerland; 5 Hospital Universitario Virgen del Rocio y Virgen Macarena, Seville, Spain; 6 Escola Superior de Saude da Cruz Vermelha Portuguesa, Lisbon, Portugal; 7 University of Lausanne and University Hospital Center, Lausanne, Switzerland; 8 Prince of Songkla University, Hat Yai, Songkla Thailand; 9 Raja Isteri Pengiran Anak Saleha Hospital, Bandar Seri Begawan, Brunei Muara Brunei Darussalam; 10 Infection Control Office, Tri-Service General Hospital, National Defense Medical Center, Taipei, Taiwan; 11 Department of Pediatrics, Tri-Service General Hospital, National Defense Medical Center, Taipei, Taiwan; 12 Faculty of Nursing, Chiang Mai University, Chiang Mai, Thailand; 13 Quality management division in Hung Vuong hospital, Ho Chi Minh, Vietnam; 14 Infection control department in Hung Vuong Hospital, Ho Chi Minh, Vietnam

## Erratum

After publication of this supplement abstract article [1], some errors in the published version [1] were reported and are included in this erratum.

Abstract PM4 was accidentally withdrawn during the processing of the supplement when it should not have been. The Publisher apologises for this error and inconveniences caused. PM4 has been included in full this erratum.

In the abstract AR11, “3 MRGN” and “4 MRGN” were incorrectly included as “MGRN” in the ‘Material and methods’ section. This has been corrected in the abstract in this erratum.

In abstract AR12, “95% CI” was incorrectly referred to as “95% C.I”. A point should not have been included between “C” and “I”. This has been corrected in the abstract included in this erratum.

In abstract E6, “aims“ was incorrectly indicated as “aim” and "of the study is" should not have been included in the ‘Background’ section. These have been corrected in the abstract in this erratum.

In the abstract PM1, “Kirby-Bauer” was incorrectly referred to as “Kirby-bauer” in the ‘Materials and methods’ section. The correct presentation of this is included in the abstract in this erratum.

In abstract PM6, “pathogens” and “vancomycin” were capitalised incorrectly in the ‘Background’ and ‘Materials and methods’ section. These have been corrected in the abstract in this erratum.

In abstract PM12, Nattawipa Boonkirdram’s Family Name was incorrectly presented as “boon kirdram”. This has been corrected in the abstract in this erratum.

In abstract P27, “coagulase-negative staphylococci” was incorrectly italicised. This has been corrected in the abstract in this erratum.

## Session: Prevention of MDROs

### PM4 Octenidine to fight *Staphylococcus aureus* epidemic clones: an alternative strategy

#### Teresa Conceição^1^, Hermínia de Lencastre^1,2^, Marta Aires-de-Sousa^3^

##### ^1^Laboratory of Molecular Genetics, Instituto de Tecnologia Química e Biológica António Xavier, Universidade Nova de Lisboa, Oeiras, Portugal; ^2^Laboratory of Microbiology and Infectious Diseases, The Rockefeller University, New York, NY, USA; ^3^Escola Superior de Saúde da Cruz Vermelha Portuguesa, Lisboa, Portugal

###### **Correspondence:** Marta Aires-de-Sousa (msousa@esscvp.eu)

**Background**

Mupirocin nasal ointment, together with chlorhexidine-based skin disinfectants, have been used for methicillin resistant *Staphylococcus aureus* (MRSA) decolonization, but emergence of resistance strains highlights the need for alternative strategies. In the present study, we investigated the bactericidal activity of octenidine among international epidemic clones of *S. aureus*, including mupirocin resistant strains.

**Materials and methods**

Eight *S. aureus* isolates belonging to different epidemic clones were included. Mupirocin resistance was tested by disk diffusion, E-test and by PCR amplification of *mup*A and *mup*B. The bactericidal efficacy of octenidine was determined with and without organic load (albumin) by quantitative suspension tests according to EN13727.

**Results**

Two MRSA isolates showed high-level mupirocin resistance (MIC > 1024 mg/L and *mup*A), two MRSA isolates showed low-level mupirocin resistance (MICs = 24 and 32 mg/L) and the two remaining MRSA and the two MSSA were susceptible to mupirocin (MICs = 0.25 to 0.75 mg/L). Octenidine was effective (bacterial reduction more than 6 log10) at a concentration of 0.001% (10 ppm) after 30 seconds of contact time for all isolates tested. With 0.6% albumin (corresponding to protein levels found in human nasal mucosa), bacterial reduction more than 6 log10 was observed with 0.01% octenidine (100 ppm) at the same contact time. The octenidine-based ready-to-use product, octenisept®, used in the same experimental setup was demonstrated to be highly effective (bactericidal reduction more than 6 log10 after 30 seconds).

**Conclusions**

Octenidine was a promising alternative for *S. aureus* eradication, including mupirocin resistant strains belonging to different epidemic clones, and at significantly lower concentrations than those currently used in the clinical setting (0.05% to 0.1%).

## Session: Antimicrobial Resistance

### AR11 Antimicrobial activity of octenidine against multidrug-resistant gram-negative pathogens

#### Rocio Alvarez Marin^1,2^, Marta Aires de Sousa^3^, Nicolas Kieffer^1^, Patrice Nordmann^1,4^, Laurent Poirel^1^

##### ^1^Medical and Molecular Microbiology Unit, Department of Medicine, University of Fribourg, Fribourg, Switzerland; ^2^Hospital Universitario Virgen del Rocio y Virgen Macarena, Seville, Spain; ^3^Escola Superior de Saude da Cruz Vermelha Portuguesa, Lisbon, Portugal; ^4^University of Lausanne and University Hospital Center, Lausanne, Switzerland

###### **Correspondence:** Laurent Poirel (laurent.poirel@unifr.ch)

**Background**

Multidrug-resistant gram-negative (MRGN) pathogens pose a major and growing threat for health care systems, as therapy of infections is often limited due to the lack of available systemic antibiotics. Well tolerated antiseptic molecules may be a very useful implementation in infection control,not only to reduce the dissemination of methicillin-resistant Staphylococcus aureus (MRSA), but also MRGN.

**Material and methods**

As decolonization strategies with regard to MRSA are already implemented in high risk areas (i.e. ICUs), this study aimed to investigate, if the same protocol might be concomitantly efficient against MRGN. A series of 5 different species (*Escherichia coli, Klebsiella pneumoniae, Enterobacter cloacae, Acinetobacter baumannii, Pseudomonas aeruginosa*) was studied to prove efficacy under clinically relevant conditions according to an official test norm (EN13727). We used 5 clonally-unrelated isolates per species, including a single wild-type strain, and four MRGN isolates, corresponding either to the 3 MRGN or 4 MRGN definition of multidrug resistance. Octenidine (OCT, Schuelke&Mayr GmbH, Germany) susceptibility was evaluated with and without organic load.

**Results**

A contact time of 30 seconds or 1 minute was fully effective for all isolates by using different OCT concentrations (0.01% and 0.05%), with a bacterial reduction factor of >5 log systematically observed. Growth kinetics were determined with two different wild-type strains (*A. baumannii and K. pneumoniae*), proving a time-dependent efficacy of OCT, mirroring what has been previously observed for MRSA.

**Conclusions**

These results highlight that OCT, besides being a very effective agent against MRSA, may also be extremely useful to eradicate emerging highly resistant gram-negative pathogens associated with nosocomial infections.

## Session: Antimicrobial Resistance

### AR12 Comparison of mortality of patients with *Acinetobacter baumannii* bacteremia caused by different levels of drug resistance

#### Wison Laochareonsuk, Sireekul Petyu, Pawin Wanasitchaiwat, Sutasinee Thana, Chollathip Bunyaphongphan, Woranan Boonsomsuk, Pakpoom Maneepongpermpoon, Silom Jamulitrat

##### Prince of Songkla University, Hat Yai, Songkla, Thailand

###### **Correspondence:** Wison Laochareonsuk (ic_conference@yahoo.com)

**Background**

*Acinetobacter baumannii* is an important opportunistic nosocomial pathogen causing a variety of infections. The intrinsic virulence of drug-resistant *A. baumannii* has remained controversial. We compared mortality rates and sepsis score of patients with *A. baumannii* bacteremia caused by different level of drug resistance.

**Materials and methods**

A retrospective study was conducted in adult patients (age > 15 years) admitted to Songklanagarind hospital during 2009 and 2015 and blood culture positive for *A. baumannii* after 3 days of admission. Antimicrobial resistance was categorized into four levels comprising of non-multidrug resistance (non-MDR), multidrug-resistant (MDR), extensively drug-resistant (XDR), and possible pandrug-resistant (possible-PDR). Severity of underlying disease of the patients immediately before onset of bacteremia was determined by sequential organ failure (SOFA) score and American Association of Anesthesia (ASA) score. Virulence of *A. baumannii* was assessed in terms of sepsis score and in hospital mortality rate.

**Results**

The study identified 38, 110, 168, and 14 cases of bacteremia caused by non-MDR, MDR, XDR, and possible PDR, respectively. After adjusting for confounding effect by using Cox proportional hazard model, mortality rates attributable to *A. baumannii* was significantly associated to levels of drug resistance. Using nonMDR as a reference, the incidence rate ratios and corresponding 95% confidence intervals (95% CI) of MDR, XDR, and possible PDR were 2.3 (95% CI = 0.9-4.9), 3.1(95% CI = 1.4-7.0), and 1.9(95% CI = 0.6-5.5) respectively.

**Conclusions**

The virulence of *A. baumannii* did not loss with drug resistance.

## Session: Environment control

### E6 Garnering staff towards a cleaner and safer environment: the national kidney foundation experience

#### Jamilaf Binte Jantan^1^, Chua Chor Guek^1^, Eu Chiow Kian^1^, Pampe Anak Pirido^1^, Nur Fadilah Binte Mohd Aron^2^, Leah May Estacio^3^, Francis Alvarez Palana^4^, Michelle Gracia^5^, Nur Syafiqah Binte Shamsuddin^6^, Kersten Timbad Castro^7^, Madonna Baloria^8^, Faezah Binte Adam^9^

##### ^1^Nationa kidney foundation, Singapore, Singapore; ^2^Jurong West1 Dialysis Centre, Singapore, Singapore; ^3^Kolam Ayer Dialysis Centre, Singapore, Singapore; ^4^Woodlands1 Dialysis Centre, Singapore, Singapore; ^5^Tech Whye Dialysis Centre, Singapore, Singapore; ^6^Yishun1 Dialysis Centre, Singapore, Singapore; ^7^Hougang1 Dialysis Centre, Singapore, Singapore; ^8^Kim Keat Dialysis Centre, Singapore, Singapore; ^9^Tampines2 Dialysis Centre, Singapore, Singapore

###### **Correspondence:** Jamilah Binte Jantan (jamilah.jantan@nkfs.org)

**Background**

In the dialysis centre, there is potential for cross transmission of infectious agents through contaminated devices, hands, equipment, supplies and environmental surface during haemodialysis (HD) treatment. To reduce the risk of acquiring infections, staff routinely clean and disinfect medical equipment and high-touch areas after each patient's HD treatment at the National Kidney Foundation (NKF). This study aims to assess environmental cleaning of high-touch areas and develop intervention program to achieve compliance ≥85%.

**Materials and methods**

This is a quantitative study involving 29 Infection Control Link Nurses (ICLNs) at the Community-based Dialysis Centres (CB-DCs), NKF from October 2015 to April 2016. In November 2015, ICLNs conducted an environmental cleaning assessment of high-touch areas using a checklist and Glo Germ Kits, to ascertain the efficacy of environmental cleaning at 29 CB-DCs. Pre-study data showed an overall average of 67% compliance. RCA revealed the absence of an audit tool for high-touch areas, a lack of training leading to knowledge deficit, poor cleaning techniques and staff incompetency. Interventions included a checklist (audit tool) for environmental cleaning assessment of high-touch areas, a "Train the Trainer" programme for the 29 ICLNs, an annual competency assessment and video tutorials on environmental hygiene to standardise practice.

**Results**

Following the interventions, environmental cleaning assessment of high-touch areas showed an overall average of 86% compliance, with 17 CB-DCs achieving ≥85% compliance in environmental cleaning of high-touch areas.

**Conclusions**

This study illustrated that the intervention programme increased staff awareness, thereby improving compliance. Besides promoting positive outcomes, it enhanced the internal monitoring system at NKF.

## Session: Prevention of MDROs

### PM1 Successful control of carbapenem-resistant Enterobacteriaceae outbreaks

#### Terrence Rohan Chinniah, Kavitha Prabu, Rashidah Ahmad, Susylawathi Magon, Jauharatud DiniSuhaimi, Aizzuddin Mirasin, Nurul Morni, Boon Chu, Azizah Samsuddin, Aliyah Ahmad, Amalina Sidek, Noraini Ajis, Amalina AbuBakar, Amanie Shafiee, Julaini Safar

##### Raja Isteri Pengiran Anak Saleha Hospital, Bandar Seri Begawan, Brunei Muara, Brunei Darussalam

###### **Correspondence:** Terrence Rohan Chinniah (trctrc64@gmail.com)

**Background**

Infection with Carbapenem-resistant Enterobacteriaceae (CRE) is increasing worldwide leading to high morbidity and mortality. No CRE cases were detected in Brunei till April 2013. This study aimed to control the outbreak as soon as it’s detected by microbiology laboratory.

**Materials and methods**

All specimens at microbiology laboratory are cultured according to routine laboratory methods and identified by VITEK 2, VITEK MS, API 20E and API 20NE. Antibiotic susceptibility test is performed according to Clinical Laboratory Standard Institute (CLSI) guidelines. Enterobacteriaceae showing resistant to carbepenems, namely ertapenem, imipenem and meropenem, were rechecked by VITEK 2 XL, Kirby-Bauer method and Minimum Inhibitory Concentration by Estrips. If it still remain resistance to carbapenem, clinicians and infection control team was alerted immediately by email and phone. All these were found to be carbapenemase producing CRE, by Modified-Hodge test recommended by CLSI.

**Results**

One case of CRE was identified in April 2013. No case was detected during the year of 2014. From March 2015 till October 2015, sixteen cases of CRE were confirmed. Another small outbreak of four cases was noted from January 2016 till March 2016. No cases were identified since then.

**Conclusions**

Following identification of one case in April 2013 barrier nursing and isolation were re-visited to seal spread from this case. Outbreak of 15 cases in 2015 was mainly among critical care patients. Due to vigorous infection control measures this outbreak was control. Similarly the smaller outbreak of 5 cases in 2016 was also controlled by vigorous infection control practices. This experience of ours underlines the importance of early detection of CRE and strict infection control measures.

## Session: Prevention of MDROs

### PM6 The influence of disease burden for patients infected with multidrug-resistant pathogens

#### Ming-Chin Chan^1^, Chih-Chien Wang^2^

##### ^1^Infection Control Office, Tri-Service General Hospital, National Defense Medical Center, Taipei, Taiwan; ^2^Department of Pediatrics, Tri-Service General Hospital, National Defense Medical Center, Taipei, Taiwan

###### **Correspondence:** Chih-Chien Wang (jmj621115@gmail.com)

**Background**

Since the use of antibiotics, drug-resistant pathogens were inevitably appeared. It is undoubtedly a huge challenge to clinical practices. Infections with multidrug-resistant pathogens would result in higher mortality rate and extra medical expenses.

**Materials and methods**

This research collected and analyzed healthcare-associated bloodstream infection by multi-drug resistant pathogens including *Acinetobacter baumannii, Pseudomonas aeruginosa*, vancomycin-resistant enterococci (VRE), *Staphylococcus aureus* and *E. coli* in Tri-Service General Hospital between 2011 and 2014. The cases were collected by infection control nurses. All the information and the expenditure of ICUs or wards were analyzed and compared.

**Results**

The results showed the highest mortality was VRE, about 80% and 57% of mortality rate in ICUs and wards, respectively. The longest days from admission to infection was *Staphylococcus aureus*, about 55.4 and 74.5 days in ICU and ward, respectively. Moreover, pathogen that has the longest days from infection to outpatient or expired was *E. coli*, with the average of 59.6 and 41.6 days in ICUs and wards, respectively. As for the expenditure, patients infected with *E. coli* in ICUs, and patients infected with VRE in wards cost the most expenses, which was about 15,333 and 5,000 US dollars, respectively.

**Conclusions**

The expenditure for nosocomial infections with multi-drug resistant pathogens in ICUs is higher than in wards, especially infected with *E. coli*, the other four pathogens caused about 6,333-10,000 US dollars in ICUs. In wards, it is about 3,667-4,667 US dollars infected with VRE, *Staphylococcus aureus* and *E. coli*. The antibiotics cost is the highest when infected with VRE.

## Session: Prevention of MDROs

### PM12 Development of clinical pathway for prevention and control of multidrug-resistant organisms transmission in hospital

#### Nattawipa Boonkirdram, Wilawan Picheansathian, Pimpaporn Klunklin

##### Faculty of Nursing, Chiang Mai University, Chiang Mai, Thailand

###### **Correspondence:** Nattawipa Boonkirdram (spun.11@hotmail.com)

**Background**

Transmission of multidrug-resistant organisms in hospitals has a direct impact on patients, health care personnel, the hospital, community, and the nation. This developmental research aimed to develop a clinical pathway for prevention and control of multidrug-resistant organism transmission in the medical department at a regional hospital.

**Materials and methods**

Study samples included 131 health care personnel who worked in the medical department and related including: hemodialysis, echocardiogram, radiology and ultrasound, CT scan, MRI, and stretcher. Ten patients infected or suspected of multidrug-resistant organism infection at the medical department were included in the test. The process for developing was based on Cheah’s (2000) framework. Three development steps were thus included: assessment and situation analysis, designing, and testing the implementation. The data collection instruments consisted of a demographic data record form and questionnaire assessing the opinions of health care personnel towards. The content validity of the questionnaire was examined by 5 experts and the content validity index was 0.90. Data were analyzed using descriptive statistics and data categorization.

**Results**

The results included: screening for multidrug-resistant organisms, using contact precautions, using masks when performing splash-generating procedures, cleaning patients’ bodies, cleaning the immediate environment, reminding health care personnel of best practices and proper transfer between units, active surveillance, and limiting medical errors and adverse events. Most health care personnel agreed that this clinical pathway was clear, convenient, feasible, practical and appropriate for implementation in their units.

**Conclusions**

This research suggests that should be routinely implemented in the hospital.

## Session: Prevention of Site Specific Infections

### P27 Surgical site infection after obstetric and gynecology surgery in Hung Vuong Hospital, Vietnam

#### Hang Thi Phan^1^, Anh Pham Phuong Dinh^2^, Tuyet Thi Kim Nguyen^2^

##### ^1^Quality management division in Hung Vuong hospital, Ho Chi Minh city, Viet Nam; ^2^Infection control department in Hung Vuong Hospital, Ho Chi Minh city, Viet Nam

###### **Correspondence:** Anh Pham Phuong Dinh (thuyhangytcc@gmail.com)

**Background**

Surgical site infection (SSI) is the most common hospital acquired infection at obstetrics and gynecology hospital. SSI prolonged hospital stay and increased treatment costs. We aimed to identify incidence and causative organism of surgical site infection among cesarean section and gynecology surgery cases.

**Materials and methods**

A prospective cohort study was conducted at Hung Vuong Hospital, Viet Nam from January 2015 to May 2016. Women who underwent caesarean and gynecological surgery were included in the study. CDC criteria (2009) were used to diagnose SSI.

**Results**

29,681 women underwent caesarean and gynecological surgery. SSI rate was 1.1% (95% CI: 0.98 – 1.22%), in which 0.96% SSI after caesarean section (95% CI: 0.84 – 1.08%) and 1.7% SSI after gynecological surgery (6.26% SSI after hysterectomy with 95% CI: 4.91 – 7.79%). Average number of treatment days that with SSI is 9 ± 4.5 days. *Staphylococcus aureus* and coagulase-negative staphylococci were the most common organism caused SSI after caesarean section (50%) and *Escherichia coli* was the main organism for SSI after gynecology operation (66.7%).

**Conclusions**

The incidence of SSIs was limited because we only follow patients with SSIs who were treated in hospital. The incidence of SSIs after hysterectomy surgery was higher than other types of gynecologic surgery. It’s necessary to promote infection control to reduce SSI in hysterectomy surgery.
